# Transcriptional network orchestrating regional patterning of cortical progenitors

**DOI:** 10.1073/pnas.2024795118

**Published:** 2021-12-17

**Authors:** Athéna R. Ypsilanti, Kartik Pattabiraman, Rinaldo Catta-Preta, Olga Golonzhka, Susan Lindtner, Ke Tang, Ian R. Jones, Armen Abnousi, Ivan Juric, Ming Hu, Yin Shen, Diane E. Dickel, Axel Visel, Len A. Pennacchio, Michael Hawrylycz, Carol L. Thompson, Hongkui Zeng, Iros Barozzi, Alex S. Nord, John L. Rubenstein

**Affiliations:** ^a^Nina Ireland Laboratory of Developmental Neurobiology, Department of Psychiatry, UCSF Weill Institute for Neurosciences, University of California, San Francisco, CA 94158;; ^b^Department of Neurobiology, Physiology and Behavior, University of California, Davis, CA 95618;; ^c^Department of Psychiatry and Behavioral Sciences, University of California, Davis, CA 95618;; ^d^Precise Genome Engineering Center, School of Life Sciences, Guangzhou University, Guangzhou 510006, China;; ^e^Institute for Human Genetics, University of California, San Francisco, CA 94143;; ^f^Department of Neurology, University of California, San Francisco, CA 94143;; ^g^Department of Quantitative Health Sciences, Lerner Research Institute, Cleveland Clinic Foundation, Cleveland, OH 44195;; ^h^Environmental Genomics and Systems Biology Division, Lawrence Berkeley National Laboratory, Berkeley, CA 94720;; ^i^School of Natural Sciences, University of California, Merced, CA 95343;; ^j^US Department of Energy Joint Genome Institute, Berkeley, CA 94720;; ^k^Comparative Biochemistry Program, University of California, Berkeley, CA 94720;; ^l^Allen Institute for Brain Science, Seattle, WA 98109;; ^m^Faculty of Medicine, Department of Surgery and Cancer, Imperial College, London SW7 2AZ, United Kingdom

**Keywords:** cortical patterning, epigenetics, transcription factors, progenitor cells

## Abstract

We uncovered a transcription factor (TF) network that regulates cortical regional patterning in radial glial stem cells. Screening the expression of hundreds of TFs in the developing mouse cortex identified 38 TFs that are expressed in gradients in the ventricular zone (VZ). We tested whether their cortical expression was altered in mutant mice with known patterning defects (*Emx2, Nr2f1*, and *Pax6*), which enabled us to define a cortical regionalization TF network (CRTFN). To identify genomic programming underlying this network, we performed TF ChIP-seq and chromatin-looping conformation to identify enhancer–gene interactions. To map enhancers involved in regional patterning of cortical progenitors, we performed assays for epigenomic marks and DNA accessibility in VZ cells purified from wild-type and patterning mutant mice. This integrated approach has identified a CRTFN and VZ enhancers involved in cortical regional patterning in the mouse.

## Significance

Development of cortical areas begins in cortical stem cells through the action of morphogens controlling the graded expression of transcription factors (TFs). Here, we have systematically identified the TFs and gene regulatory elements (REs) that together control regional pattering of the cortical progenitor zone; these data have led us to propose a cortical regionalization TF network. To identify REs active in this network, we performed TF chromatin immunoprecipitation followed by sequencing (ChIP-seq) and chromatin-looping conformation experiments as well as assays for epigenomic marks and DNA accessibility in purified ventricular zone (VZ) progenitor cells in wild-type and patterning mutant mice. This integrated approach has laid the foundations to identify a TF network and cortical VZ REs involved in cortical regional patterning.

Cortical development is an intricate choreography that integrates finely tuned molecular states such as gradients of transcription factors (TFs) and the chromatin landscape with a variety of cellular processes (cell type specification, proliferation, differentiation, and migration). Together, these developmental processes result in the establishment of an adult cerebral cortex and associated pallial structures, which are organized into discrete areas and laminae distinguished by specific molecular signatures (1). The earliest steps in the generation of cortical areas (regionalization process) are regulated in mouse by fibroblast growth factor signaling and by TFs such as ARX, DMRT3, DMRT5, EMX2, LEF1, LHX2, NR2F1, NR2F2, PAX6, PBX1, and SP8; TFs which exhibit graded expression patterns along the rostral/caudal and dorsal/ventral axes in the neuroepithelium and radial glial stem cells of the developing pallium (2–6). These TFs are crucial for early patterning of the cortex, and their loss causes shifts in cortical regional structures (7–10). As the cortex develops, regional identity is then translated from the ventricular zone (VZ) to secondary progenitors of the subventricular zone and then to neurons of the cortical plate (11).

To understand cortical patterning, one needs to comprehend the regulatory architecture of individual genes that play a role in regionalization. Work on the regulatory control of individual TF genes identified enhancer elements that control gene expression of specific patterning TFs such as *Emx2* (12, 13) and *Pax6* (14–16). A larger-scale effort identified E14.5 pallial enhancers by chromatin immunoprecipitation and DNA sequencing (ChIP-Seq) for p300 (an enhancer-associated coactivator) and showed activity for eight enhancers (17). Similarly, we performed a large-scale identification of forebrain putative regulatory elements (pREs) and found 145 pREs active in the forebrain at E11.5 (18). This work showed that many cortical pREs had sharp boundaries of activity in the neuroepithelium. In a follow-up study, we discovered that TF binding at individual pREs enabled them to integrate information from TF gradients in the developing pallium to generate discrete regional domains prenatally and postnatally (19). Individual enhancers with discrete neuroepithelial activity can together encode regional expression gradients, and thus individual pRE activity may reflect the highest resolution of transcriptional differences within the developing cortex and serve as a protomap of cortical areas (20).

**Fig. 1.**
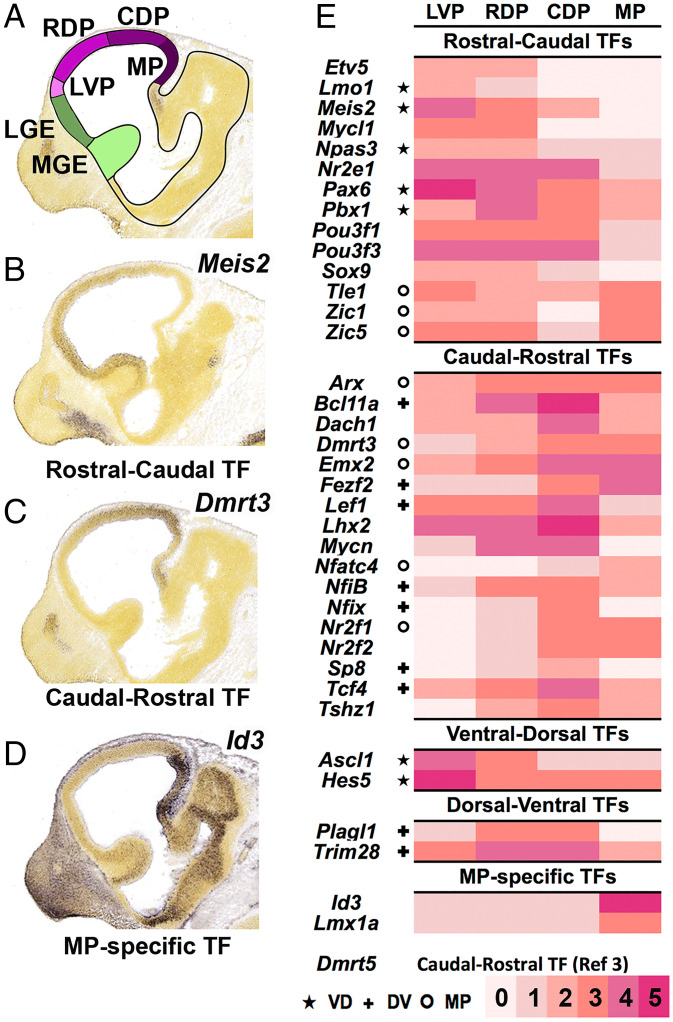
Annotation of TF expression in the CRTFN in the E11.5 cortex. (*A*) Schema of sagittal view of E11.5 mouse brain. The pallium is in four shades of purple corresponding to regional subdivisions (LVP: laterovental pallium, RVP: rostrovental pallium, CDP: caudodoral pallium, MP: medial pallium). (*B–D*) ISH analysis of *Meis2* (*B*), *Dmrt3* (*C*), and *Id3* (*D*). (*E*) Heatmap of cortical expression levels (0 to 5: pink to red scale) of CRTFN TFs in the four pallial subdivisions. ★ indicates that the TF also has VD gradient. ✚ indicates that the TF also has DV gradient. ^o^ indicates that the TF also has MP expression.

Herein, we built on our previous work with the aim to elucidate components of the transcriptional network that orchestrates regionalization of the cortical neuroepithelium. We focused on 38 cortical progenitor TFs that have graded RNA distribution along the rostrocaudal (RC) and/or dorsoventral (DV) axes. We refer to these 38 TFs as the cortical regionalization TF network (CRTFN) (Fig. 1*E*). Toward defining the transcriptional logic of cortical patterning, we assessed changes in CRTFN expression in the context of known cortical patterning defects by using *Emx2*, *Nr2f1*, and *Pax6* loss-of-function mouse mutants. We found that rostroventral patterning involves *Pax6* promoting the expression of *Nr2f1* and *Nr2f2*, which in turn promotes *Lmo3* and *Npas3.* We show that loss of *Npas3* function also alters rostroventral cortical patterning.

To identify the genomic REs tying together the CRTFN, we performed TF ChIP-seq for EMX2, LHX2, NR2F1, PAX6, and PBX1. This analysis, coupled to histone ChIP-seq and assay for transposase-accessible chromatin with high-throughput sequencing (ATAC-seq), identified ∼2,000 pREs in the CRTFN. Combinatorial TFs binding was tightly associated with the activity of cortical enhancers. Furthermore, coordinated binding of EMX2, LHX2, NR2F1, PAX6, and PBX1 provided evidence for a combinatorial signature regulating enhancer activity along the rostroventral–caudodorsal axes. Finally, by assessing epigenetic changes of the 2,000 CRTFN pREs in *Emx2*, *Nr2f1*, and *Pax6* mutants, we identified enhancers likely to participate in cortical patterning. Together, this study establishes the foundations of the transcriptional circuitry operating in radial glial stem cells and underlying cortical regionalization.

## Results

### Pallial Expression of 698 TFs at E11.5.

To systematically identify TFs that could participate in cortical regional patterning through their activity in neural progenitors, we used the Allen Brain Developing Mouse Atlas to study the RNA expression of 698 TFs based on in situ hybridization (ISH) analyses on sagittal sections of E11.5 mouse embryos.

We detected pallial expression for 270 TFs but did not observe pallial expression for another 428 TFs. We described the pallial expression of all TFs based on their regional and laminar expression patterns using topological schemata that define pallial expression in lateroventral, rostrodorsal, caudodorsal, and medial pallial regions (LVP, RDP, CDP, MP, respectively) (Fig. 1*A* and Dataset S1). Herein, we focus on progenitor zone expression, as we wished to concentrate on mechanisms that operate in progenitors and control pallial regional patterning. We assessed the level of expression based on both the intensity and the density of expression (0 to 5 scale). Fig. 1*E* shows TFs that were expressed in gradients in progenitors at E11.5 in the LVP, RDP, CDP, and MP. In this analysis, we identified 218 TFs whose telencephalic expression was specific to the VZ of the pallium and/or showed a regional or graded pattern within the pallium, with an expression intensity of 2 or more. Only a few TFs had regional expression boundaries: 10 largely restricted to the DP, 6 to the MP (i.e., *Id3*; Fig. 1*D*), and 1 to the LVP. More commonly, TFs were broadly expressed across the pallium (*n* = 134), with many showing either RC (*n* = 30 [i.e., *Meis2*]; Fig. 1*B*) or caudorostral (CR, *n* = 31 [i.e., *Dmrt3*]; Fig. 1*C*) gradients.

We hypothesize that our screen and subsequent analysis identified most of the TFs that are candidate VZ regulators of pallial patterning at E11.5. We will refer to these TFs as the CRTFN. This screen also provides evidence that there are few TFs with restricted intrapallial expression domains except in the MP, supporting the idea that most pallial subdivisions are not generated by highly restricted TF expression within pallial progenitors.

### *Emx2*, *Nr2f1*, and *Pax6* Regulate Gradients of CRTFN Expression at E11.5.

**Fig. 2.**
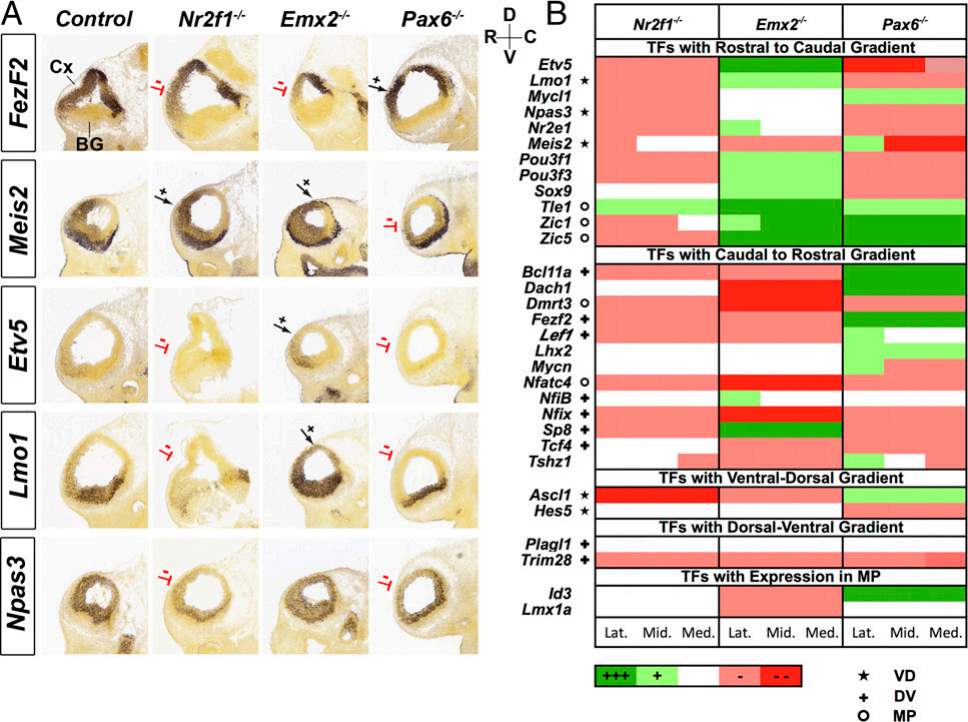
Graded expression changes of CRTFN TFs in *Nr2f1*, *Emx2*, and *Pax6* mutants. *(A)* ISH shows changes in one CR TF (*Fezf2)* and four RC TFs (*Meis2*, *Etv5*, *Lmo1*, and *Npas3)* in E11.5 sagittal sections of *Nr2f1*, *Emx2*, and *Pax6* mutants (*n* = 2). Red lines denote reduced expression, whereas black arrows indicate increased expression. The RC and DV axes are depicted on the *Top Right* of the sections. (*B*) Heatmap showing changes in expression of 31 TFs in *Nr2f1^−/−^*, *Emx2^−/−^*, and *Pax6^−/−^* using a five-level qualitative scale: increased expression (dark green, +++ or light green, +); no change (white); decreased expression (light red − or dark red − −). TFs are categorized according to their gradient in WT (RC, CR, VD, or DV) or their expression in a restricted region (MP). Changes are annotated in Lateral (Lat.), Middle (Mid.), and Medial (Med.) sagittal sections. ★ indicates that the TF also has VD gradient. ✚ indicates that the TF also has DV gradient. ^o^ indicates that the TF also has MP expression.

Next, we investigated how CRTFN TFs are regulated by TFs with known functions in cortical patterning. We studied the expression of 31 TFs in *Emx2*, *Nr2f1*, and *Pax6* mouse mutants at E11.5 (Fig. 2 and *SI Appendix*, Fig. S6). We chose to study the 31 TFs based on the following three criteria: 1) TFs previously not well-known to be expressed in the E11.5 pallium; 2) TFs whose neurodevelopmental functions were poorly known; and 3) TFs with clear E11.5 expression gradients.

The phenotypic descriptions of the effects of the *Emx2*, *Nr2f1*, and *Pax6* mutations on the expression of the 31 TFs are organized based on changes of their RC, CR, or regional expression. We annotated the expression changes based on the independent assessment of three experts using a five-level qualitative expression scale: increased expression (green; ++ or +); no change; decreased expression (red; – − or −) (Fig. 2*B*). The expression changes were assessed at lateral, middle, and medial levels of sagittal sections at E11.5, and the expression change at each level was denoted in Fig. 2. Representative sections for each TF in the three mutants can be found in *SI Appendix*, Fig. S6. All but one TF (*Plagl1*) showed differential RNA expression in at least one of the three mutants.

During cortical patterning, EMX2 and NR2F1 promote caudal identity, whereas PAX6 promotes rostral identity (2, 21). Considering the regional patterning role of these TFs, we hypothesized that genetic ablation would lead to loss of regional transcriptional activation along the rostral–caudal axis. Consistent with this, genes with CR gradients (i.e., increased caudal expression relative to rostral) were more likely to show decreased expression in *Emx2^−/−^* (9/13) and in *Nr2f1^−/−^* (8/13) than in *Pax6^−/−^* (5/13). Likewise, genes with RC gradients showed reduced expression in *Pax6^−/−^* (8/12) and increased expression in *Emx2^−/−^* (9/12) (Fig. 2 and *SI Appendix*, Fig. S6). However, unlike in *Emx2^−/−^*, loss of *Nr2f1* led to a decrease in expression of RC TFs (9/12).

Although *Nr2f1* and *Emx2* are expressed in CR gradients, they have opposite DV gradients. *Emx2* has a DV gradient, whereas *Nr2f1*, like *Pax6*, has a ventrodorsal (VD) gradient (*SI Appendix*, Fig. S2*A*). Thus, perhaps the TF expression differences between the *Emx2* and *Nr2f1* mutants might in part relate to their differences in DV patterning functions. Indeed, in the *Nr2f1* knockout, several down-regulated RC TFs (including *Npas3*) were also down-regulated in the *Pax6* mutant (Fig. 2*B*). This led us to explore whether *Pax6 and Nr2f1* have similar roles in regulating the ventral patterning of the cortex.

### *Nr2f1/2* and *Pax6* Regulate Patterning of the Rostral Ventrolateral Pallium.

**Fig. 3.**
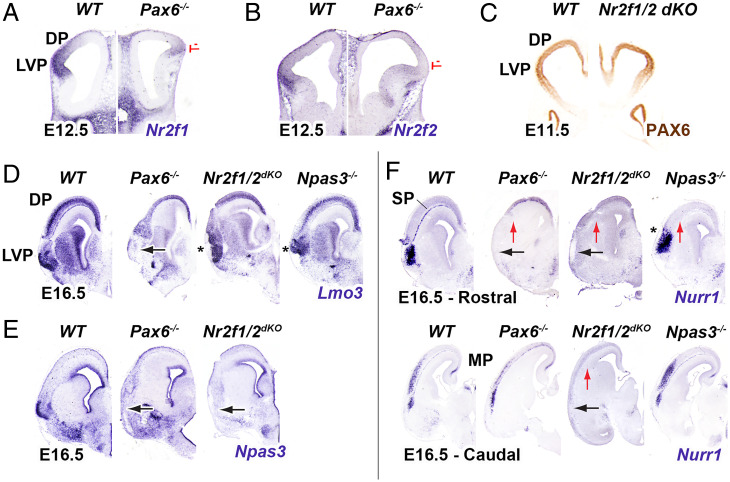
*Pax6*-*Nr2f1/2*-*Npas3* pathway promotes patterning of the rostral latero-ventral pallium and subplate. (*A*, *B*) At E12.5, *Nr2f1 and Nr2f2* ISH expression is decreased in the LVP and DP of coronal sections in *Pax6^−/−^* (red lines, *n* = 3). (*C*) At E11.5, PAX6 immunohistochemistry shows no change in the *Nr2f1/2* dKO (*n* = 2). (*D–F*) E16.5, ISH shows changes of LVP markers (*Lmo3, Npas3* and *Nurr1)* in *Pax6^−/−^* (*n* = 3), *Nr2f1/2* dKO (*n* = 2) and *Npas3^−/−^* (*n* = 3) in rostral sections. Caudal sections are also depicted for *Nurr1* ISH. Black arrow: Loss of expression in the R LVP; Star (*): abnormal morphology of the R LVP; Red arrow: loss of the subplate (SP).

The loss of function experiments described in the previous section (Emx2*,* Nr2f1*, and* Pax6* Regulate Gradients of CRTFN Expression at E11.5*) suggest that TFs with rostral-ventral gradients (such as *Npas3*) are similarly regulated by *Pax6* and *Nr2f1.* Loss-of-function analyses show that *Pax6* has prominent roles in promoting LVP properties (22). In *Pax6^−/−^* we found that LVP expression of both *Nr2f1* and *Nr2f2* were strongly reduced at E12.5 (Fig. 3 *A* and *B*), suggesting that patterning of the ventral pallium by *Pax6* involves *Nr2f1* and *Nr2f2*. Previous work did not find LVP defects in *Nr2f1^−/−^* (23). However, because *Nr2f1* and *Nr2f2* expression are very similar, they may compensate for each other. Therefore, we analyzed *Nr2f1/2* conditional mutants (cKOs; *Nr2f1^−/−^; Nr2f2^f/f^; Emx1-cre*). We first assessed whether they coregulated *Pax6* expression in the LVP at E11.5 but detected no change in PAX6 immunostaining (Fig. 3*C*). We then examined rostroventral cortex phenotypes at later ages in both *Pax6^−/−^* and *Nr2f1/2* cKOs. In the rostroventral cortex at E16.5, ventral cortex markers, *Nurr1* and *Npas3*, were strongly reduced in both the *Pax6^−/−^* and *Nr2f1/2* cKOs. (Fig. 3 *E* and *F*). *Nurr1* expression was maintained ventrocaudally in the *Pax6*^−/−^ but was lost in the *Nr2f1/2* cKOs (Fig. 3*F*). Among other ventral cortex markers, *Lmo3* exhibited reduction in expression in *Pax6^−/−^* and not the *Nr2f1/2* cKOs (Fig. 3*D*). This suggests that *Pax6* can also work through an *Nr2f1/2*-independent pathway to specify the ventral cortex.

We next examined whether some of the rostroventral TFs that were coregulated by *Pax6* and *Nr2f1* could in turn regulate rostroventral cortical development. To that end, we examined the rostroventral cortical phenotypes in the *Npas3^−/−^* mice (24). We found that the rostroventral cortex in *Npas3*^−/−^ appeared dysmorphic based on *Lmo3* and *Nurr1* expression (Fig. 3 *D* and *F*). Moreover, as in *Pax6*^−/−^, *Npas3*^−/−^ maintained ventrocaudal *Nurr1* expression. The core components likely to participate in the transcriptional regulation of patterning the rostroventral cortex are schematized in *SI Appendix*, Fig. S1*A* (characterization of *Nr2f1*, *Npas3*, and *Lmo3* genomic loci in *SI Appendix*, Fig. S1*C* will be explained hereafter).

### Analysis of EMX2, LHX2, NR2F1, PAX6, and PBX1 Binding to Genomic Regions Associated with the CRTFN.

**Fig. 4.**
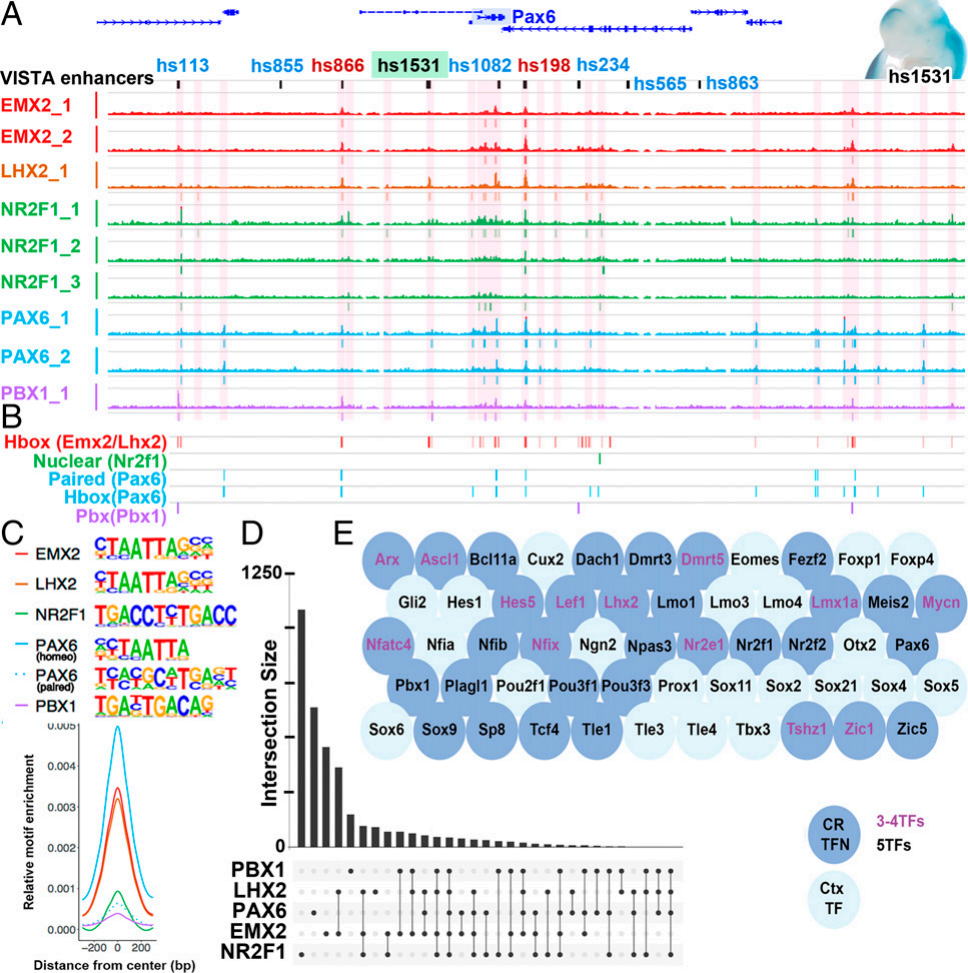
Combinatorial binding of EMX2, LHX2, NR2F1, PAX6, and PBX1 predicts genes in the CRTFN. (*A*) EMX2, LHX2, NR2F1, PAX6, and PBX1 ChIP-seq coverage at the *Pax6* locus at E12.5. pREs are highlighted in pink. VISTA enhancers are also indicated in green (cortical activity [i.e., hs1531, wholemount with positive blue LacZ staining, *Top Right*]), in blue (noncortical activity [i.e., hs113]), and in red (no activity at E11.5 [i.e., hs198]). (*B*) Genomic location of the EMX2, LHX2, NR2F1, PAX6, and PBX1 primary binding motifs in the *Pax6* locus. (*C*) Centered distribution of EMX2, LHX2, NR2F1, PAX6, and PBX1 motifs across ChIP-seq peaks. Motifs that were strongly enriched within ChIP peaks were homeobox motifs in EMX2, LHX2, PAX6, and PBX1, the paired motif in PAX6 and the nuclear receptor motif in NR2F1. Primary DNA binding sequences are shown. (*D*) Number of peaks for each TF alone or in combination. *y* axis shows number of peaks. *x* axis indicates single and combinatorial binding at pREs. (*E*) pREs with combinatorial binding of three or four (Purple letters) or five TFs (Black letters) are enriched around genomic loci for TFs from the CRTFN (blue circles, 30/35 TFs) as well as other TFs important in cortical development (light blue circles).

Next, we investigated whether PAX6, EMX2, and NR2F1 proteins bind to regulatory regions of the CRTFN genes (Dataset S2). Our goal was to determine whether they directly regulate these genes and to identify pREs for the CRTFN. We performed ChIP-seq using PAX6-, EMX2-, and NR2F1-specific antibodies on E12.5 cortical tissue (19) and here. We also chose to include ChIP-seq data for two other patterning cortical TFs that are expressed in progenitor cells, namely LHX2 and PBX1 (6), because the mutants for these TFs exhibit patterning defects (6, 25, 26). These five TFs are expressed in gradients in the pallial VZ: RC for *Pax6* and *Pbx1* and CR for *Emx2*, *Lhx2*, and *Nr2f1* (*SI Appendix*, Fig. S2*A*). A description of the ChIP-seq for PAX6, PBX1, and NR2F1 was previously published (6, 19). The LHX2 antibody was previously used for ChIP-seq (27), and a comparable LHX2 ChIP-seq on cortex at E12.5 was previously published (28). The EMX2 antibody was generated for this study; its specificity was tested by immunostaining in wild-type (WT) mice by performing ChIP-seq with an EMX2 blocking peptide (EMX2 Rep2, Fig. 4*A*) as well as by ChIP-qPCR in WT and *Emx2^−/−^* dissected cortices (*SI Appendix*, Fig. S2*B*). Representative ChIP-seq peaks are depicted around the *Pax6* locus in Fig. 4*A*. TF ChIP peaks are shown around the *Pax6* locus, and called peaks are annotated beneath the genomic tracks. Overlapping TF ChIP-seq peaks were merged into discrete pREs (highlighted in pink). We assessed the reproducibility of the ChIP-seq experiments by pairwise Pearson genome-wide read count correlation of biological replicates run individually, as depicted in *SI Appendix*, Fig. S2*D*. For each TF, ChIP binding sites were located predominantly at distal rather than proximal genomic loci (*SI Appendix*, Fig. S2*C*). We defined the size of each genomic locus by defining its boundaries by 1) the distal point of contact for promoter–enhancer looping obtained by a chromatin conformation assay (see *Promoter–Enhancer Binding around 35 TFs of the CRTFN*) 2) ±100 kb from the limits of the gene body.

We used Homer (29) to perform de novo motif discovery and enrichment analysis. We identified enriched sequence motifs including the primary binding motifs for EMX2, LHX2, PAX6, and PBX1 (homeoboxes), NR2F1 (nuclear receptor), and PAX6 (paired domain) (Fig. 4*C*) (6, 30–33). These putative primary motifs were enriched in the center of pREs (*SI Appendix*, Fig. S2 *F* and *J*) supporting direct TF binding. Beyond the primary motifs associated with TFs profiled here, many additional motifs were enriched within pREs, including motifs for TFs that are crucial for cortical development (i.e., SOX motifs), as well as for basal ganglia development (i.e., NKX and DLX motifs) (*SI Appendix*, Fig. S2*G*). Notably, when NR2F1 was bound at loci in conjunction with other TFs, the nuclear NR2F1 motif was rare, indicating that NR2F1 is likely to bind indirectly to such pREs (*SI Appendix*, Fig. S2*J*).

Many pREs exhibited combinatorial TF binding. Our hypothesis was that regions bound by multiple TFs are important for regulating pallial regional patterning in the developing cortical VZ. Thus, we looked for loci which were bound by all five TFs. At the whole-genome level, these pREs were enriched at loci associated with transcriptional activity/DNA binding and genes with functions in forebrain and neuronal development (*SI Appendix*, Fig. S2*E*). These pREs were predominantly found at distal loci (*SI Appendix*, Fig. S2*H*). They were highly enriched around TF genes that are expressed in the embryonic pallium (blue bubbles in Fig. 4*E*) as well as non-TFs that are implicated in cortical development (e.g., *Bone Morphogenetic Proteins*, *Ephrins*, and *Semaphorins*; *SI Appendix*, Fig. S2*I*). Of particular note, pREs with combinatorial binding were enriched around TFs that we identified as being a part of the CRTFN (dark blue bubbles in Fig. 4*E*). Considering the full set of pREs, we found 1,236 unique TF binding sites associated with genes in the network (Dataset S2). Based on the proximity ligation assisted chromatin-immunoprecipitation (PLAC-seq)-defined interactome (see *Promoter–Enhancer Binding around 35 TFs of the CRTFN*), 33/38 CRTFN genomic loci had at least one pRE that was co-occupied by at least three to five TFs (Fig. 4*D*). For the majority of these loci (30/38, 79%), the enhancer was within 100 kb of the transcription start site (TSS). We tested the probability of this occurring by chance by randomly selecting 1,000 sets of 38 genes and evaluating the presence of three to five TFs occupancy in a distal enhancer within 100 kb of the TSS. Randomly sampled sets had a median of 7/38 (18%). Thus, the TSSs for the CFTRN network show enrichment (4.3-fold) for local combinatorial distal enhancers bound by the TFs examined here. We hypothesize that these pREs are likely to participate in regulating TF gene expression during patterning of the cortical VZ.

### pREs Bound by Multiple TFs Are More Likely to Be Active Enhancers.

To assess the activity of pREs defined by ChIP binding, we explored whether they are located in known enhancers that are active at E11.5 [VISTA enhancers—https://enhancer.lbl.gov (18)]. These enhancers were primarily ascertained by screens of evolutionarily conserved sequences or p300 ChIP-seq and thus represent an unbiased set with regard to the binding patterns of the TFs profiled here. We classified VISTA enhancers based on where they are active at E11.5: Pallial (*n* = 55), Subpallial (*n* = 56), and Nontelencephalic (*n* = 75). We also identified Inactive enhancers (*n* = 121; Dataset S3). We found that TF binding (one to five TFs) was present on 91% of Pallial, 73% of Subpallial, 51% of Nontelencephalic, and 31% of Inactive enhancers (*SI Appendix*, Fig. S5*C*). This indicates that nearly all genomic loci from this unbiased set of validated pallial enhancers exhibit binding of at least one of the CRTFN TFs.

Strikingly, 35% of the VISTA enhancers with Pallial activity were bound by four or five TFs. In comparison, only 23% of Subpallial, 1% of Nontelencephalic, and 6% of Inactive VISTA enhancers showed this pattern (*SI Appendix*, Fig. S5*C* and Dataset S3). Thus, binding by four to five TFs appears to be a potent indicator of enhancer activity. Furthermore, we estimate that there are 115 such loci associated with the CRTFN. Thus, CRTFN pREs bound by multiple TFs are likely to have pallial activity.

We hypothesized that TF binding would predict regional pRE activity. To test this, we leveraged the information provided by TF combinatorial binding and applied it in the context of cortical gradients and/or cortical subregions.

### Expression Gradients Are Regulated by the Combinatorial Binding of EMX2, LHX2, NR2F1, PAX6, and PBX1.

**Fig. 5.**
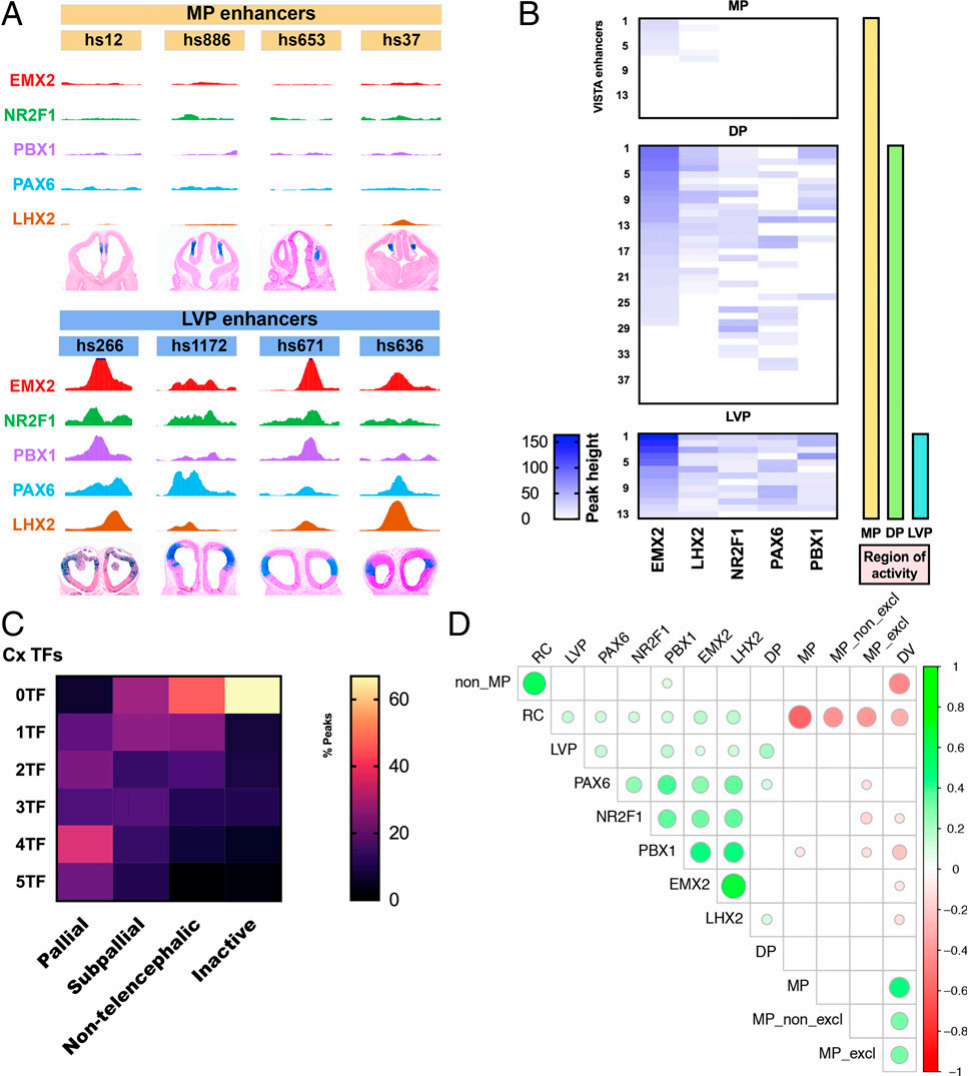
Combinatorial binding of EMX2, LHX2, NR2F1, PAX6, and PBX1 predicts gradients of enhancer activity in the pallium. (*A*) LVP enhancers (hs266, hs1172, hs671, and hs636) are enriched for ChIP-seq peaks but MP enhancers (hs12, hs886, hs653, and hs37) lack peaks. (*B*) Heatmap showing enrichment (peak height) of EMX2, LHX2, NR2F1, PAX6, and PBX1 binding over VISTA enhancers with MP exclusive activity (*n* = 16), MP + DP activity (*n* = 40), and MP + DP + LVP activity (*n* = 13) symbolized by the yellow, green, and blue vertical bars. Each row represents a distinct VISTA enhancer and the coverage of TF peaks at that locus. (*C*) Heatmap of combinatorial TF binding (percentage enrichment; 0 to 5 TFs) on VISTA enhancers that have pallial (*n* = 55), subpallial (*n* = 56), nontelencephalic (*n* = 75), and no activity (inactive; *n* = 121). (*D*) Correlalogram of computational modeling showing the different combinations of TF binding and their predictions of gradients of activity (*P* < 0.01). Green circles indicate correlation, and red circles indicate anticorrelation. The size of the circles is associated with the strength of the correlation/anti-correlation. For instance, RC enhancer activity is correlated with PAX6, NR2F1, PBX1, EMX2, and LHX2 TF ChIP-Seq.

We were interested in determining whether combinatorial binding of TFs was predictive of the activity pattern of pREs and/or of their cognate genes. We looked at VISTA enhancers that specifically had cortical expression patterns and classified their expression in cortical subregions: LVP, DP, and MP (as in Fig. 1*A* and Dataset S4). Next, we correlated the ChIP-seq peak heights, normalized for coverage depth differences across TFs, and found association between ChIP-seq signal strength and cortical regional activity (Fig. 5*B*). While ChIP-seq signal was present across a large proportion of regions, LVP active enhancers appeared to exhibit a particularly strong ChIP-seq signal (Fig. 5 *A* and *B*).

Enhancers with LVP activity had the most robust peaks (>25% percentile for peak enrichment, 100%, *n* = 13; Fig. 5 *A* and *B*). In contrast, peaks over enhancers whose activity was exclusively in the MP displayed no TF peaks in ∼56% of cases (*n* = 12) or small peaks (<25% percentile for peak enrichment) in ∼44% of cases (*n* = 6). Peaks that were over enhancers with DP activity (88% peaks, *n* = 25) or in mixed regions (DP + MP − 100% peaks, *n* = 14) displayed more heterogeneity in peak height but were often between both extremes observed in LVP and MP VISTA enhancers. TF binding and peak height enrichment for each individual VISTA enhancers that have regional activity is shown in Fig. 5*B*. Thus, quantitative enrichment from ChIP-seq suggests stronger TF–pRE interactions at LVP and DP pallial enhancers.

Some VISTA enhancers had spatial activity gradients along the pallial RC and DV axes. For instance, hs798 has both CR and DV gradients, whereas hs636 has RC and VD gradients (*SI Appendix*, Fig. S3*B*). To model our findings, we examined the correlation of TF binding, enhancer spatial activity gradients, and regionally defined enhancer activity. We found that peak presence was correlated with RC and LVP enhancer activity (Fig. 5D and *SI Appendix*, Fig. S3*C*). In contrast, the presence of EMX2, LHX2, NR2F1, and PBX1 was anticorrelated with MP and DV enhancers (*P* < 0.01 for all correlations). This implies that the binding of these five TFs promotes activity of enhancers with RC and/or LVP activity but not enhancers with MP activity. These results provide a gene regulatory logic where TF–RE interactions generate spatial gradients across the developing pallium.

### Profiling the Epigenomics of Cortical Progenitors.

**Fig. 6.**
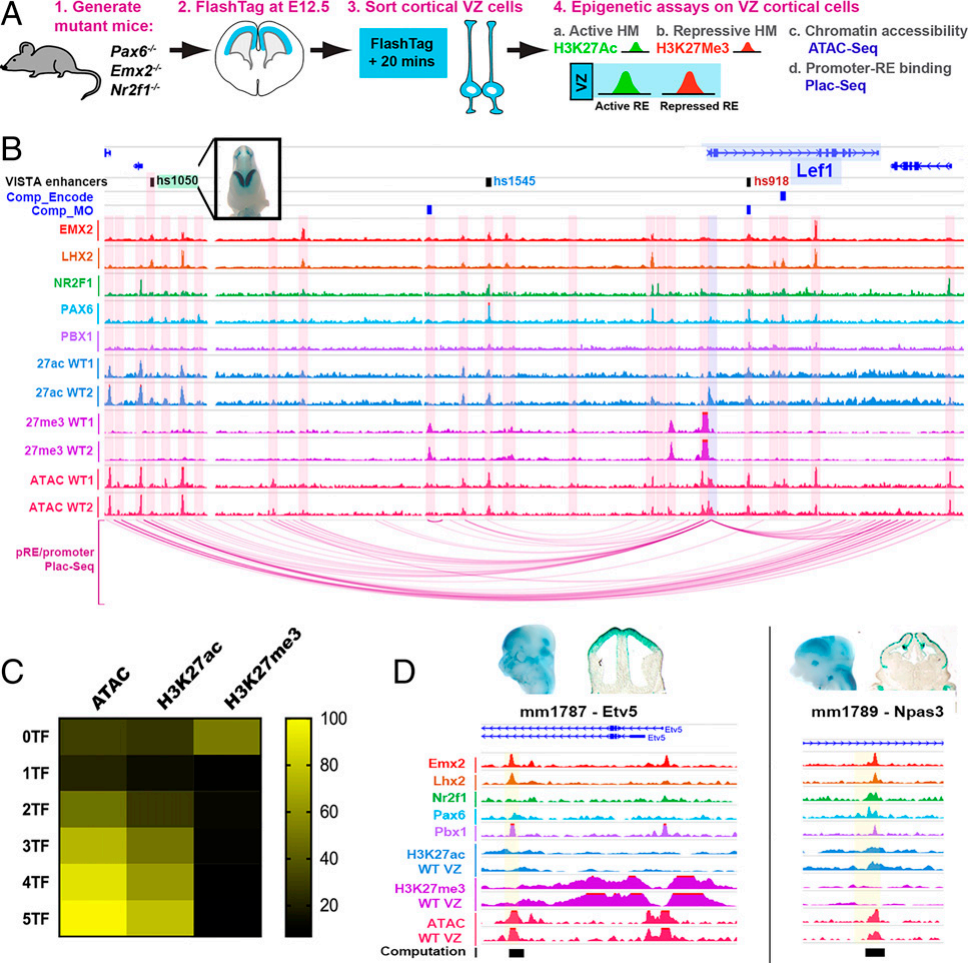
pRE landscape around CRTFN TF genes in cortical progenitors. (*A*) Method for VZ cell purification for epigenomic experiments (*Materials and Methods*). (*B*) Coverage of TF ChIPs, VZ histone marks (active: H3K27ac; repressive: H3K27me3), and VZ ATAC-seq in the *Lef1* locus at E12.5. pRE–promoter interactions are derived from PLAC-seq (purple arcs *Below*) and computationally (blue boxes *Above*, Comp_Encode and Comp_MO; Dataset S5). VISTA enhancers are also indicated (green = cortical activity, blue = noncortical activity, and red = inactive). (*C*) Heatmap of percentage enrichment of epigenomic marks over pREs with differential combinatorial TF binding (0 to 5 TFs). (*D*) Enrichment of TF and epigenomic marks over newly identified cortical enhancers near CRTFN genes: mm1787 (*Etv5*) and mm1789 (*Npas3*).

To link TF binding with pRE activity, we profiled chromatin state in WT cortical VZ cells isolated using the FlashTag method (34) (Fig. 6*A* and *SI Appendix*, Fig. S4 *A* and *B*). From these VZ cells, we made nuclei and performed a chromatin accessibility assay (ATAC-seq) and histone ChIP-seq with antibodies to histone modifications associated with active pRE state (H3K27ac) and repressed pRE state (H3K27me3) (35, 36). In CRTFN loci, we found 749 ATAC, 556 H3K27ac, and 564 H3K27me3 peaks. Examples of VZ epigenomic peaks are illustrated in the *Lef1* locus, with pREs highlighted in pink (Fig. 6*B*).

In general, there was a high degree of overlap of TF binding, H3K27ac, and ATAC peaks; H3K27me3 peaks were less likely to be centered on TF, ATAC, and H3K27ac peaks. The proportion of overlap with TF binding (one to five TFs) was 40% for ATAC peaks, 26% for H3K27ac, and 10% for H3K27me3 (Fig. 6*C*). Interestingly, as TF binding increased from zero to five TFs, the likelihood of a pRE having an ATAC or H3K27ac peak increased proportionally (Fig. 6*C*). All pREs where five TFs were bound had ATAC peaks, and ∼78% had H3K27ac (active) marks. An inverse relationship was found between H3K27me3 marks and TF binding; only ∼10% of five TF-bound pREs had a H3K27me3 mark. On the other hand, 60% pREs with no TF peaks had H3K27me3 binding. Complete genomic tracks for each of the 38 TF loci are shown in *SI Appendix*, Fig. S6.

Overall, active marks and repressive marks were evenly represented across the 2,083 pREs identified in the CRTFN (27% for each histone modification). At individual genomic loci though, there was a range of acetylated and repressed pREs (Fig. 6*D* and *SI Appendix*, Fig. S4*C*). Certain loci were enriched for H3K27me3 (i.e., *Emx2*, *Dmrt3*, and Sp8), whereas others were highly enriched for H3K27ac (i.e., *NfiB*, *Bcl11a*, and *Lhx2*) (Fig. 6*D* and *SI Appendix*, Figs. S4*C* and S5*B*). In general, genes with MP expression had pREs that were significantly more repressed than pREs in other genes (acetylation/methylation ratio is 0.38 versus 2.80, *P* < 0.05, unpaired two-tailed Student’s *t* test; *SI Appendix*, Figs. S5*B* and S4*C*). Thus, the analysis of histone modifications in the CRTFN on purified VZ cells has provided evidence for which REs are active and repressed during cortical VZ patterning.

### Promoter–Enhancer Binding around 35 TFs of the CRTFN.

To define promoter–enhancer units, and thereby gain information about the looping structure of the chromatin, we performed PLAC-seq on E12.5 cortices (37). We dissociated ∼6 million cortical cells, immunoprecipitated promoters using a known promoter epigenomic mark (H3K4me3), and then identified chromosomal interactions for those promoters (38). The identification of enhancer–promoter units that surpassed a false discovery rate (FDR) < 0.01 yielded a total of 39,655 interactions using 5-kb bin pairs and 118,701 interactions with 10-kb bin pairs. Over 90% of these interactions were located at distal loci (at least 2,500 base pairs away from the TSS of a gene).

Enhancer–promoter loops show points of contact between TF gene promoters and multiple pREs (see *Lef1* locus, Fig. 6*B* and *SI Appendix*, Fig. S6). PLAC-seq loops terminated at regions of chromatin accessibility, TF binding, and H3K27Ac, as well as at VISTA enhancers with cortical activity (i.e., hs1050) (Fig. 6*B*). The PLAC-seq data allowed us to assign some of the 2,083 CRTFN pREs to specific genes based on interaction between the pREs and the promoter region of genes. Within the CRTFN, we identified 619 pRE–gene interactions (using 5-kb bin pairs) and 945 pRE–gene interactions (using 10-kb bin pairs, Dataset S5). In addition, we complemented the PLAC-seq results with previously computationally derived enhancer–gene associations based on correlation between the putative enhancer activity and the expression level of the genes residing in the same topologically associating domain (Dataset S5) (39, 40). Thus, we defined the interactome of cis-REs around the TSS for each CRTFN genomic locus.

### Epigenomic Marks Are Good Predictors of pRE Activity.

We tested whether chromatin state was predictive of cortical progenitor activity for pREs by examining open chromatin and histone marks on VISTA enhancers in Dataset S3. As with TF binding, Pallial VISTA enhancers were more likely to have ATAC and H3K27ac peaks than Subpallial, Nontelencephalic, or Inactive enhancers (*SI Appendix*, Fig. S5*C*). In contrast, H3K27me3 was not enriched on cortical VISTA enhancers compared to other enhancers (active in other tissues or inactive).

Next, we tested the enhancer activity of additional pREs in the CRTFN by selecting a set of loci with three to five TF binding and ATAC epigenomic marks. We tested 18 pREs using a transgenic reporter assay at E12.5 (18). We found that 15/18 pREs had positive enhancer activity at E12.5 with at least three embryos showing the same pattern of activity (Dataset S6). Of those, 5/15 had activity in the cortex, and an additional 8/15 had activity in surrounding regions such as the pretectum or subpallium (Dataset S6). Two of these enhancers are shown in Fig. 6*E* and displayed activity in the developing DP (mm1787 near *Etv5*) as well as the CDP and MP (mm1789 near *Npas3*) (Fig. 6*E*). Thus, integrating TF and histone ChIP with ATAC data provides an efficient approach to identify REs that are active in cortical progenitors.

### Identification of pREs That Are Sensitive to Changes in Cortical Patterning in *Emx2^−/−^, Nr2f1^−/−^*, and *Pax6^−/−^*.

Having identified 2,083 CRTFN pREs in cortical progenitor cells, we next sought evidence for which of these may participate in regulating regional patterning. To this end, we looked for changes in pRE chromatin state in *Emx2^−/−^*, *Nr2f1^−/−^*, and *Pax6^−/−^*, an approach we previously applied in *Nkx2-1* and *Dlx1/2* mutants (35, 41). We used the Flash-Tag/fluorescence-activated cell sorting (FACS) method to purify cortical VZ progenitors from E12.5 WT and mutant littermates. After FACS sorting, we conducted ATAC-seq and histone ChIP-seq (H3K27ac and H3K27me3). We annotated the data as the following: Gain (gain of enrichment in a peak in the mutant), Loss, or No change in the CRTFN; we found 204 pREs (12% of pREs) that were affected in the mutant cortex (“differential pREs”) (Dataset S7).

**Fig. 7.**
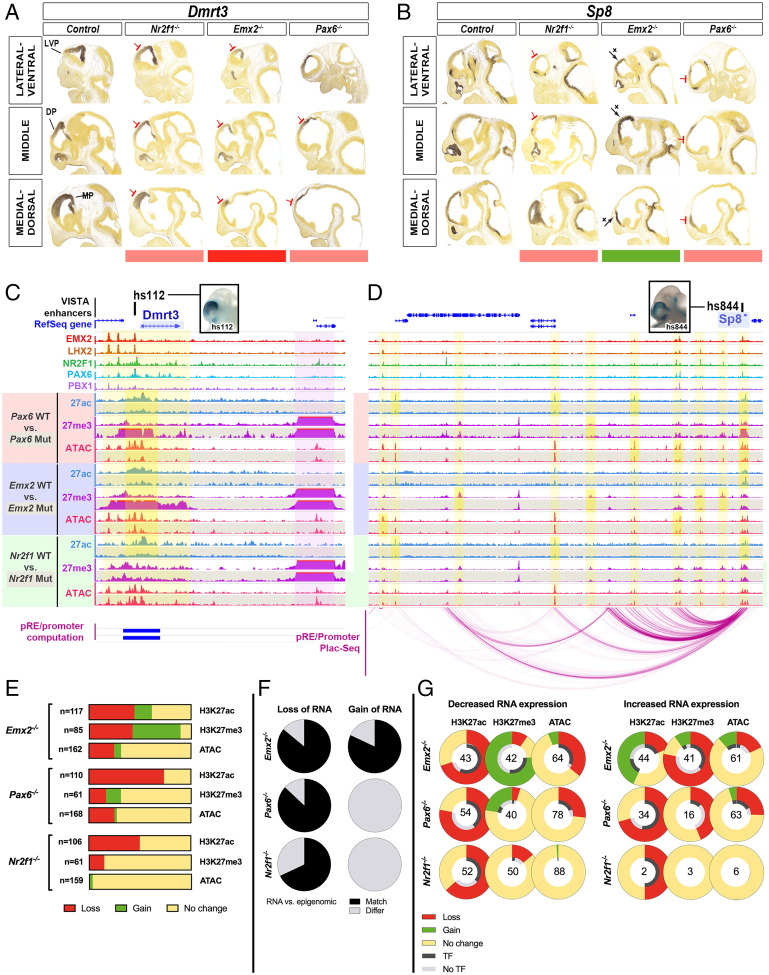
pREs show epigenetic changes in *Emx2^−/−^*, *Nr2f1^−/−^*, and *Pax6^−/−^* (*A* and *B*) ISH for *Dmrt3* and *Sp8* in WT, *Nr2f1^−/−^*, *Emx2^−/−^*, and *Pax6^−/−^* at E11.5. Red and green bars below indicate decrease and increase in expression. (*C* and *D*) TF ChIPs and VZ epigenomic marks (H3K27ac, H3K27me3, ATAC-seq) at *Dmrt3* and *Sp8* loci at E12.5 in WT, *Pax6^−/−^* (red), *Emx2^−/−^* (blue), and *Nr2f1^−/−^* (green). WT tracks (shaded white) are *Above* mutant tracks (shaded gray). pRE–promoter interactions are derived from PLAC-seq (purple arcs *Below Sp8* locus) and computationally (blue boxes *Below Dmrt3* locus). Two cortical VISTA enhancers (hs112 and hs844) are shown *Above* the genomic loci (black squares); pictures of embryo wholemounts show their cortical activity (blue stain). Yellow vertical bars highlight regions of differential epigenomic marks in mutants, and a purple vertical bar highlights *Dmrt2,* a region next to *Dmrt3* that is unchanged. (*E*) CRTFN pREs with differential enrichment of epigenomic marks in the *Emx2^−/−^*, *Pax6^−/−^*, and *Nr2f1^−/−^* were recorded. Loss (red), Gain (green), and No change (yellow) were recorded for individual pREs and tabulated across all peaks for individual epigenomic marks. (*F*) Proportion of loci showing a match (black) or a mismatch (gray) between the RNA ISH analysis and the differential H3K27ac/H3K27me3 ratio in the TF locus, in *Emx2^−/−^*, *Pax6^−/−^*, and *Nr2f1^−/−^*. For *Nr2f1^−/−^*, there is not enough information to make comparisons (only one TF that shows a gain in RNA expression; that locus has two pREs). (*G*) pREs in TFs loci were classified as having either a decrease, an increase, or no change in RNA expression in the *Emx2^−/−^*, *Pax6^−/−^*, and *Nr2f1^−/−^*. We show the changes in epigenomic marks in the TF genes’ interactome according to whether the TF gene showed increased or decreased RNA expression. The percent change compared to WT littermates is plotted in the outer ring (Loss in red; No change in yellow; Gain in green). For pREs that changed, we assessed whether we found a TF peak at that pRE (i.e., in the case of *Pax6^−/−^*, we looked for a PAX6 ChIP peak). The proportion of differential pREs with or without the cognate TF binding ChIP-seq reads is presented in the inner ring (black for presence of the TF binding peak; gray for the absence of the TF binding peak). Numbers of differential pREs assessed for each TF gene locus are shown in the center of the rings.

Of these 204 differential pREs, we could associate over 80% with a TF gene promoter either by PLAC-seq or by computational analysis (Dataset S7). The annotated changes in epigenetic state of differential pREs for each mutant are presented in Fig. 7*G*. *Pax6*^−/−^ showed a 69% loss of H3K27ac marks (81/118) and no increases and a balanced loss and gain of H3K27me3 (12 and 11%, respectively). Consistent with histone changes, loss of chromatin accessibility (25%) occurred more frequently than an increase in chromatin accessibility (2%). *Emx2*^−/−^ showed a 44% loss and a 17% gain of H3K27ac and a 42% loss and 47% gain of H3K27me3. Chromatin accessibility was more often lost (25%) than gained (7%). Finally, *Nr2f1*^−/−^ showed a 45% loss of H3K27ac and an 11% loss of H3K27me3 and no gains in either mark. Chromatin accessibility only showed a change in 3% of peaks (Fig. 7*E*).

We found that enhancers that lose H3K27ac in *Emx2^−/−^* are bound by LHX2 in 68% of cases; compared to 24 and 35% when H3K27Ac is gained or unchanged in *Emx2^−/−^*, respectively. In addition, we used Homer (29) to perform motif analysis on enhancers that were repressed (loss of H3K27Ac and/or gain of H3K27me3) or activated (gain of H3K27Ac and/or loss of H3K27me3) in *Emx2^−/−^*. We found that the LHX2 motif was significantly enriched (compared to background) in enhancers that lost H3K27Ac (*P* = 1 × 10^5^) and enhancers that gained H3K27me3 (*P* = 1 × 10^2^) in *Emx2^−/−^*. Conversely, the LHX2 binding motif was not detected in enhancers that became more active in *Emx2^−/−^*. Taken together, this suggests that enhancers that have LHX2 binding are more likely to be turned off than those that do not have LHX2 binding in *Emx2^−/−^*.

We next checked whether these epigenomic changes were consistent with changes in RNA expression within the CRTFN (Fig. 7*F*). In genes exhibiting a decrease in RNA expression, we expect a loss of H3K27ac and a gain of H3K27me3 (42). In *Emx2^−/−^*, 14 TFs had reduced RNA expression, and 86% of these genomic loci exhibited an overall loss of active and/or gain of repressive marks on pREs (Dataset S7). In *Pax6^−/−^*, 15 TF genes had a reduction in RNA expression, and 87% of these genomic loci showed an overall loss of active and/or gain of repressive marks. Finally in *Nr2f1^−/−^*, 19 TF genes had a reduction in RNA expression, and 13 (68%) of these genomic loci showed an overall loss of active marks at pREs. This indicates that for a given TF gene, reduction of RNA expression in a mutant is correlated with a reduction of the H3K27ac/H3K27me3 ratio in the pREs of that locus.

In contrast, for genes showing an increase in RNA expression, we expect a loss of H3K27me3 and/or a gain of H3K27ac (increased H3K27ac/H3K27me3 ratio). *Emx2^−/−^* met this expectation; 9/11 TF genes that had increased RNA expression also had pREs that lost H3K27me3 and/or gained H3K27ac. On the other hand in *Pax6^−/−^*, increased RNA expression was not associated with an increased H3K27ac/H3K27me3 ratio.

The summary of changes in epigenomic profiles for pREs based on changes in ISH RNA expression in *Emx2^−/−^*, *Nr2f1^−/−^*, and *Pax6^−/−^* are presented in Fig. 7*G*. The schema is organized according to Loss, Gain, and No change in RNA expression in the three mutants. The outer ring depicts percent changes for each epigenomic mark in pREs in the mutants (Loss, No change, and Gain). The inner ring depicts the presence (Black) or absence (Gray) of the cognate TF ChIP binding peak (i.e., PAX6 in *Pax6^−/−^*). The number in the center of the rings represents the number of pREs investigated. Overall, we suggest that loss of H3K27ac is associated with reduced RNA expression and with TF protein binding to pREs in the *Emx2^−/−^*, *Nr2f1*^−/−^, and *Pax6^−/−^*.

Next, we show two gene loci (*Dmrt3* and *Sp8*) in Fig. 7 *C* and *D* with the cognate change in RNA expression presented in Fig. 7 *A* and *B* (see *SI Appendix*, Fig. S6 for all 38 TF loci in the CRTFN). In the genomic loci of these 2 TFs, we highlighted pREs that showed epigenomic changes in yellow. *Dmrt3 RNA* expression is down-regulated in all mutants. At the *Dmrt3* locus, hs112 showed a decreased H3K27ac/H3K27me3 ratio and a loss of chromatin accessibility in both *Emx2^−/−^* and *Pax6^−/−^*. *Sp8* is down-regulated in *Pax6*^−/−^ but increased in *Emx*2^−/−^. pRE epigenetic changes were in synchrony with this. For instance, the H3K27ac/H3K27me3 ratio on hs844 was reduced in *Pax6^−/−^* and increased in *Emx2^−/−^*. Other pREs around the *Sp8* locus showed epigenetic changes in only one or two mutants and were unchanged in the other knockouts.

We identified 20 to 120 pREs per locus, with 10% showing epigenetic changes in the TF mutants (*SI Appendix*, Fig. S7). Understanding how individual pREs regulate transcription at a single gene locus will require investigating these pREs individually and in combination across normal and abnormal development.

## Discussion

We have identified the key elements (TFs and target genomic REs) in a transcriptional network that regulates early steps in cortical regional patterning in mice. This CRTFN is composed of TFs and their pREs. We tested the validity of this network by characterizing the impact of genetic ablation of three key members (*Emx2*, *Nr2f1*, and *Pax6*) on the expression of TF genes and the epigenomic state of pREs. Within the CRTFN, 30/31 of the TF genes were sensitive to genetic ablation of at least one of these three TFs. pREs were identified using TF ChIP-seq for EMX2, LHX2, NR2F1, PAX6, and PBX1, and epigenomic assays for chromatin accessibility, active pREs (H3K27ac), and repressive pREs (H3K27me3). pREs were further assessed by PLAC-seq–defined promoter–enhancer looping or computationally derived enhancer–gene associations. We found that pREs with combinatorial binding of different TFs were enriched at enhancers displaying cortical activity (VISTA enhancers) and that their binding was correlated with activity in the LVP and RC gradients and anticorrelated with activity in the MP and DV gradients. We also showed that five CRTFN pREs had cortical activity in transient transgenic assays. Approximately10% pREs were sensitive to loss of EMX2, NR2F1 or PAX6; these showed a change in the H3K27ac/H3K27me3 ratio. Taken together, our integrated approach has identified a TF network involved in regional patterning of the cortex.

### Defining a CRTFN.

We carried out an unbiased screen to discover TFs that have informative patterns in the E11.5 pallium using the Developing Mouse Brain Atlas (developingmouse.brain-map.org). TFs at E11.5 were expressed either in gradients (∼70) or homogeneously across cortical regions (∼150). We did not find TFs with sharp intrapallial boundaries, except for six whose expression was restricted to the MP. This suggests that cortical areas do not originate from progenitor domains with spatially restricted TF expression but rather from a process that integrates information from TF gradients to create sharp boundaries.

We focused our analysis on testing the sensitivity of 31 CRTFN TFs with graded expression patterns in *Emx2^−/−^*, *Nr2f1^−/−^*, and *Pax6^−/−^*. CR TFs showed decreased expression in *Emx2^−/−^* and *Nr2f1^−/−^*, whereas RC TFs showed decreased expression in *Nr2f1^−/−^* and *Pax6^−/−^.* Patterning TFs often act in concert to regulate regional patterning of pallial subregions (43). Here, we present evidence that *Nr2f1* and *Pax6* act together to coregulate the development of the rostroventral cortex. We found that *Pax6* promotes *Nr2f1/2* expression and that, together, these TFs coregulate other rostroventral TFs including *Lmo3* and *Npas3*, which in turn plays a role in patterning the rostral piriform cortex and adjacent structures. Of note, the rostral subplate is also reduced in the *Pax6^−/−^*, *Nr2f1/2 cKO*, and *Npas3*^−/−^ (Fig. 3), suggesting that its development is linked to the rostroventral cortex (44).

Thus, we defined CRTFN TFs that are likely to play a role in cortical regional patterning. Further, it enabled us to identify a *Pax6-Nr2f1-Npas3-Lmo3* pathway that regulates patterning of the rostroventral cortex.

### The Epigenome of CRTFN pREs Predicts Their Spatial Gradients of Cortical Activity.

Having defined the key TFs playing a role in cortical patterning, we aimed to uncover the regulatory network governing the expression of TF gene gradients. To this end, we identified pREs of the CRTFN.

Combinatorial binding of EMX2, LHX2, NR2F1, PAX6, and PBX1 was a strong predictor of pRE activity in the developing cortex (Fig. 5*C*). Biochemical and computational chromatin conformation analysis provided evidence that at least 45% of pREs interacted with a given TF gene’s promoter and defined the cis-regulatory interactome for each CRTFN gene. In total, 91% of cortical Vista enhancers in the CRTFN had binding of at least one TF. Moreover, combinatorial binding of TFs was strongly enriched in Pallial Vista enhancers compared to Subpallial, Nontelencephalic, and Inactive enhancers.

Here, we uncovered some of the transcriptional logic underlying differences in enhancer regional activity using two different methods: 1) degree of TF binding (EMX2, LHX2, NR2F1, PAX6, and PBX1) on VISTA enhancers with regional activity and 2) an unbiased computational approach (Fig. 5). We found that enhancers active in the most dorsal caudal pallium (MP) had the least binding, whereas enhancers active in the most rostral ventral pallium (LVP) had the most binding. This bias is not due to expression of the TFs (*Emx2*, *Lhx2*, *Nr2f1*, and *Pax6* are expressed in the MP), nor is it entirely due to a bias in cell numbers since LVP and MP are both small structures in comparison to the DP, which had a level of TF binding intermediate to that of the LVP and MP. We propose that there is a distinct set of MP TFs, such as those found in our screen, *Id3* and *Lmx1a*, that promote the activity of MP enhancers (Fig. 1) (45). Thus, we have shown that the combination of EMX2, LHX2, NR2F1, PAX6, and PBX1 binding in pREs provided a signature that was predictive of pRE regional activity and cortical gradients.

Toward establishing an epigenetic metric for a gene’s activity, we calculated the H3K27Ac/H3K27me3 ratio within the interactomes of each CRTFN TF. We found that pREs within the genetic locus of TFs with expression in the MP had a lower H3K27Ac/H3K27me3 ratio than that of non-MP TF gene loci (*SI Appendix*, Fig. S5). We suggest that pREs of MP genes are repressed in the DP and LVP, and we predict that a focused analysis on the VZ of the MP would find a H3K27Ac/H3K27me3 > 1.

To gain insights into the function of the pREs, we assessed their epigenomic state. We restricted our study to VZ cells to bypass issues relating to cellular heterogeneity. We found that VZ ATAC and H3K27ac peaks were proportionally enriched at pREs that have increased cobinding by TFs (*SI Appendix*, Fig. S5*C*). This indicates that pREs with combinatorial TF binding are accessible in the VZ and are likely to be active. We estimated that CRTFN gene loci have between 20 and 100 pREs, thus underscoring the complexity of regulatory landscapes (Dataset S2).

This analysis identified ∼2,000 pREs in the CRTFN based upon their chromatin state and binding by TFs important for cortical patterning. Cobinding of multiple TFs, as well as the presence of ATAC and H3K27ac marks, is correlated with enhancer activity. Moreover, our results suggest that binding of TFs can predict enhancer spatial activity gradients, thus uncovering a transcriptional logic to cortical regional patterning.

### pREs of the CRTFN Respond to Loss of EMX2, NR2F1, or PAX6 through Changes of H3K27ac/H3K27me3 Ratios Rather than Changes of Chromatin Accessibility.

At the gene locus level, transcriptional regulation requires the integration of RE function. In the CRTFN, each TF gene can have 20 to 100 pREs. Our analysis uncovered complexity in the properties and regulation of individual pREs within the CRTFN when we probed their epigenomic profiles in *Emx2^−/−^*, *Nr2f1^−/−^*, and *Pax6^−/−^*.

Enhancers are spatial and temporal integrators of combinatorial TF binding and chromatin state (46). While the majority of pREs did not exhibit epigenetic changes in the three mutants, we did find 204 pREs that were differentially regulated, of which >80% were linked to the TSS of the TF gene (Dataset S7). This suggests that these pREs are responsive to ablation of these specific TFs and likely important for regional patterning of the cortical neuroepithelium.

Loss of EMX2, NR2F1, and PAX6 had different effects on distinct pREs. In some cases, changes in the epigenetic state of a pRE was similar in two or three mutants (38%). For instance, *hs112* in *Dmrt3* showed loss of H3K27ac in the three mutants, gain of H3K27me3 in *Pax6^−/−^ and Emx2^−/−^*, and loss of chromatin accessibility in *Pax6^−/−^* (Fig. 7). Other pREs (∼9%) responded differently in different mutants. For instance, *hs844* in the *Sp8* locus showed a reduced H3K27ac/H3K27me3 ratio in the *Pax6^−/−^* but an increased ratio in the *Emx2^−/−^*. In 52% of occurrences, pREs showed changes in only one TF mutant, reflecting the specificity of pRE regulation. While histone modifications were sensitive marks of pRE epigenetic changes, the ATAC-seq assay of chromatin accessibility showed few changes in all three mutants (Fig. 7*G*). Thus, while ATAC-seq was effective at identifying active pREs (Fig. 6*C*), it was not an effective tool to assess which were modified in the VZ of *Emx2^−/−^*, *Nr2f1^−/−^*, and *Pax6^−/−^*.

At the gene locus level, each TF gene had between 1 and 13 differentially regulated pREs in the three mutants. In 93% of cases, all differentially regulated pREs in the same locus showed a similar change in their H3K27ac/H3K27me3 ratio in a given mutant, such as at the *Dmrt3* locus in *Pax6^−/−^* and *Emx2^−/−^*. Perhaps, some of these pREs can compensate for one another making their regulatory programs more robust to mutations, such as in the *Arx* loci (47). However as for *Arx* enhancers, such redundant enhancers could also have distinct functions.

Integration of discrete RE function across a locus leads to overall transcriptional patterns of the target genes. We found that genes showing reduced cortical RNA had coherent changes in epigenomic marks in the pREs of mutants (Fig. 7 *H* and *I*). Likewise, genes showing increased RNA had coherent changes in epigenomic marks in the *Emx2^−/−^* mouse but surprisingly not in the *Pax6^−/−^*. Understanding why *Pax6^−/−^* showed increased RNA may require probing different epigenomic marks and/or investigating indirect mechanisms of transcriptional activation. The most pronounced epigenomic change that we detected was the loss of H3K27ac marks in *Pax6^−^*^/-^ (Fig. 7*G*). PAX6 binds several chromatin modifiers, including p300, a transcriptional coactivator and histone acetyltransferase (48); perhaps this accounts for the reduction in the H3K27ac marks in *Pax6^−^*^/-^. Thus, further integration of TFs and chromatin regulators will be crucial in understanding the epigenetic code that regulates cortical development.

Around 10% of CRTFN pREs were epigenetically changed in *Emx2^−/−^*, *Nr2f1^−/−^*, and *Pax6^−/−^*. These were enriched for promoter looping. We propose that these pREs are the core set of enhancers regulating cortical patterning. Combined with studies on the transcriptomic and epigenomic landscape in the developing human cortex (36, 49–52), our analyses will set the stage for understanding VZ regionalization in the developing human cortex. It is important to note that although the TFs we studied are conserved in humans, the pREs that we identified here in mice might not be conserved in the developing human cortex.

### Propagating the VZ Protomap into Regionalization of the Cortical Laminae.

Here, we define TFs within the CRTFN that exhibit regional gradients in the developing mouse cortical neuroepithelium. We demonstrate via genomic interaction mapping and genetic knockout that expression patterns of these TFs are regulated, at least in part, via the interaction of specific TFs with target REs. Considering the lack of TFs that are restricted to specific cortical VZ regions, pREs must encode regional activity via integrating the effects of combinatorial TF binding. Based on this, we hypothesize that the VZ enhancers identified here may serve as a protomap of cortical regions by integrating TF signaling. We previously postulated that enhancers serve as protein-binding modules that translate gradients of TFs in cortical progenitors into region-specific expression in cortical neurons (19, 53). Our hypothesis is that information encoded in the VZ by gradients of TFs will be encoded in the REs of genes important in cell specification. Along these lines, *Pax6* was shown to control regionalization through a downstream cascade beginning with the induction of *Tbr2* in the intermediate progenitors and propagated by *Tbr1* to the cortical plate (54, 55). Likewise, *Pbx1*, *Nr2f1*, and *Lhx2* function in immature cortical neurons to control cortical regionalization (6, 56). Moreover, at early postnatal stages, refinement of the somatosensory cortical neurons (coexpressing *Ctip2* and *Satb2*) was regulated by the modulation of epigenetic mechanisms in a time and area-specific manner by *Lmo4* (56). Here, we restricted our focus to studying the transcriptional control of patterning in the VZ. Future work will further elucidate the TF network (TFs and cis-REs) that plays a role in the propagation of the VZ protomap to the overlying cortical regions.

## Materials and Methods

All procedures and animal care were approved and performed in accordance with NIH and the University of California San Francisco Laboratory Animal Research Center guidelines. Cortical expression analysis was performed using the Allen Brain Institute Developing Mouse Brain database. Histology, transgenic assays, TF ChIP-seq, epigenomic, and PLAC-seq assays were performed and analyzed according to published protocols. More detail regarding specific experimental procedures and analyses can be found in *SI Appendix*.

## Supplementary Material

Supplementary File

Supplementary File

Supplementary File

Supplementary File

Supplementary File

Supplementary File

Supplementary File

Supplementary File

## Data Availability

ChIP data have been deposited in Gene Expression Omnibus database (GSE183130) (57).

## Change History

December 21, 2021: The author line has been updated.
