# Supplementary material for: Transcriptional network orchestrating regional patterning of cortical progenitors
Source: Proc Natl Acad Sci U S A. 2021 Dec 17;118(51):e2024795118. doi: 10.1073/pnas.2024795118 (PMC8713794; doi:10.1073/pnas.2024795118)
Supplement: Supplementary File [file pnas.2024795118.sapp.pdf]

PNAS
www.pnas.org

Supplementary Information for

Transcriptional Network Orchestrating Regional Patterning of Cortical Progenitors

Athéna R Ypsilanti[1], Kartik Pattabiraman[1], Rinaldo Catta-Preta[6], Olga Golonzhka[1], Susan Lindtner[1], Ke Tang[7], Ian R Jones[3], Armen Abnousi[2], Ivan Juric[2], Ming Hu[2], Yin Shen[3], Diane E Dickel[8], Axel Visel[8,9,10], Len A Pennacchio[8,9,11], Michael Hawrylycz[4], Carol L Thompson[4], Hongkui Zeng[4], Iros Barozzi[5], Alex S Nord[6], John L Rubenstein[1].

[1] Nina Ireland Laboratory of Developmental Neurobiology, Department of Psychiatry, UCSF Weill Institute for Neurosciences, University of California San Francisco, San Francisco, CA 94158 USA
[2] Department of Quantitative Health Sciences, Lerner Research Institute, Cleveland Clinic Foundation, Cleveland, OH 44195, USA
[3] Institute for Human Genetics and Department of Neurology, University of California, San Francisco, San Francisco, CA, USA.
[4] Allen Institute for Brain Science, Seattle, WA 98109, USA.
[5] Faculty of Medicine, Department of Surgery and Cancer, Imperial College, London, UK
[6] Department of Neurobiology, Physiology and Behavior, and Department of Psychiatry and Behavioral Sciences, University of California, Davis, Davis, CA 95618, USA
[7] Precise Genome Engineering Center, School of Life Sciences, Guangzhou University, Guangzhou, Guangdong 510006, China
[8] Environmental Genomics and Systems Biology Division, Lawrence Berkeley National Laboratory, Berkeley, CA 94720, USA
[9] U.S. Department of Energy Joint Genome Institute, Berkeley, CA 94720, USA
[10] School of Natural Sciences, University of California, Merced, CA 95343, USA
[11] Comparative Biochemistry Program, University of California, Berkeley, CA 94720, USA

* John L Rubenstein.
Email: John.Rubenstein@ucsf.edu

This PDF file includes:
Supplementary text
Figures S1 to S7
Tables S1
Legends for Datasets S1 to S7
SI References

SUPPLEMENTARY TEXT

MATERIALS AND METHODS

Mice and genotyping

All procedures and animal care were approved and performed in accordance with National Institutes of Health and the University of California San Francisco Laboratoy Animal Research Center (LARC) guidelines. Mice strains that were used have been previously reported: *Pax6*$^{Sey/Sey}$ (1), *Emx2*$^{-/-}$ (2), *NR2F1*$^{-/-}$ (3), *Emx1::Cre; NR2F1*$^{-/-}$*; NR2F2*$^{fl/fl}$ (4, 5) and *Npas3*$^{-/-}$ (6). All mice were genotyped as previously reported.

Method Details

Cortical expression analysis

The Developing Mouse Brain database of the Allen Brain Institute has generated *in situ hybridization* catalogs for hundreds of proteins in the mouse embryonic and postnatal brain. Three separate investigators annotated expression patterns of the TFs in the cortex at E11.5 by annotating both the density and the intensity of the *ISH* staining in the ventricular zone and the subventricular zone/mantle zone of the lateral ventral pallium (LVP), rostral dorsal pallium (RDP), caudal dorsal pallium (CDP) and medium pallium (MP). These annotations were then computationally mined to identify novel gradient patterns and/or regional expression.

Histology

Brains were fixed, cryopreserved and embedded as previously described (7). After cryostat-sectioning, brain sections were stained as described (8). In situ hybridization was performed as previously described (7). For details on how probes were generated, see ABA website.

Emx2 antibody production

A guinea-pig Emx2 antibody was generated by Genscript. It was raised against a peptide of the N-terminus of mouse Emx2 (aa 1-155) which specifically excluded the homeodomain of Emx2.

TF Chromatin Immunoprecipitation (ChIP)

Dissected cortices from embryos from multiple litters were dissociated and crosslinked at room temperature for 10 min in 1% formaldehyde (EMX2, LHX2) or 20 minutes in 1.5% formaldehyde (NR2F1, PAX6, PBX1) before being quenched for 5 mins in glycine (2.5mM) and washed gently with 1xPBS. Nuclei were extracted by lysing the fixed cells in a hypotonic buffer (50 mM Tris pH 7.5 / 0.5% NP40 / 0.25% Sodium Deoxycholate / 0.1% SDS / 150 mM NaCl). The crosslinked chromatin was sheared in dilution buffer (0.01% SDS, 1.1% Triton X- 100, 1.2mM EDTA, 16.7mM Tris-HCl, pH 8.1, 167mM NaCl) using a bioruptor (Diagenode) for either 18 rounds (for samples fixed in 1% PFA) or 40 rounds (1.5% PFA) (1 round = 30 s on/45 min off at high intensity) and incubated at 4°C overnight with 5 mg of antibody or either 20× blocking peptide or a control IgG which were used as negative controls as appropriate. Protein/antibody complexes were collected using Dynabeads (20 µL protein A + 20 µL protein G) before being washed in three wash buffers: low salt wash buffer (0.1% SDS, 1% Triton X-100, 2mM EDTA, 20mM Tris-HCl, pH 8.1, 150mM NaCl), high salt wash buffer (0.1% SDS, 1% Triton X-100, 2mM EDTA, 20mM Tris-HCl, pH 8.1, 500mM NaCl); and LiCl wash buffer (0.25M LiCl, 1% IGEPAL CA630, 1% deoxycholic acid (sodium salt), 1mM EDTA, 10mM Tris, pH 8.1and TE). Chromatin was eluted in Elution buffer (1%SDS, 10mM Codium bicarbonate buffer) at 65^{0}C for 10 min. Eluted chromatin was reverse crosslinked overnight at 65^{0}C with NaCl (500mM), then subsequently treated with RNase (10 µg/ 200 µl reaction, 15 min at 37˙C) and Proteinase K (10 µg/ 200 µl reaction, 60 min at 55˙C). DNA was then cleaned using a ChIP DNA Clean & Concentrator kit (Zymo Research). ChIP experiments were then validated by ChIP-qPCR using a 7900HT Fast Real-Time PCR System (Applied Biosystems) with SYBR Green qPCR SuperMix (Invitrogen, Cat. No. 11760-100).

ChIP-seq libraries were prepared using the Ovation Ultralow DR Multiplex System (NuGEN). Generated libraries were size selected using Blue Pippin (centered around 300 bp), QC tested on a Bioanalyzer

(Agilent) and sequenced as single-end 50 nucleotide reads on a HiSeq 4000 (Illumina) at the Center for Advanced Technology at UCSF (http://cat.ucsf.edu/).

ChIP-seq Computational Analysis

Clustering, base calling, and quality metrics were performed using standard Illumina software. Sequencing libraries were analyzed for overall quality and were filtered, and reads were mapped to the mouse genome (mm9) using Burrows-Wheeler Alignment (BWA) (9).

Pairwise Pearson Correlation

Pearson correlation between aligned read counts of pairs of TFs was determined using DeepTools (10) to show association between TFs and between different TF replicates.

Motif analysis

De novo motif discovery and enrichment was performed using HOMER version 4.9 (11) in the called peaks for each individual TF, using standard parameters, 300 bp up- and downstream of TF peaks. We compared the significant motifs discovered with the JASPAR (25) database. Motif enrichment was determined for all motifs present in the HOMER known motif database with p-value < 10^{-100}. We established the average motif coverage enrichment plot around 300 bp of peaks for each TF using custom R scripts.

Gene Ontology analysis

Gene Ontology analyses were conducted using the GREAT algorithm.

VISTA enhancer annotations

Relevant VISTA enhancers were annotated by at least two experts in the field. Their region of activity was ascertained on whole-mount embryos and where necessary, using sections. Gradients of activity were only defined when they were clear and when consensus was obtained between at least two observers.

Gradient modelling

We established the model associating TF binding, enhancer spatial activity gradients, and enhancer regional activity by determining the pairwise correlation of among the different factors. The TF binding combinations and other factors were intersected using custom R scripts, and the correlation matrix and plot with R ggcorrplot package. Only correlations with p < 0.01 was shown. Non-relevant associations were manually removed.

Histone mark ChIP

We used Cell Trace Yellow Cell Proliferation Kit (ThermoFisher, #C34567) or Cell Trace CFSE Cell Proliferation Kit (ThermoFisher, #C34554) to label the ventricular zone of Pax6 and Emx2 mutants and their control littermates at E12.5. FlashTag labeling was conducted by injecting 0.3 µl of 10 mM of a carboxyfluorescein succinimidyl ester (CellTrace Yellow or Cell Trace CFSE, ThermoFisher) bilaterally into the fourth ventricle of E12.5 embryos (12). Gentle manual pressure was then applied to the exterior of the embryonic head to promote even mixing of the dyes. After 20 minutes, the cortices were then dissected and papain-treated (Papain dissociation system, Worthington Biochemical Corporation) for 15-30 minutes at 37°C with rotation. After inhibiting the papain, dissociated cortical cells were resuspended in 4% FBS/1x-PBS and the singlet FTag-positive population was sorted using the BD FACS Aria III Cell Sorter at Helen Diller Cancer Building (UCSF). Approximately 200,000 cells were used for native ChIP as described in (13). Wild-type cells and mutant littermates were always injected, sorted and processed side by side using the same number of nuclei. Basically, nuclei were extracted from the sorted cells and digested for 8 mins with micrococcal nuclease (MNase, Sigma). Mono and di-nucleosomes were combined and used for ChIP of two epigenomic marks: H3 acetylated lysine 27 (H3K27ac, Abcam, ab472) and H3 trimethyl lysine-27 (H3K27me3, Active motif, 39157). After immunoprecipatation, DNA and libraries were prepared as for TF ChIP as described above.

ATAC-seq

ATAC-seq was performed on around 80,000 sorted nuclei. Basically, we fluorescently labeled the VZ of wild-type and mutant littermates using the FlashTag procedure as indicated above. After making nuclei,

the pellet was resuspended in 25uL of Tagment DNA buffer and 2uL of enzyme (Tagment DNA Enzyme, Nextera DNA Library Prep Kit, 15028211, Illumina). Tagmentation was performed at 37°C for 30 mins without shaking. Samples were then purified using MinElute columns (Qiagen), PCR-amplified for 8-10 cycles using the NEB Next High Fidelity 2x PCR Master Mix (NEB, 72 °C 5 min, 98 °C 30 s, (98 °C 10 s, 63 °C 30 s, 72 °C 60 s) per cycle, held at 72 °C). The generated amplified libraries were purified on Ampure XP Bead (Beckmann Coulter) and bioanalyzed. Sequencing was carried out on a HiSeq4000 (50 bp PE, Illumina).

PLAC-seq

PLAC-seq libraries for E12.5 cortex were prepared similar to the previously published protocol (14). 3 to 7 million cells were used for each library. If the cells appeared aggregated, they were dissociated with gentle MACS dissociator or dounce homogenizer. Each PLAC-seq library was prepared using DpnII as the restriction enzyme and Dynabeads M-280 sheep anti-rabbit IgG (Invitrogen #11203D) mixed with 5ug of H3K4me3 (04-745, Millipore) for the chromatin immunoprecipitation step. Finally, libraries were prepared with the Illumina Tru-seq adaptors and the final libraries were sent for paired-end sequencing on the HiSeq X Ten (150 bp reads).

PLAC-seq data analysis using the MAPS pipeline

We applied the MAPS pipeline (15) to detect statistically significant H3K4me3-centric long-range chromatin interactions from PLAC-seq data. We only analyzed intra-chromosomal interactions for autosomal chromosomes, and identified chromatin interactions at 10Kb bin resolution between 20Kb and 1Mb. We first mapped the raw paired-end reads (i.e. fastq file) to the mm10 reference genome using bwa mem. Next, we applied filtering steps to remove PCR duplicates, low quality reads (MAPQ <= 30) and chimeric reads (15). We then split the remaining mapped reads into short-range (distance between pair ends within 1Kb) and long-range reads (distance between pair ends between 1Kb and 1Mb). We used the short-range and long-range reads to measure protein immunoprecipitation (IP) efficiency and detect long-range chromatin interactions, respectively. In addition, we collected ChIP-seq peaks called by MACS2 from cortical cells. Among all 10Kb bin pairs, we only examined those 10Kb bin pairs in which at least one 10Kb bin overlapped with MACS2 ChIP-seq peaks, since these 10Kb bin pairs are enriched by H3K4me3 antibody during the PLAC-seq experiment (16). We fitted a positive Poisson regression model on all selected 10Kb bin pairs with more than one raw count, taking into consideration multiple bias factors including 1D genomic distance between two interacting bins, restriction enzyme cut site frequency, GC content, mappability score, and H3K4me3 antibody IP efficiency measured by the number of short-range reads in each bin. After modeling fitting, we obtained expected contact frequency, p-value and false discovery rate (FDR) for each 10Kb bin pairs. Finally, we defined a 10Kb bin pair as a statistically significant long-range chromatin interaction if the raw contact frequency >= 12, normalized contact frequency (defined as observed contact frequency/expected contact frequency) >= 2, and FDR < 0.01. We further merged adjacent significant chromatin interactions together, and defined those isolated significant chromatin interactions as singletons. We applied a more stringent FDR threshold (FDR < 0.0001) among those singletons to reduce potential false positives.

Enhancer-Gene maps (definitions and annotation strategy).

Associations were previously defined by correlation between the H3K27ac profile of putative enhancers and the total-RNA profile of annotated genes across embryonic development. Two different maps were used, based on a dataset of 29 (17) or 66 (18) samples representing time-courses considering up to 12 tissues and 7 time points. Given a list of genomic regions, a custom C++ script was used to annotate each one of these regions to the overlapping putative enhancers in these two maps (if any) and to associate them to the computationally inferred target gene.

Validated enhancers from the VISTA Enhancer Browser (derivation and annotation strategy)

Human and murine validated elements available on August 25, 2017 on the VISTA enhancer browser (http://enhancer.lbl.gov) (19). Human regions (hg19) were lifted to the mouse genome (mm10) using liftOver (20). This was run with default parameters except for *minmatch* that was set to 0.1 for mouse to human conversions and to 0.95 for mapping between mm9 and mm10. After that, the same script and strategy outlined in the previous paragraph were used to annotated a given list of genomic intervals to the regions in VISTA.

PLAC-seq data annotation

Using the same script and strategy outlined in the previous paragraphs, each end of the interaction was annotated to any overlapping: (1) VISTA element; (2) the putative target gene, as inferred separately from the two maps described in the previous paragraph; (3) TF-binding events, as inferred by ChIP-seq; in case of multiple overlapping peaks, the region was assigned the peaks with the highest enrichment score; (4) the closest gene on the linear genome, using the TSS of RefSeq genes as landmark. Coordinates of RefSeq genes were downloaded from the UCSC genome browser (19) on May 30, 2015. Merging of the resulting annotations was performed using the statistical computing environment R v3 (http://www.r-project.org).

Defining the interactome for loci in the Cortical Regionalization TF Network

We defined genomic loci based on the farthest PLAC-seq interaction between promoters and pREs for each loci. In cases in which this chromatin binding domain was restricted to only one side (upstream or downstream) of the gene body (i.e. *Bcl11a* or *Dmrt3*, Figure S5), we added a 100kb buffer on the other side of the TSS or 5'UTR.

Transgenic enhancer assays

Transgenic assays were performed according to published methods (21, 22) and the VISTA enhancer browser can be consulted here: https://enhancer.lbl.gov. A summary of the methodology can be found in Lindtner et al, 2019 (23).

Contact for Reagent and Resource Sharing

Further information and requests for resources should be directed to and will be addressed by the Lead Contacts, John L. R. Rubenstein (John.Rubenstein@ucsf.edu) and Alex S. Nord (asnord@ucdavis.edu).

Table S1: Key Resources Table

Reagent or Resource	Source	Identifier
**Antibodies**		
EMX2 (aa1-155)	This paper	N/A; Available from the authors
LHX2	S. Lomvardas	(24)
NR2F1	R&D Biosystems	PP-H8132-10
PAX6	Millipore	AB2237
PBX1	Santa Cruz	sc-888
H3K4me3	Millipore	04-745
H3K27me3	Millipore	Millipore Cat# 07-449; RRID: AB_310624
H3K27ac	Abcam	Cat# ab472
Anti-Digoxigenin-AP	Sigma-Aldrich	Cat# 11093274910
Goat anti-Rabbit IgG (H+L), Alexa Fluor 546	Thermo-Fisher	Cat# A-11035
Normal Rabbit IgG	Santa Cruz	sc-2027
**Chemicals, Peptides and Recombinant Proteins**		
DIG RNA labeling mix	Sigma-Aldrich	Cat# 11277073910
T7 RNA polymerase	Sigma-Aldrich	Cat# 10881767001
DNaseI	Sigma-Aldrich	Cat# 10104159001
Sheep Serum	Sigma-Aldrich	Cat# S2263
Blocking reagent	Sigma-Aldrich	Cat# 11096176001
BM purple	Sigma-Aldrich	Cat# 11442074001
37% Formaldehyde	Ted Pella	Cat# 18508
Dynabeads Protein G	Thermo-Fisher	Cat# 10003D
Dynabeads Protein A	Thermo-Fisher	Cat# 10001D
RNase, DNase free	Sigma-Aldrich	Cat# 11119915001

Proteinase K	Sigma-Aldrich	Cat# 3115879001
PerfeCTa SYBR Green FastMix ROX	Quanta	Cat# 95073-012
MNase	Sigma-Aldrich	Cat# N3755-50UN
Thermo Scientific™ Shandon™ Aqua-Mount Slide Mounting Media	Fisher Scientific	Cat# 14-390-5
Complete EDTA-free Protease inhibitor	Sigma-Aldrich	Cat# 11873580001
**Critical Commercial Assays**		
ChIP DNA Clean and Concentrator	Zymo Research	D5205
Ovation Ultralow DR Multiplex System	Nugen	Cat# 0344-32
2% Agarose Gel Cassette Blue Pippin	Sage Science	Cat# BDF2010
High Sensitivity DNA Reagents	Agilent Technologies	Cat# 5067-4626
MinElute PCR Purification Kit	Qiagen	28004
Nextera DNA Library Prep Kit	Illumina	#FC-121-1030
Papain Dissociation System	Worthington	LK003153
NEBNext High Fidelity 2x PCR master mix	New England Biolabs	M0541L
Micrococcal Nuclease	New England Biolabs	M0247S
CellTrace™ Yellow Cell Proliferation Kit	ThermoFisher	C34567
CellTrace™ CFSE Cell Proliferation Kit	ThermoFisher	C34554
**Experimental Models: Organisms/Strains**		
Transgenic enhancer assay: FVB	Charles River	http://www.criver.com/
ChIP-seq: CD1	Charles River	http://www.criver.com/

Emx2$^{-/-}$	JAX	MGI:2148858; (2)
Emx1::Cre; Nr2f1$^{-/-}$*, Nr2f2*$^{lox/lox}$	Tang et al. 2012	(4, 5)
Nr2f1$^{-/-}$	Zhou et al., 2001	(3)
Pax6$^{-/-}$	JAX	Stock #000391; (1)

A

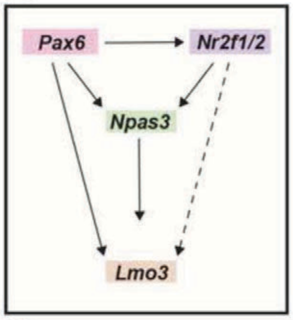

B

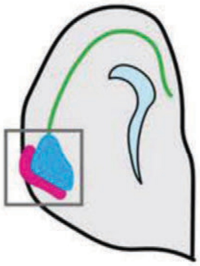

C

pRE coordinates (mm9)	Features of genomic locus	ChIP binding		Differential epigenomic marks in WT vs. Mutant						pRE/TSS interaction
				Pax6 mutant			*Nr2f1* mutant			
		NR2F1	PAX6	27ac	27me3	ATAC	27ac	27me3	ATAC	
**Nr2f1 interactome**										
chr13:77535859-77537676	hs273 Enhancer	x		Same		Same			Same	x
chr13:78031585-78032706	hs271 Enhancer	x	x	Loss		Same	Same		Same	x
chr13:78033028-78033775		x	x	Loss		Same	Loss		Same	x
chr13:78238631-78240287			x	Loss		Loss	Same		Same	x
chr13:78301285-78303743		x		Loss		Same	Same	Same	Same	x
chr13:78318156-78320079		x	x	Loss	Gain	Same	Loss	Loss	Same	x
chr13:78336209-78338805	TSS		x	Loss	Same	Same	Loss	Loss	Loss	x
chr13:78339478-78340485		x				Same		Loss	Same	x
chr13:78341891-78344303		x		Same		Same	Same	Loss	Same	x
chr13:78565590-78568155			x	Loss		Same	Same		Same	x
chr13:78583826-78587247	hs1550 Enhancer	x	x	Loss		Loss	Same		Same	x
chr13:78719884-78721593	hs1172 Enhancer	x	x			Same			Same	x
**Npas3 Interactome**										
chr12:54162201-54164479	mm1790 Enhancer			Loss		Same	Loss		Same	x
chr12:54224810-54226545			x			Same			Same	
chr12:54347665-54350320	TSS			Loss		Same	Loss		Same	x
chr12:54353494-54355559	Gene Body		x	Loss		Same	Loss		Same	x
chr12:54391045-54394848	Gene Body	x		Loss		Same	Loss			x
chr12:54548080-54550100	mm1789 Enhancer	x	x			Loss			Same	x
chr12:54593760-54594932	Gene Body	x				Same			Same	x
chr12:54723872-54727081	hs527 Enhancer	x	x	Loss		Loss	Same?		Same	x
**Lmo3 Interactome**										
chr6:138368197-138369679	mm1199 Enhancer		x	Loss		Loss	Loss			
chr6:138372851-138374454	Gene Body	x	x			Same			Same	
chr6:138375177-138376219	Gene Body		x			Loss			Same	
chr6:138696588-138711025	hs1532 Enhancer	x	x		Loss	Loss		Same	Same	

Figure S1. Genetic circuitry controlling the patterning of the rostral latero-ventral pallium (LVP)
(A) Schematic of transcriptional control of rostral LVP development.

(B) Depiction of the rostral LVP at E16.5. Piriform cortex (*Lmo3*$^{+}$ and *Npas3*$^{+}$) is pink. Endopiriform cortex and claustrum (*Nurr1*$^{+}$) are blue. Subplate is green.

(C) Table of regulatory elements (pREs) around the *Nr2f1, Npas3* and *Lmo3* loci, that may participate in rostral LVP patterning Shown are NR2F1 and PAX6 ChIP-seq peaks, differential epigenomic peaks in the *Nr2f1*$^{-/-}$ *and Pax6*$^{-/-}$ and pRE/TSS interactions (see Figures 4, 6, 7).

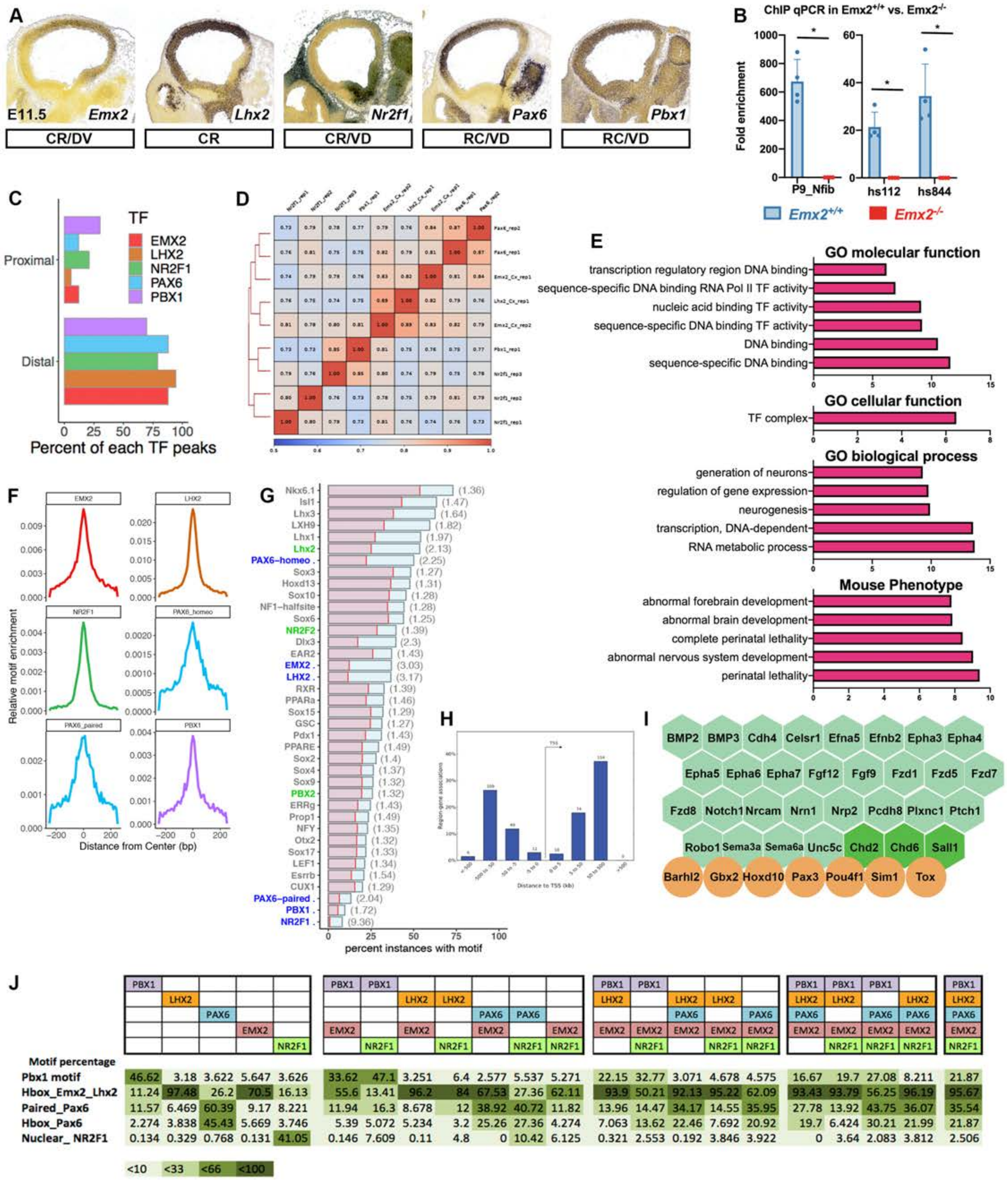

A
E11.5 Emx2
Lhx2
Nr2f1
Pax6
Pbx1
CR/DV
CR
CR/VD
RC/VD
RC/VD
B
ChIP qPCR in Emx2+/+ vs. Emx2-/-
Fold enrichment
P9_Nfib
hs112
hs844
Emx2+/+
Emx2-/-
C
TF
EMX2
LHX2
NR2F1
PAX6
PBX1
Proximal
Distal
Percent of each TF peaks
D
E
GO molecular function
transcription regulatory region DNA binding
sequence-specific DNA binding RNA Pol II TF activity
nucleic acid binding TF activity
sequence-specific DNA binding TF activity
DNA binding
sequence-specific DNA binding
GO cellular function
TF complex
GO biological process
generation of neurons
regulation of gene expression
neurogenesis
transcription, DNA-dependent
RNA metabolic process
Mouse Phenotype
abnormal forebrain development
abnormal brain development
complete perinatal lethality
abnormal nervous system development
perinatal lethality
F
EMX2
LHX2
NR2F1
PAX6_homeo
PAX6_paired
PBX1
Relative motif enrichment
Distance from Center (bp)
G
Nkx6.1 (1.36)
Isl1 (1.47)
Lhx3 (1.64)
LXH9 (1.82)
Lhx1 (1.97)
Lhx2 (2.13)
PAX6-homeo (2.25)
Sox3 (1.27)
Hoxd13 (1.31)
Sox10 (1.28)
NF1-halfsite (1.28)
Sox6 (1.25)
NR2F2 (1.39)
Dlx3 (2.3)
EAR2 (1.43)
EMX2 (3.03)
LHX2 (3.17)
RXR (1.39)
PPARa (1.46)
Sox15 (1.29)
GSC (1.27)
Pdx1 (1.43)
PPARE (1.49)
Sox2 (1.4)
Sox4 (1.37)
Sox9 (1.32)
PBX2 (1.32)
ERRg (1.43)
Prop1 (1.49)
NFY (1.35)
Otx2 (1.32)
Sox17 (1.33)
LEF1 (1.34)
Esrrb (1.54)
CUX1 (1.29)
PAX6-paired (2.04)
PBX1 (1.72)
NR2F1 (9.36)
percent instances with motif
H
Region-gene associations
Distance to TSS (kb)
I
BMP2 BMP3 Cdh4 Celsr1 Efna5 Efnb2 Epha3 Epha4
Epha5 Epha6 Epha7 Fgf12 Fgf9 Fzd1 Fzd5 Fzd7
Fzd8 Notch1 Nrcam Nrn1 Nrp2 Pcdh8 Plxnc1 Ptch1
Robo1 Sema3a Sema6a Unc5c Chd2 Chd6 Sall1
Barhl2 Gbx2 Hoxd10 Pax3 Pou4f1 Sim1 Tox
J
Motif percentage
Pbx1 motif 46.62 3.18 3.622 5.647 3.626 33.62 47.1 3.251 6.4 2.577 5.537 5.271 22.15 32.77 3.071 4.678 4.575 16.67 19.7 27.08 8.211 21.87
Hbox_Emx2_Lhx2 11.24 97.48 26.2 70.5 16.13 55.6 13.41 96.2 84 67.53 27.36 62.11 93.9 50.21 92.13 95.22 62.09 93.43 93.79 56.25 96.19 95.67
Paired_Pax6 11.57 6.469 60.39 9.17 8.221 11.94 16.3 8.678 12 38.92 40.72 11.82 13.96 14.47 34.17 14.55 35.95 27.78 13.92 43.75 36.07 35.54
Hbox_Pax6 2.274 3.838 45.43 5.669 3.746 5.39 5.072 5.234 3.2 25.26 27.36 4.274 7.063 13.62 22.46 7.692 20.92 19.7 6.424 30.21 21.99 21.87
Nuclear_ NR2F1 0.134 0.329 0.768 0.131 41.05 0.146 7.609 0.11 4.8 0 10.42 6.125 0.321 2.553 0.192 3.846 3.922 0 3.64 2.083 3.812 2.506
<10 <33 <66 <100

Figure S2. Characterization of EMX2, LHX2, NR2F1, PAX6, and PBX1 combinatorial binding

(A) ISH cortical expression gradients of TFs used for ChIP at E11.5. *Lhx2* has a caudorostral (CR) gradient. *Emx2 has a CR and dorsoventral (DV) gradient. Nr2f1* has a CR and ventrodorsal (VD) gradient. *Pax6* and *Pbx1* have a rostrocaudal (RC) and VD gradient.

(B) ChIP-qPCR on *Emx2*$^{+/+}$ (blue bars) and *Emx2*$^{-/-}$ (red bars) cortices at E15.5 shows significantly decreased enrichment of binding at genomic targets (enhancers around *Nfib*, *Dmrt3* and *Sp8*) in *Emx2*$^{-/-}$.

(C) Proximal (near promoter) vs. Distal binding for each TF ChIP-seq

(D) Heatmap showing pairwise Pearson Correlation for genome-wide coverage values for each TF ChIP-seq replicate.

(E) Enrichment of functional annotation terms (GO) for genomic loci showing combinatorial binding of 5 TFs by ChIP-seq.

(F) Relative motif enrichment for primary binding DNA motifs of EMX2, LHX2, NR2F1, PAX6 and PBX1 ChIP-seq across all pREs.

(G) Motif enrichment of all *de novo* motifs in pREs of the TF ChIPs.

(H) Counts of distance from TSS for loci showing combinatorial binding of 5TFs by ChIP-seq.

(I) Non-TF genes important in cortical development showing co-binding of 5TFs by ChIP-seq. Light green are genes with known functions during cortical development; Dark green are chromatin modifiers; Orange are TFs with expression in other brain regions.

(J) Analysis showing percentage of peaks with motif enrichment of primary binding motifs (described in Figure 4C) for each unique or combinatorial binding of TFs by ChIP-seq. Abbreviations: Hbox: homeobox.

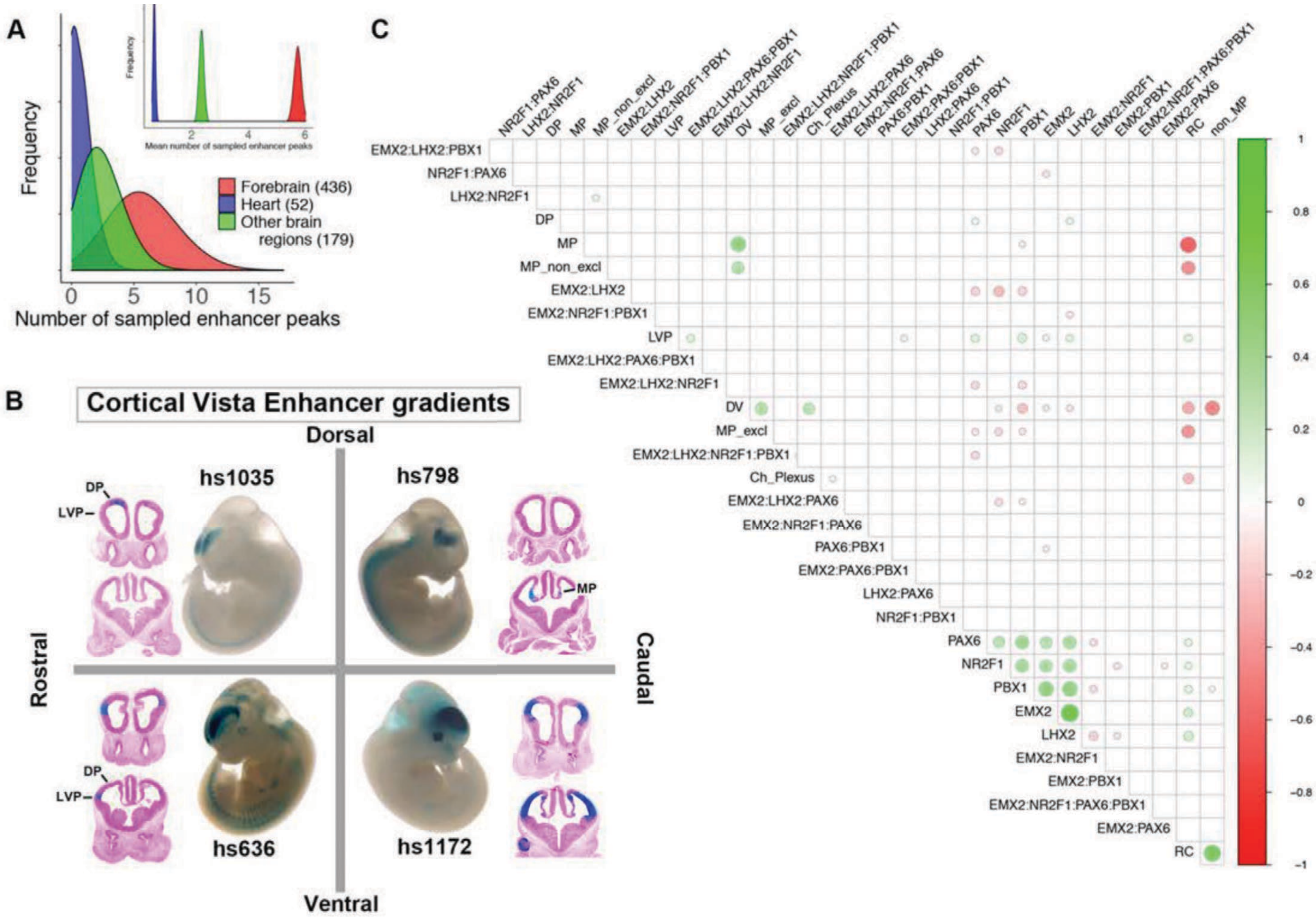

A
Frequency
Mean number of sampled enhancer peaks
Forebrain (436)
Heart (52)
Other brain regions (179)
Number of sampled enhancer peaks
B
Cortical Vista Enhancer gradients
Dorsal
Ventral
Rostral
Caudal
hs1035
hs798
hs636
hs1172
DP
LVP
MP
C
EMX2:LHX2:PBX1
NR2F1:PAX6
LHX2:NR2F1
DP
MP
MP_non_excl
EMX2:LHX2
EMX2:NR2F1:PBX1
LVP
EMX2:LHX2:PAX6:PBX1
EMX2:LHX2:NR2F1
DV
MP_excl
EMX2:LHX2:NR2F1:PBX1
Ch_Plexus
EMX2:LHX2:PAX6
EMX2:NR2F1:PAX6
PAX6:PBX1
EMX2:PAX6:PBX1
LHX2:PAX6
NR2F1:PBX1
PAX6
NR2F1
PBX1
EMX2
LHX2
EMX2:NR2F1
EMX2:PBX1
EMX2:NR2F1:PAX6:PBX1
EMX2:PAX6
RC
non_MP
1
0.8
0.6
0.4
0.2
0
−0.2
−0.4
−0.6
−0.8
−1

Figure S3. Using VISTA enhancers to model how combinatorial binding of EMX2, LHX2, NR2F1, PAX6, and PBX1 predicts cortical activity and graded expression in the developing pallial VZ.

(A) Plot showing how likely VISTA enhancer loci bound by the TFs are forebrain-active as opposed to active in other brain regions and elsewhere (heart as a surrogate). Main plot shows the frequency of occurrence, while the upper one depicts the comparison of mean number of enhancers hit by sampling, showing significant difference among different enhancer classes ($p < 0.01$).

(B) Examples of cortical VISTA enhancers with graded patterns of activity. The wholemounts and sections for each example are placed along the central grid according to their annotated gradient of activity in the developing pallium. hs1035 has a DV and RC gradient; hs798 has a DV and CR gradient; hs636 has a RC and VD gradient; and finally, hs1172 has a CR and VD gradient.

(C) Correlalogram showing the expansion of the modeling presented in Figure 4D showing the different combinations of TF binding and their predictions of gradients of activity ($p < 0.01$)

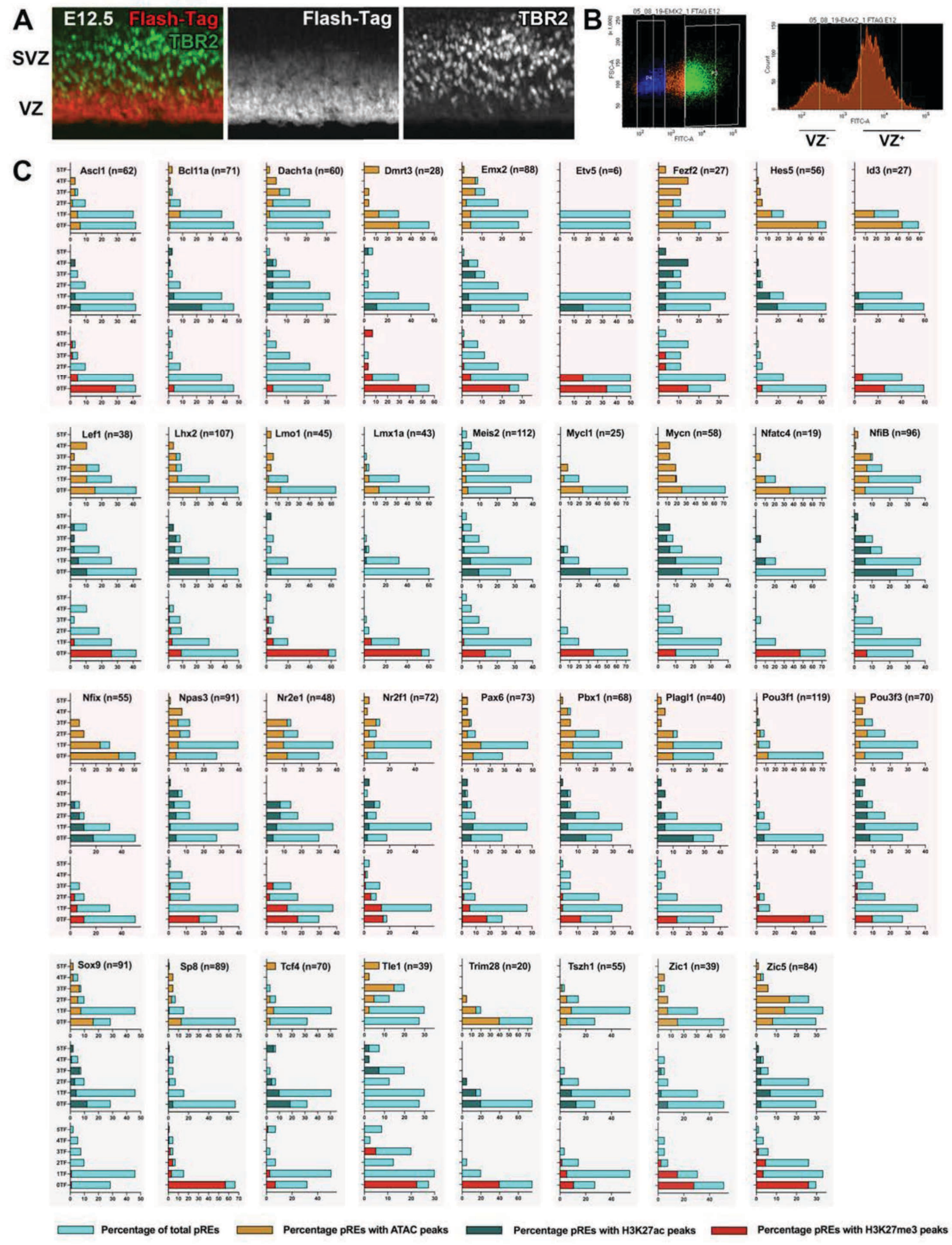

A
E12.5 Flash-Tag TBR2
SVZ
VZ
Flash-Tag
TBR2
B
05_08_19-EMX2_1 FTAG E12
FSC-A
FITC-A
Count
VZ-
VZ+
C
Ascl1 (n=62)
Bcl11a (n=71)
Dach1a (n=60)
Dmrt3 (n=28)
Emx2 (n=88)
Etv5 (n=6)
Fezf2 (n=27)
Hes5 (n=56)
Id3 (n=27)
Lef1 (n=38)
Lhx2 (n=107)
Lmo1 (n=45)
Lmx1a (n=43)
Meis2 (n=112)
Mycl1 (n=25)
Mycn (n=58)
Nfatc4 (n=19)
NfiB (n=96)
Nfix (n=55)
Npas3 (n=91)
Nr2e1 (n=48)
Nr2f1 (n=72)
Pax6 (n=73)
Pbx1 (n=68)
Plagl1 (n=40)
Pou3f1 (n=119)
Pou3f3 (n=70)
Sox9 (n=91)
Sp8 (n=89)
Tcf4 (n=70)
Tle1 (n=39)
Trim28 (n=20)
Tszh1 (n=55)
Zic1 (n=39)
Zic5 (n=84)
5TF
4TF
3TF
2TF
1TF
0TF
Percentage of total pREs
Percentage pREs with ATAC peaks
Percentage pREs with H3K27ac peaks
Percentage pREs with H3K27me3 peaks

Figure S4. Epigenomic profiling of CRTFN genes (Cortical Regionalization TF Network) in the cortical VZ.

(A) Histology of Flash-Tag staining of the cortical VZ at E12.5; note that the Flash-Tag labeled VZ cells do not overlap with TBR2 immunostained SVZ progenitors.

(B) Example FACs plots showing data for Flash-Tag (CFSE) positive progenitor cells prepared from the E12.5 cortex. The histogram of FITC counts shows a bi-modal FITC negative population (VZ^-) and a FITC positive population (VZ^+). The dot plot depicting FSC vs. FITC shows the gating which was used to collect the $FITC^+$ (VZ^+) population. Black bar above VZ^+ correspond to the cells collected for further analysis.

(C) Overview of epigenomic enrichment in pREs with differential combinatorial TF binding (0-5 TFs) for CRTFN TF gene loci. Number of pREs at each locus are in brackets (n=#pREs). Percentage of total pREs are indicated by a teal histogram bar. Percentage of pREs with the following epigenetic marks are indicated by the following colors: ATAC = yellow; H3K27ac = dark green; H3K27me3 = red. See Figure S4A which summarizes this data for each TF locus.

A

		# pREs	TF ChIP-Seq					Ratio H3K27ac / H3K27me3	VZ Epigenomic Marks		
			Emx2	Lhx2	Nr2f1	Pax6	Pbx1		H3K27ac	H3K27me3	ATAC
			**TFs with Rostral to Caudal Gradient**								
Lmo1	★	45	20.0	17.8	13.3	15.6	4.4	0.1	8.9	68.9	31.1
Pou3f1		119	14.3	10.9	13.4	5.9	7.6	0.2	10.9	63.9	23.5
Zic1	O	39	23.1	20.5	15.4	20.5	2.6	0.3	12.8	46.2	38.5
Etv5		6	16.7	16.7	0.0	16.7	0.0	0.3	16.7	50.0	0.0
Tle1	O	39	42.5	42.5	27.5	35.0	15.0	0.5	12.5	27.5	32.5
Zic5	O	84	46.4	21.4	26.2	17.9	11.9	0.5	17.9	35.7	48.8
Nr2e1	O	48	34.0	16.0	44.0	20.0	2.0	0.7	26.0	36.0	44.0
Npas3		91	39.6	24.2	35.2	19.8	17.6	1.0	19.8	19.8	30.8
Pax6	★	73	35.6	23.3	26.0	28.8	9.6	1.1	26.0	24.7	39.7
Mycl1	★	25	8.0	0.0	24.0	0.0	4.0	1.1	40.0	36.0	36.0
Meis2		112	37.5	26.8	33.0	23.2	13.4	1.7	27.7	16.1	22.3
Pbx1		68	44.1	30.9	26.5	13.2	13.2	2.6	38.2	14.7	35.3
Pou3f3		70	37.1	25.7	41.4	27.1	14.3	2.9	37.1	12.9	31.4
Sox9		91	30.8	22.0	37.4	18.7	13.2	13.5	29.7	2.2	40.7
			**TFs with Caudal to Rostral Gradient**								
Sp8	✚	89	22.6	14.3	14.3	10.7	7.1	0.1	7.1	69.0	28.6
Dmrt3	O	28	18.5	14.8	22.2	18.5	11.1	0.2	14.8	63.0	51.9
Nfatc4	O	19	5.3	5.3	26.3	0.0	0.0	0.3	15.8	47.4	52.6
Emx2	O	88	47.7	36.4	25.0	12.5	9.1	0.6	18.2	30.7	26.1
Nr2f1	O	72	36.1	26.4	43.1	22.2	13.9	0.6	22.2	37.5	31.9
Lef1	✚	38	28.9	26.3	23.7	21.1	13.2	0.8	23.7	28.9	50.0
Tszh1		55	25.5	20.0	25.5	12.7	10.9	1.3	23.6	18.2	21.8
Fezf2	✚	27	48.1	44.4	33.3	22.2	18.5	1.7	37.0	22.2	63.0
Nfix	✚	55	18.2	7.3	40.0	7.3	1.8	2.0	40.0	20.0	80.0
Lhx2		107	21.5	19.6	25.2	10.3	11.2	3.2	50.5	15.9	43.9
Tcf4	✚	70	34.8	20.3	26.1	20.3	8.7	3.4	39.1	11.6	18.8
Mycn		58	32.8	25.9	39.7	10.3	8.6	4.2	43.1	10.3	48.3
Dach1		60	43.3	45.0	18.3	20.0	11.7	4.5	15.0	3.3	18.3
NfiB	✚	96	44.8	25.0	19.8	16.7	8.3	6.7	49.0	7.3	34.4
Bcl11a	✚	71	25.4	14.1	22.5	15.5	5.6	7.7	32.4	4.2	16.9
			**TFs with Ventral-Dorsal Gradient**								
Ascl1	★	62	29.0	11.3	24.2	21.0	1.6	0.3	12.9	37.1	19.4
Hes5	★	56	16.1	7.1	23.2	5.4	1.8	7.3	39.3	5.4	82.1
			**TFs with Dorsal-Ventral Gradient**								
Trim28	✚	20	5.0	0.0	10.0	5.0	10.0	1.0	40.0	40.0	60.0
Plagl1	✚	40	28.2	28.2	23.1	20.5	7.7	3.4	43.6	12.8	41.0
			**TFs with Expression in MP**								
Lmx1a		43	18.6	14.0	9.3	2.3	4.7	0.1	4.7	60.5	20.9
Id3		27	0.0	3.7	37.0	0.0	0.0	0.3	11.1	33.3	63.0

★ Ventral-Dorsal
✚ Dorsal-Ventral
O Medial Pallium

B

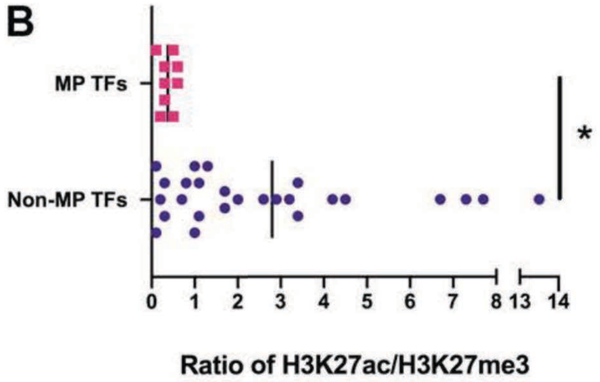

C

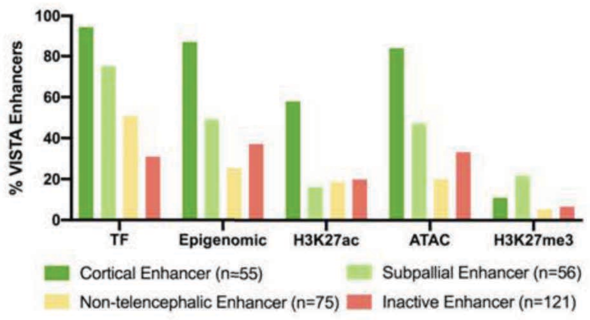

Figure S5. Epigenomic profiling of the cortical VZ shows a diminished H3K27ac/H3K27me3 ratio over pREs for MP TFs

(A) Table showing percentage enrichment of TF binding and epigenomic marks of pREs at each locus of the Cortical Regionalization TF Network. A H3K27ac/H3K27me3 ratio is calculated for each locus.

(B) Statistical analysis shows that the H3K27ac/H3K27me3 ratio is significantly lower for pREs in genomic loci of TFs with MP expression (red) compared to pREs for the non-MP TFs (purple) ($p<0.05$, unpaired two-tailed t-test).

(C) Plot of genomic features (TF binding, Epigenomic marks, H3K27ac, H3K27me3, ATAC) over VISTA enhancers that have pallial (dark green), subpallial (light green), non-telencephalic (yellow) and no activity (red) at E11.5.

Arx

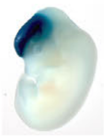

hs122

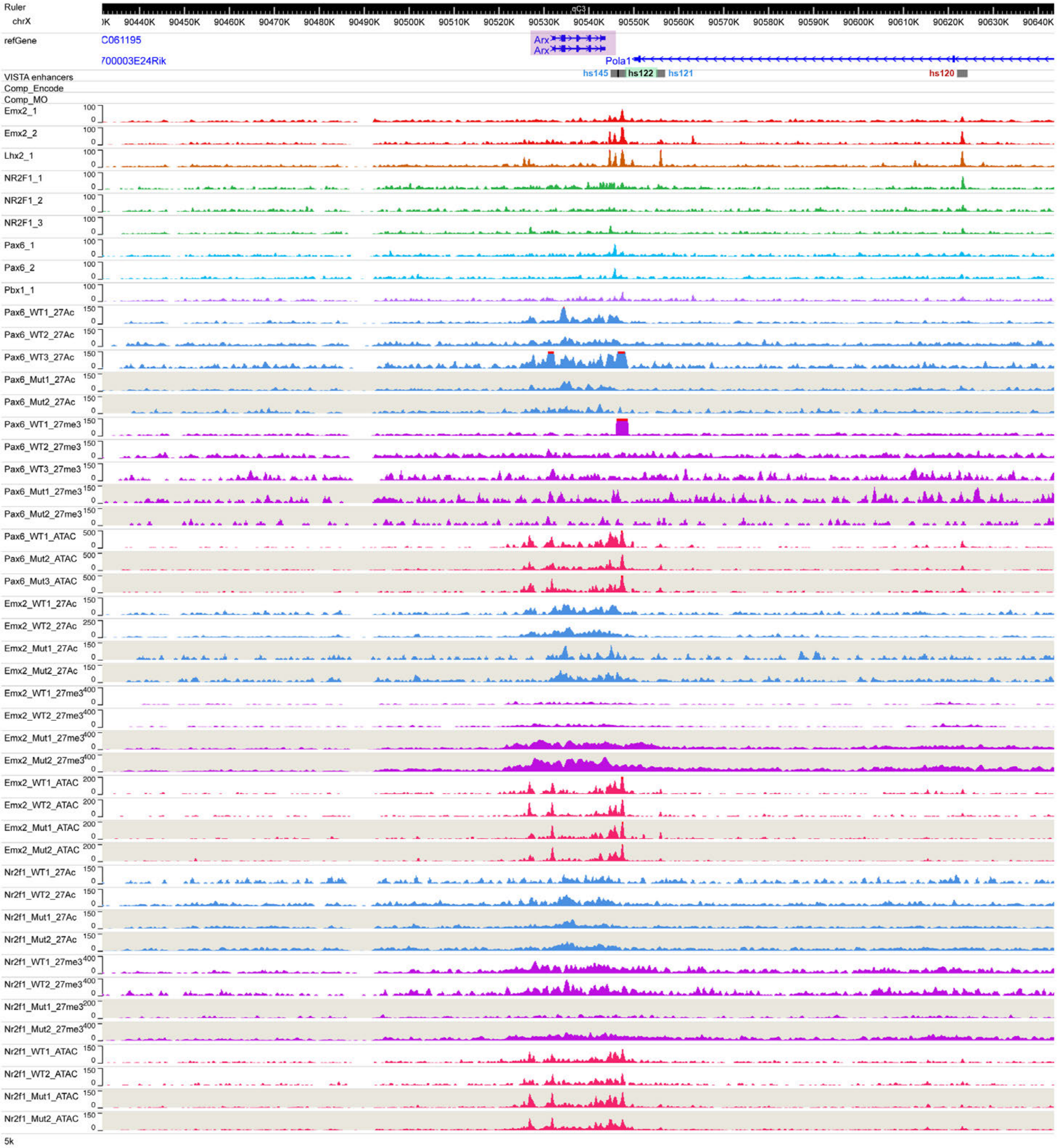

Ruler
chrX
90440K 90450K 90460K 90470K 90480K 90490K 90500K 90510K 90520K 90530K 90540K 90550K 90560K 90570K 90580K 90590K 90600K 90610K 90620K 90630K 90640K
refGene
C061195
700003E24Rik
Arx
Arx
Pola1
VISTA enhancers
hs145
hs122
hs121
hs120
Comp_Encode
Comp_MO
Emx2_1
Emx2_2
Lhx2_1
NR2F1_1
NR2F1_2
NR2F1_3
Pax6_1
Pax6_2
Pbx1_1
Pax6_WT1_27Ac
Pax6_WT2_27Ac
Pax6_WT3_27Ac
Pax6_Mut1_27Ac
Pax6_Mut2_27Ac
Pax6_WT1_27me3
Pax6_WT2_27me3
Pax6_WT3_27me3
Pax6_Mut1_27me3
Pax6_Mut2_27me3
Pax6_WT1_ATAC
Pax6_Mut2_ATAC
Pax6_Mut3_ATAC
Emx2_WT1_27Ac
Emx2_WT2_27Ac
Emx2_Mut1_27Ac
Emx2_Mut2_27Ac
Emx2_WT1_27me3
Emx2_WT2_27me3
Emx2_Mut1_27me3
Emx2_Mut2_27me3
Emx2_WT1_ATAC
Emx2_WT2_ATAC
Emx2_Mut1_ATAC
Emx2_Mut2_ATAC
Nr2f1_WT1_27Ac
Nr2f1_WT2_27Ac
Nr2f1_Mut1_27Ac
Nr2f1_Mut2_27Ac
Nr2f1_WT1_27me3
Nr2f1_WT2_27me3
Nr2f1_Mut1_27me3
Nr2f1_Mut2_27me3
Nr2f1_WT1_ATAC
Nr2f1_WT2_ATAC
Nr2f1_Mut1_ATAC
Nr2f1_Mut2_ATAC
5k

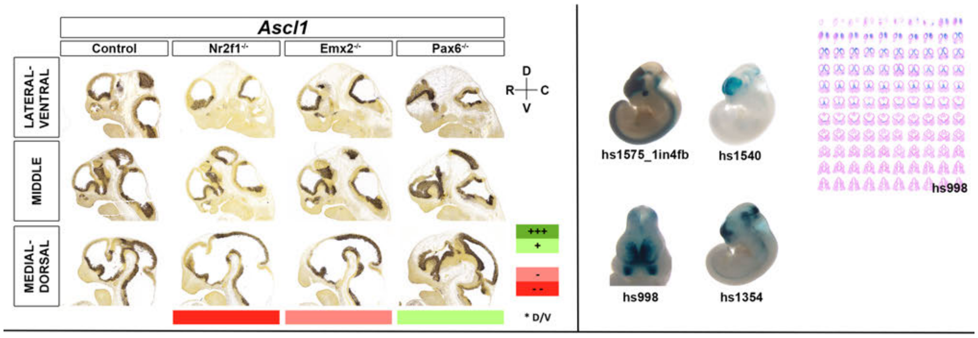

		Motifs					TF ChIP Seq					Differential epigenomic marks in WT vs. Mutant									Plac	Computation	Genomic features
												Pax6 mutant			Emx2 mutant			Nr2f1 mutant					
		Emx2 HB	Nr2f1	Pax6 Hb	Pax6 P	Pbx1	Emx2	Lhx2	Nr2f1	Pax6	Pbx1	27Ac	27me3	ATAC	27Ac	27me3	ATAC	27Ac	27me3	ATAC			
Ascl1	chr10:86400000-87800000																						
1	chr10:86434673-86435586								x											Small Gain	x		
2	chr10:86487025-86488510						x										Gain			Small Gain	x		
3	chr10:86563808-86565878	x		x	x		x	x	x	x		Same		Same	Loss		Gain	Same		Same	x		
4	chr10:86691462-86694122															Gain					x		5"UTR 1700113h08Rik
5	chr10:86906349-86912592	x					x					Same		Loss	Loss		Same	Same		Same		x	hs1540
6	chr10:87387234-87391021			x	x		x		x	x			Small Loss	Same		Small Loss	Same		Same	Same			Igf1 Gene Body
7	chr10:87605288-87611277	x								x		Loss		Loss	Same		Same	Same		Same	x		TSS Nup37
8	chr10:87660733-87665800											Loss		Loss	Same		Same			Same	x		TSS Cdc53

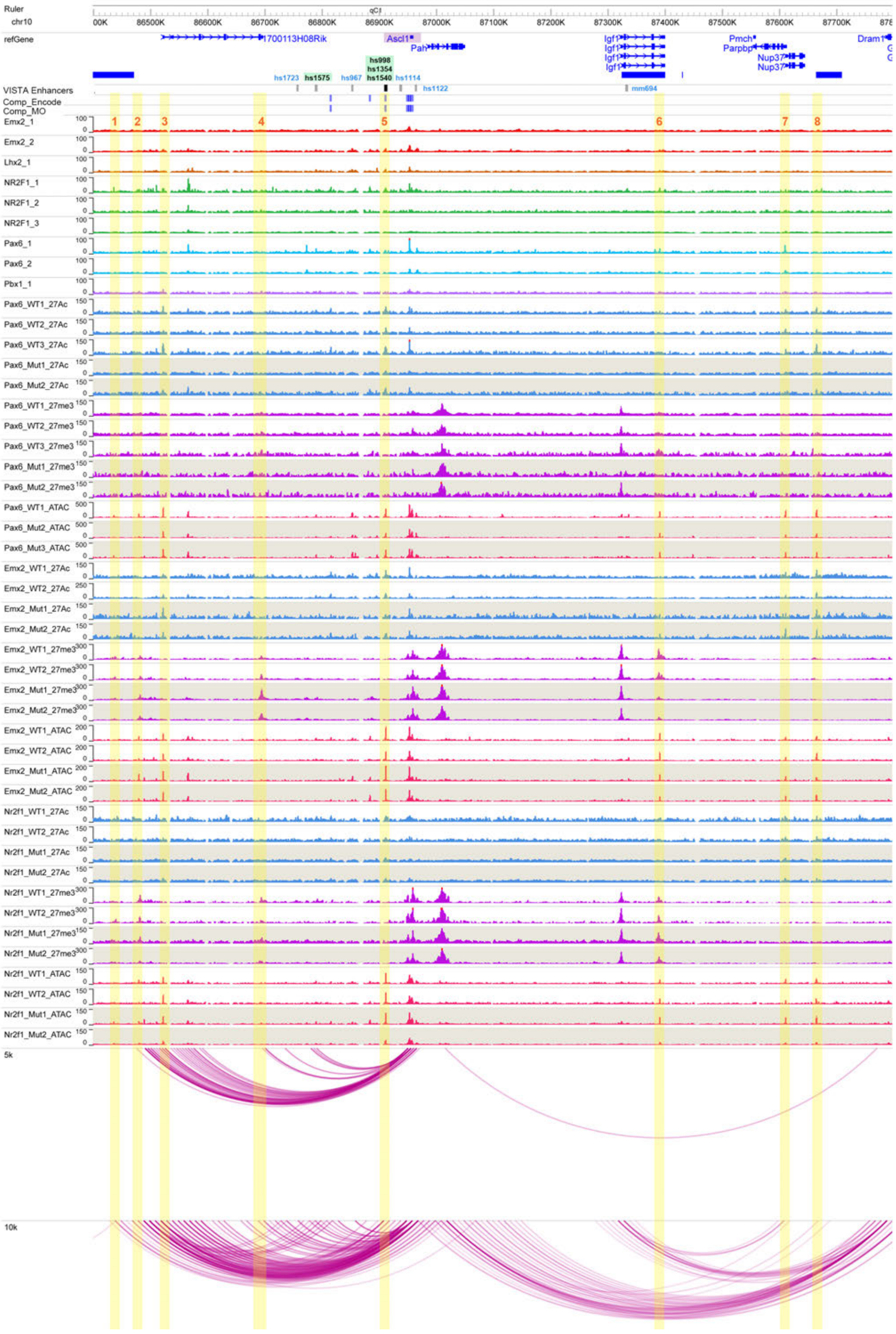

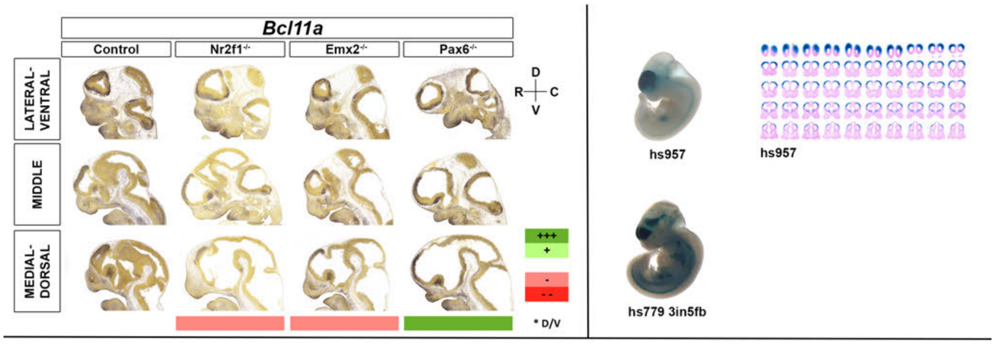

Bcl11a	chr11:23875000-25000000	Motifs: Emx2 HB	Motifs: Nr2f1	Motifs: Pax6 Hb	Motifs: Pax6 P	Motifs: Pbx1	TF ChIP Seq: Emx2	TF ChIP Seq: Lhx2	TF ChIP Seq: Nr2f1	TF ChIP Seq: Pax6	TF ChIP Seq: Pbx1	Differential epigenomic marks in WT vs. Mutant: Pax6 mutant 27Ac	Pax6 mutant 27me3	Pax6 mutant ATAC	Emx2 mutant 27Ac	Emx2 mutant 27me3	Emx2 mutant ATAC	Nr2f1 mutant 27Ac	Nr2f1 mutant 27me3	Nr2f1 mutant ATAC	Plac	Computation	Genomic features
1	chr11:23949196-23950435											Loss		Same	Loss		Same	Loss		Same		x	
2	chr11:23962200-23963523												Same			Gain			Same				hs1176
3	chr11:23973144-23974804		x						x							Gain						x	
4	chr11:23976082-23978715								x			Loss		Same	Same		Same	Loss		Same		x	TSS Bcl11a
5	chr11 23995402 23996537						x	x	x	x	x	Loss		Same	Same		Loss	Same		Same		x	hs957
6	chr11:24270356-24271960						x	x	x	x	x	Loss		Same	Loss	Gain	Same	Loss		Same	x		hs399
7	chr11:24327290-24328274						x		x					Same			Gain			Small Gain	x		hs779

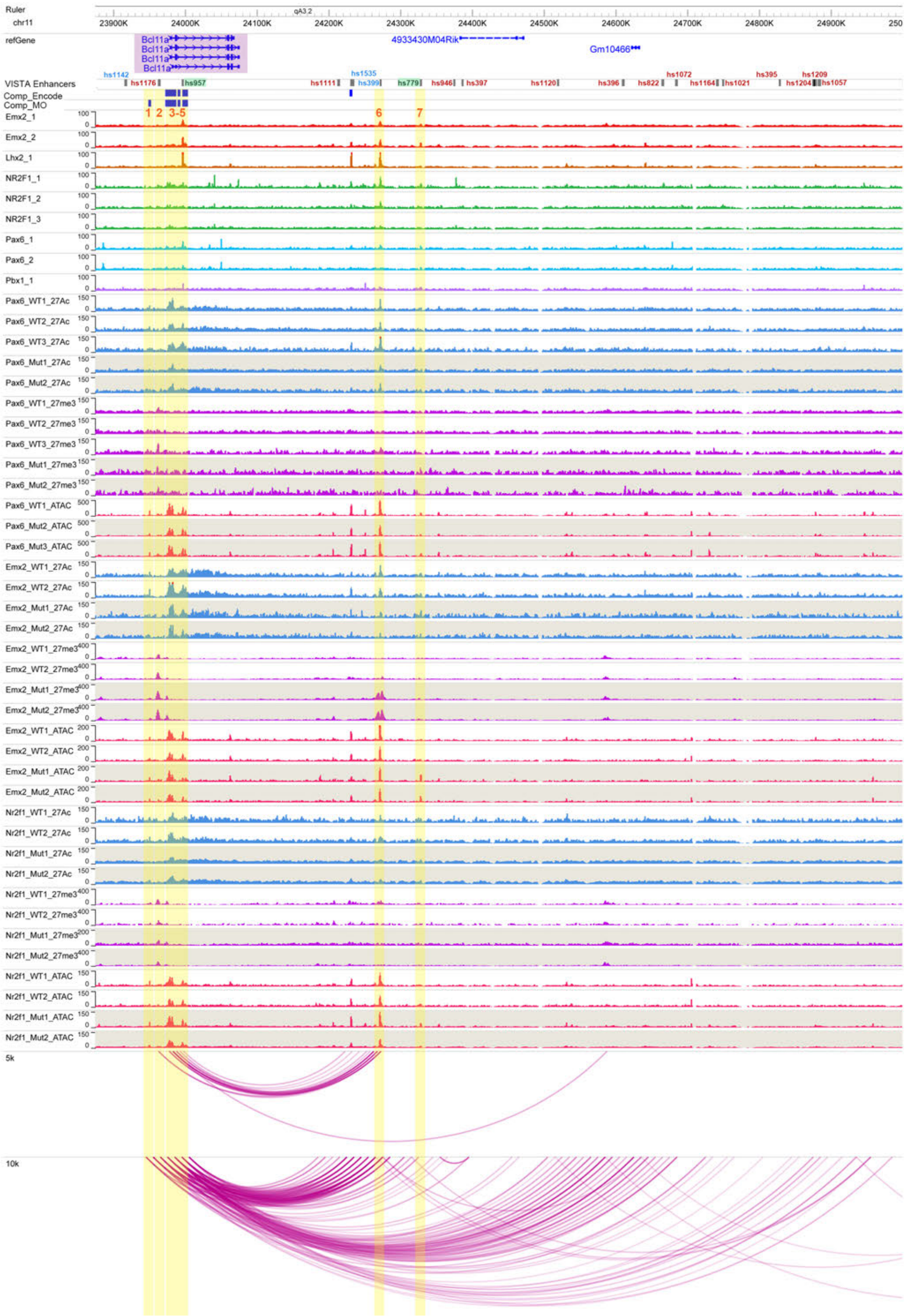

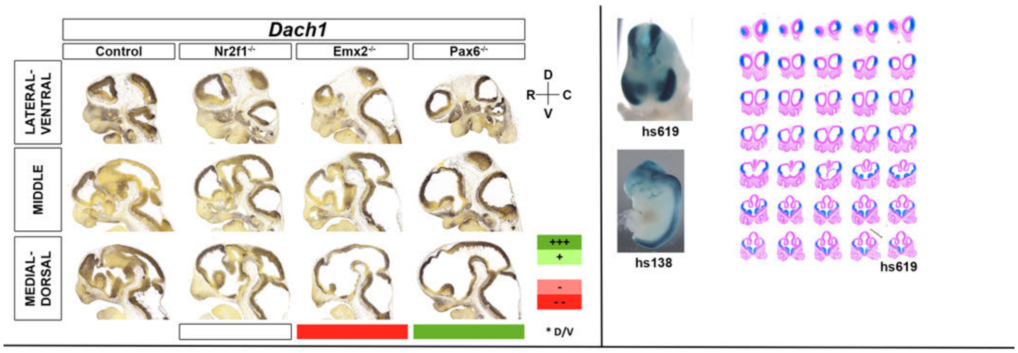

		Motifs					TF ChIP Seq					Differential epigenomic marks in WT vs. Mutant									Plac	Computation	Genomic features
												Pax6 mutant			Emx2 mutant			Nr2f1 mutant					
		Emx2 HB	Nr2f1	Pax6 Hb	Pax6 P	Pbx1	Emx2	Lhx2	Nr2f1	Pax6	Pbx1	27Ac	27me3	ATAC	27Ac	27me3	ATAC	27Ac	27me3	ATAC			
Dach1a	chr14:97700000-99400000																						
1	chr14:97794887-97795910						x	x	x					Small Gain			Small Gain			Small Gain			
2	chr14:97936954-97937320	x					x	x						Small Gain							x		hs129
3	chr14:97984506-97988219						x	x	x					Same		Gain	Same			Same	x		
4	chr14:98213227-98214488	x		x	x		x	x		x		Same?		Same	Loss		Loss	Same?		Same	x		
5	chr14:98471567-98473364	x				x	x	x	x		x	Small Loss		Same	Loss	Gain	Same	Same		Same	x		
6	chr14:98507543-98508878	x					x	x		x	x	Same		Same	Loss		Same?	Same		Same	x		
7	chr14:98522021-98522688	x				x	x	x	x	x	x			Loss			Loss			Same			
8	chr14:98533233-98535459	x		x	x		x	x	x	x				Loss			Loss			Same			
9	chr14:98564922-98570142								x			Loss		Same	Loss	Gain	Same	Same		Same			Dach1a TSS
10	chr14:98571770-98573377	x					x	x			x			Same		Gain	Loss			Same		x	
11	chr14:98787230-98787662	x					x	x			x			Same			Loss			Same			
12	chr14:98935571-98938292						x		x					Gain									hs131
13	chr14:99256151-99264870															Small Gain					x		

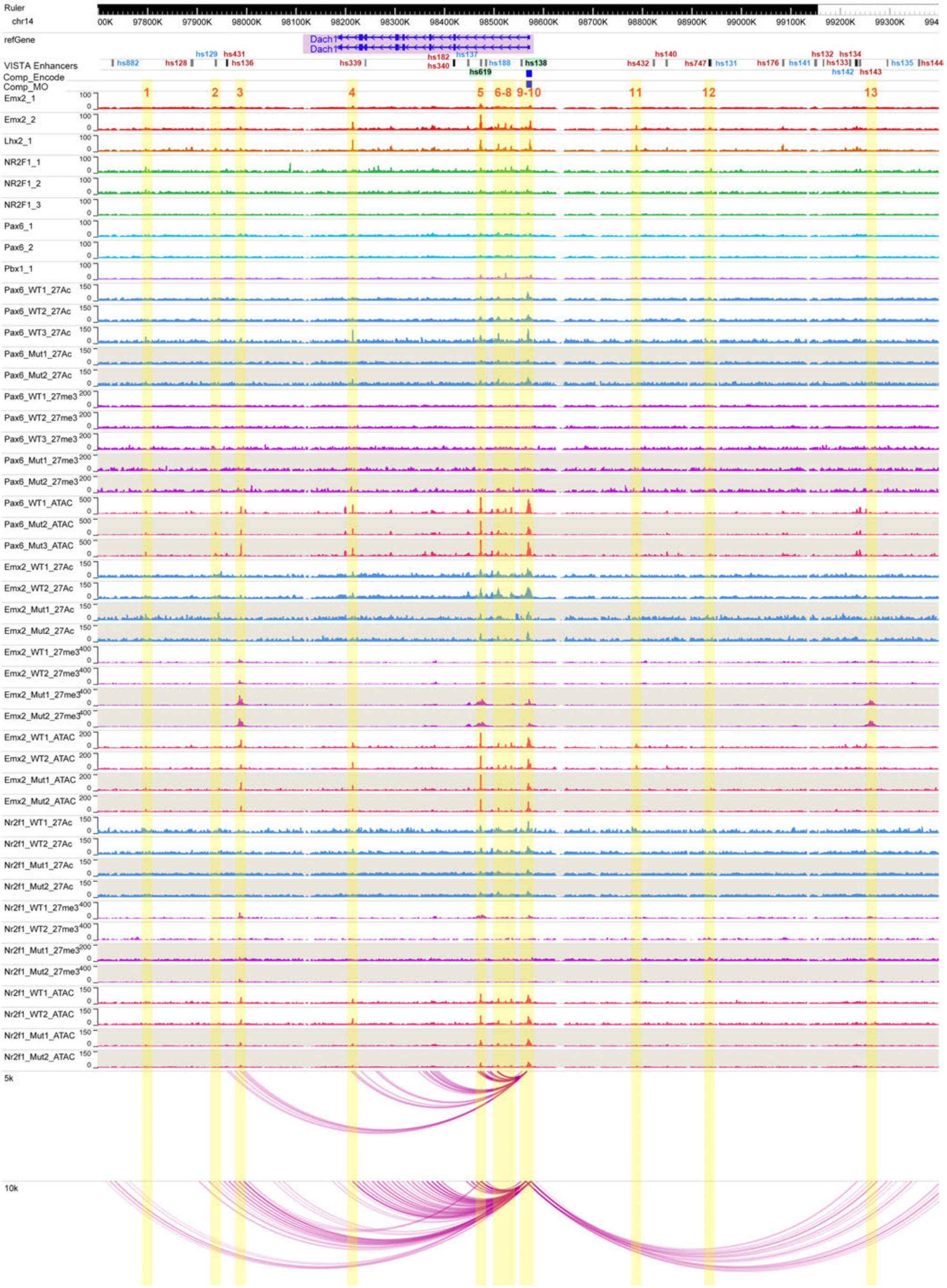

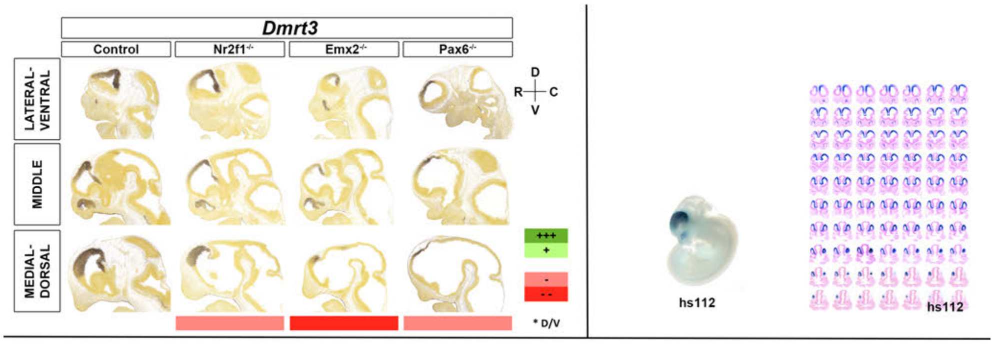

		Motifs					TF ChIP Seq					Differential epigenomic marks in WT vs. Mutant									Plac	Computation	Genomic features
												Pax6 mutant			Emx2 mutant			Nr2f1 mutant					
		Emx2 HB	Nr2f1	Pax6 Hb	Pax6 P	Pbx1	Emx2	Lhx2	Nr2f1	Pax6	Pbx1	27Ac	27me3	ATAC	27Ac	27me3	ATAC	27Ac	27me3	ATAC			
Dmrt3	chr19:25014000-25800000																						
Ctrl1	chr19:25579959-25581177												Same	Same		Same	Same		Same	Same			Dmrt1 TSS
1	chr19:25673010-25673662	x					x	x			x			Loss		Gain	Loss			Same			
2	chr19:25676337-25677056	x					x	x	x	x	x		Gain	Loss		Gain	Loss		Same	Same			
3	chr19:25682256-25683612	x		x	x	x	x	x	x	x	x	Loss	Gain	Loss	Loss	Gain	Same	Loss	Same	Same		x	hs112
4	chr19:25684275-25686162											Loss	Gain	Loss	Loss	Gain	Same	Loss	Same	Same		x	Dmrt3 TSS
5	chr19:25698077-25700494												Gain			Gain			Same				Dmrt3 5'UTR
Ctrl2	chr19:25746293-25747747		x										Same	Same		Same	Same		Loss	Same			Dmrt2 TSS

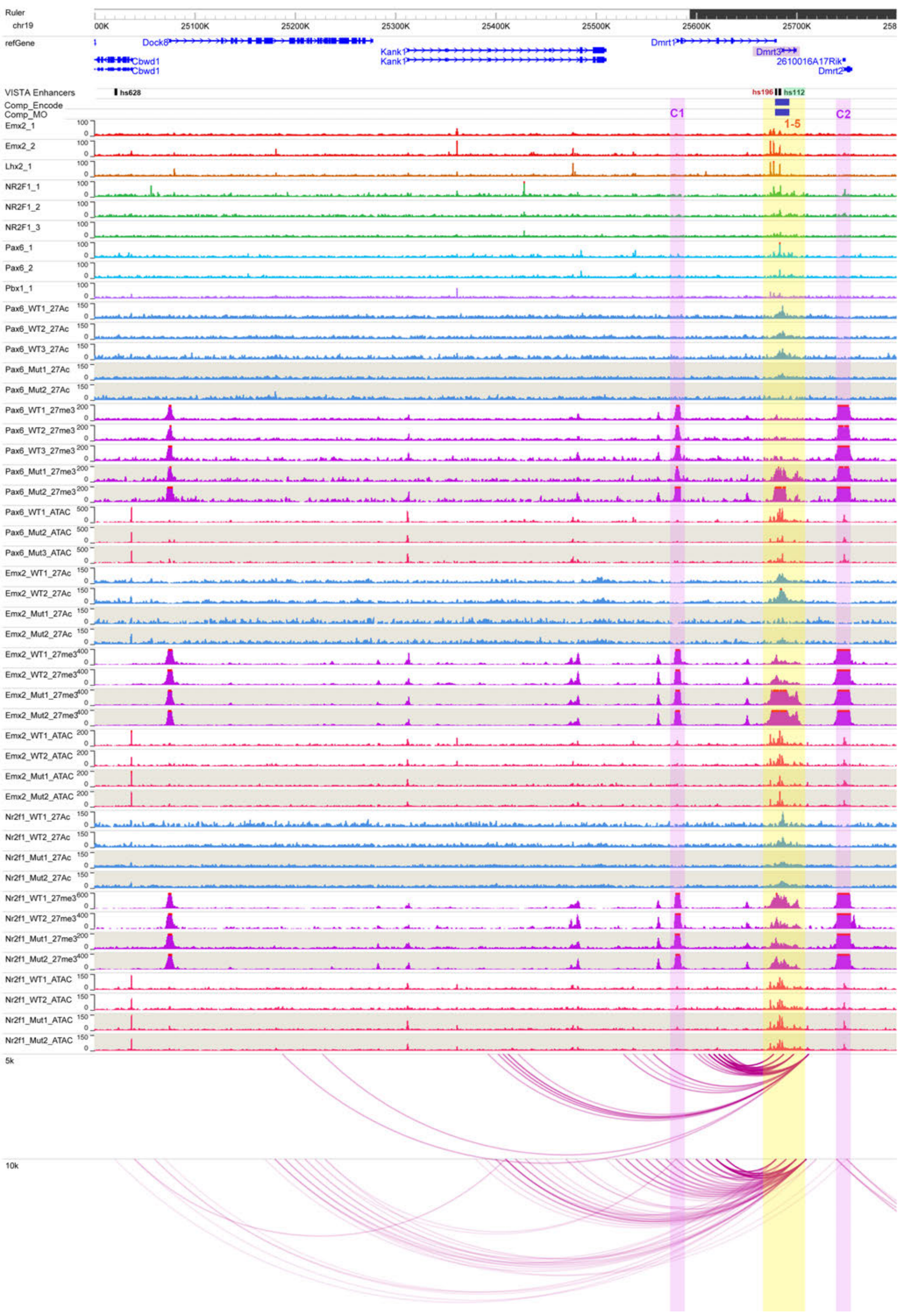

Dmrt5

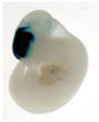

hs200

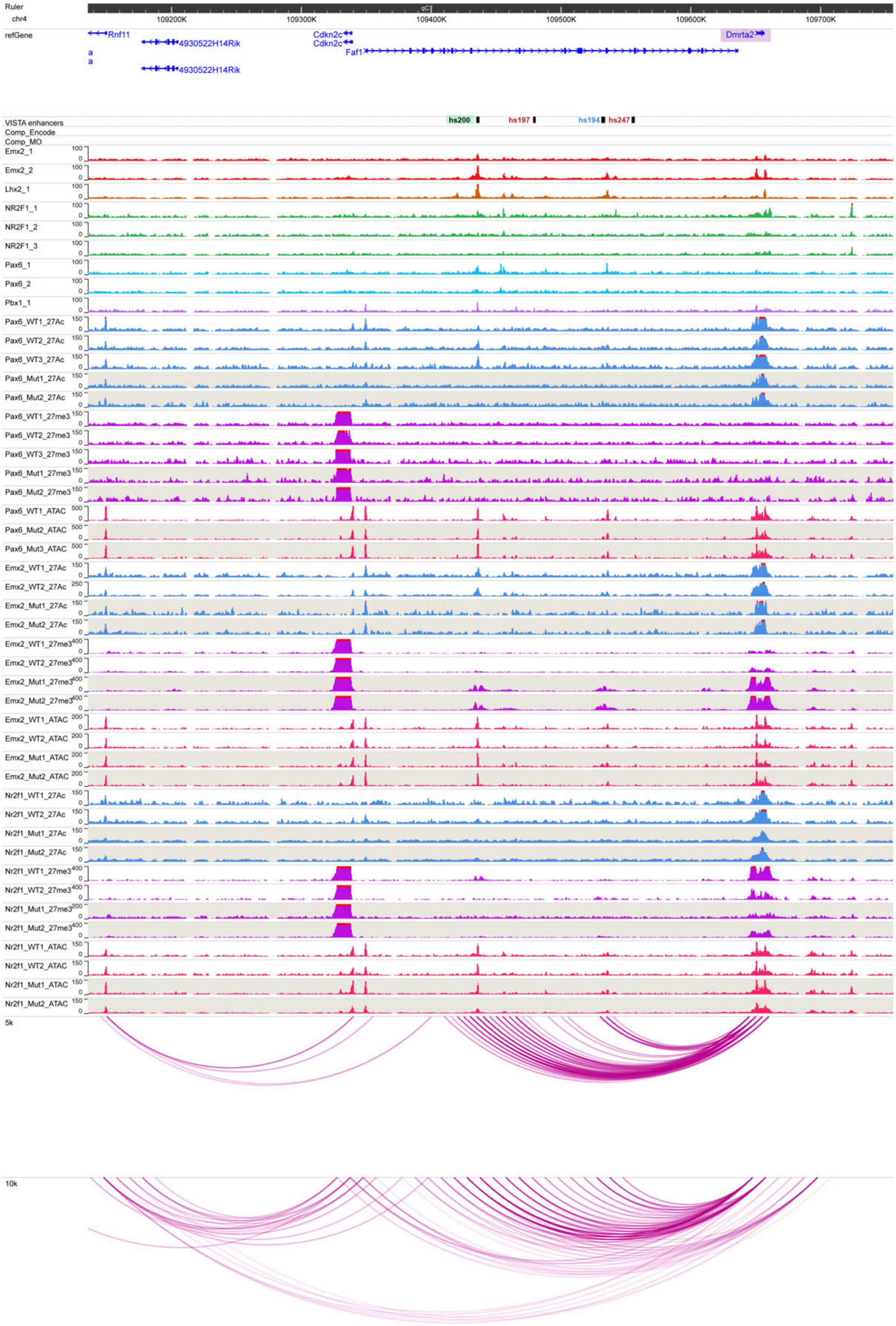

Ruler
chr4
109200K
109300K
109400K
109500K
109600K
109700K
refGene
Rnf11
4930522H14Rik
Cdkn2c
Cdkn2c
Faf1
Dmrta2
VISTA enhancers
hs200
hs197
hs194
hs247
Comp_Encode
Comp_MO
Emx2_1
Emx2_2
Lhx2_1
NR2F1_1
NR2F1_2
NR2F1_3
Pax6_1
Pax6_2
Pbx1_1
Pax6_WT1_27Ac
Pax6_WT2_27Ac
Pax6_WT3_27Ac
Pax6_Mut1_27Ac
Pax6_Mut2_27Ac
Pax6_WT1_27me3
Pax6_WT2_27me3
Pax6_WT3_27me3
Pax6_Mut1_27me3
Pax6_Mut2_27me3
Pax6_WT1_ATAC
Pax6_Mut2_ATAC
Pax6_Mut3_ATAC
Emx2_WT1_27Ac
Emx2_WT2_27Ac
Emx2_Mut1_27Ac
Emx2_Mut2_27Ac
Emx2_WT1_27me3
Emx2_WT2_27me3
Emx2_Mut1_27me3
Emx2_Mut2_27me3
Emx2_WT1_ATAC
Emx2_WT2_ATAC
Emx2_Mut1_ATAC
Emx2_Mut2_ATAC
Nr2f1_WT1_27Ac
Nr2f1_WT2_27Ac
Nr2f1_Mut1_27Ac
Nr2f1_Mut2_27Ac
Nr2f1_WT1_27me3
Nr2f1_WT2_27me3
Nr2f1_Mut1_27me3
Nr2f1_Mut2_27me3
Nr2f1_WT1_ATAC
Nr2f1_WT2_ATAC
Nr2f1_Mut1_ATAC
Nr2f1_Mut2_ATAC
5k
10k

Emx2

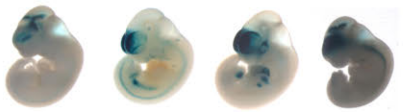

hs1032 hs1236 mm449 hs672

		TF ChIP Seq					Differential epigenomic marks in WT vs. Mutant									Plac-Seq	Computation	Genomic features
							Pax6 mutant			Emx2 mutant			Nr2f1 mutant					
		Emx2	Lhx2	Nr2f1	Pax6	Pbx1	27Ac	27me3	ATAC	27Ac	27me3	ATAC	27Ac	27me3	ATAC			
**Emx2**	**chr19:59400000-60526000**																	
1	chr19:59525206-59526676						Loss		Same	Loss		Same	Same		Same		x	
2	chr19:59531268-59535857	x	x	x		x	Loss		Same	Loss		Same	Same		Same		x	Emx2 TSS
3	chr19:59536094-59538976	x		x			Loss		Same	Loss		Loss	Same		Same		x	Emx2 Gene Body
4	chr19:59539919-59541154	x	x	x	x		Loss		Same	Loss		Same	Same		Same		x	hs1032
5	chr19:59541331-59542154	x	x	x			Loss		Same	Loss		Same	Same		Same		x	hs935
6	chr19:59543685-59546274	x	x	x			Loss		Same	Loss	Gain	Same	Same		Same		x	hs1221
7	chr19:59773144-59774850	x	x	x	x	x			Loss			Same			Same	x		
8	chr19:59963467-59965820	x	x						Same			Loss			Same	x		
9	chr19:59986713-59988654	x	x	x		x	Loss		Same	Loss		Loss	Same		Same	x		Rab11fip2
10	chr19:60091231-60093643	x	x	x		x	Loss		Same	Loss		Same	Same		Same	x		mm449
11	chr19:60275728-60287674	x	x					Same	Same		Loss	Same		Same	Same	x		
12	chr19:60301681-60303387						Same		Same	Same		Same	Loss		Same	x		D19ertd737e TSS

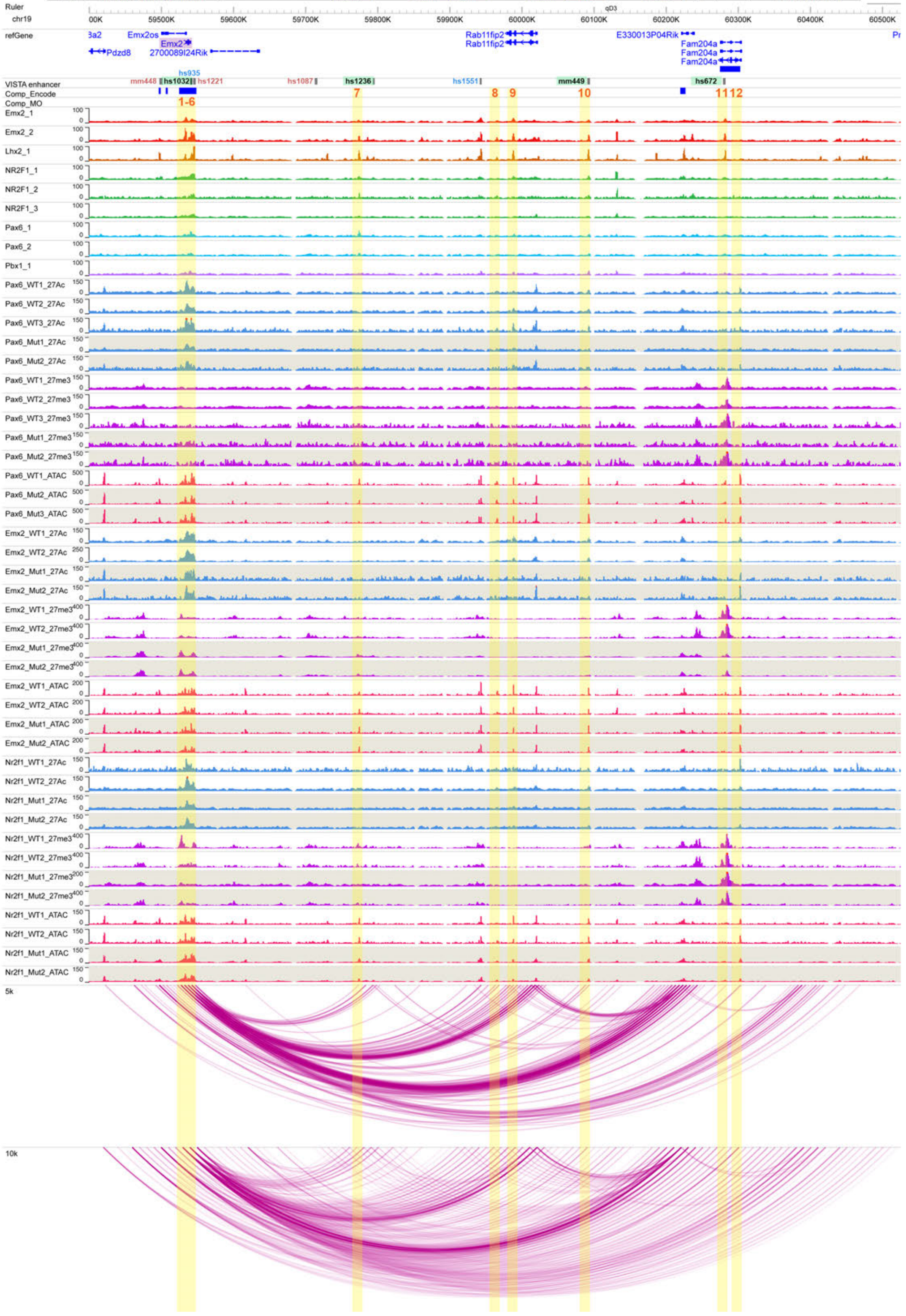

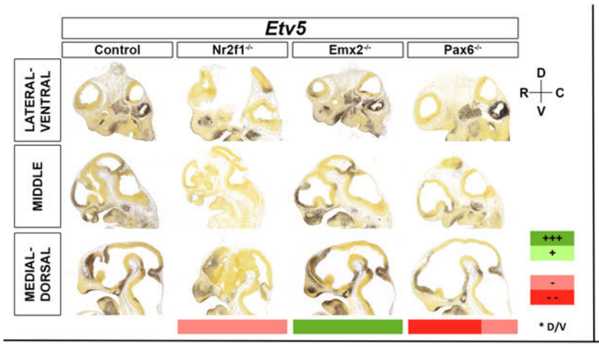

		Motifs					TF ChIP Seq					Differential epigenomic marks in WT vs. Mutant									Plac	Compu tation	Genomic features
												Pax6 mutant			Emx2 mutant			Nr2f1 mutant					
		Emx2 HB	Nr2f1	Pax6 Hb	Pax6 P	Pbx1	Emx2	Lhx2	Nr2f1	Pax6	Pbx1	27Ac	27me3	ATAC	27Ac	27me3	ATAC	27Ac	27me3	ATAC			
Etv5	chr16:22275000-22830000																						
1	chr16:22427340-22431723					x	x	x			x		Same	Loss		Small Loss			Same			x	
2	chr16:22433515-22438593					x	x				x		Same	Same		Small Loss			Same				Etv5 TSS
3	chr16:22628211-22636846															Small Gain							
4	chr16:22796861-22801628									x				Same?		Gain	Gain			Same	x		

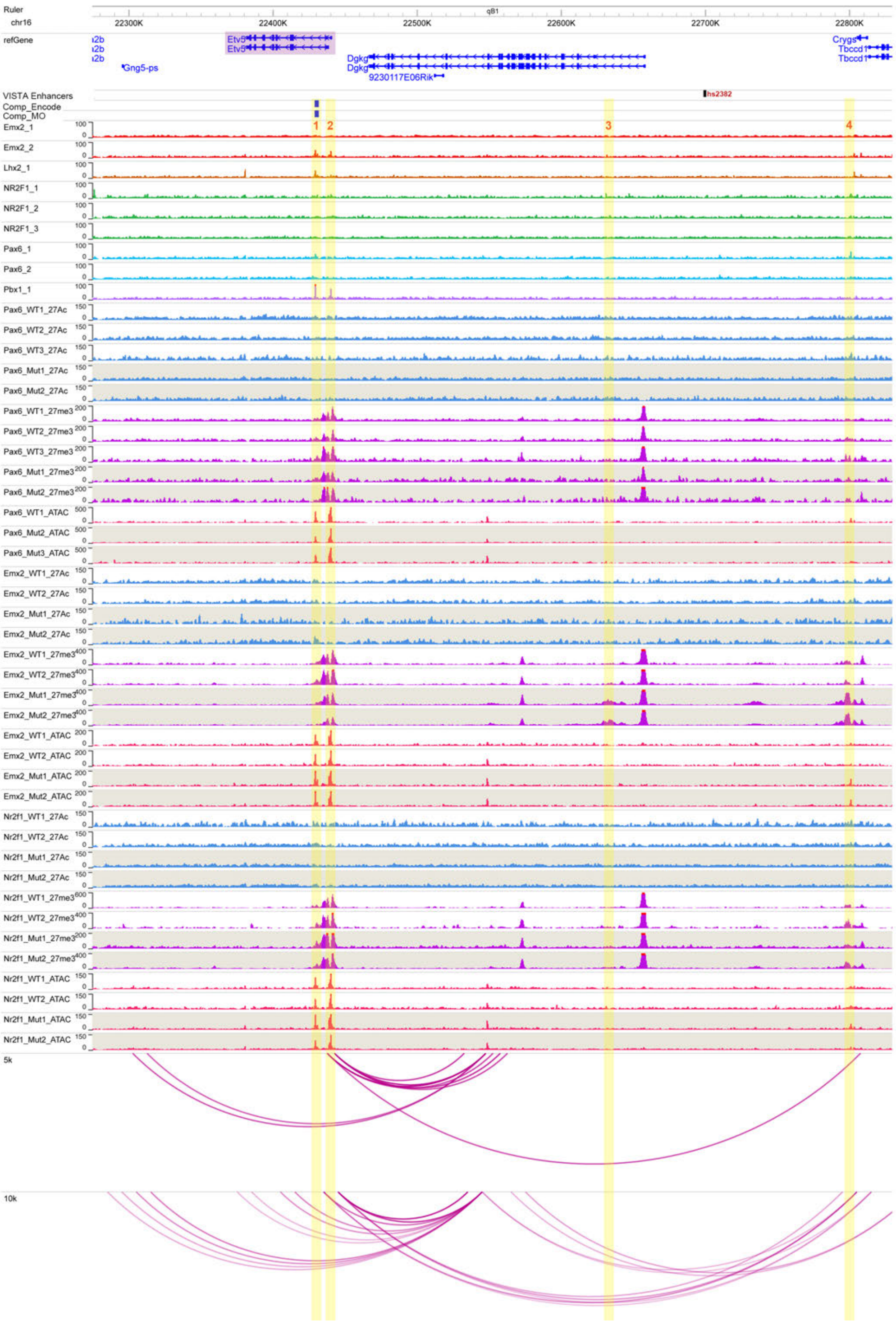

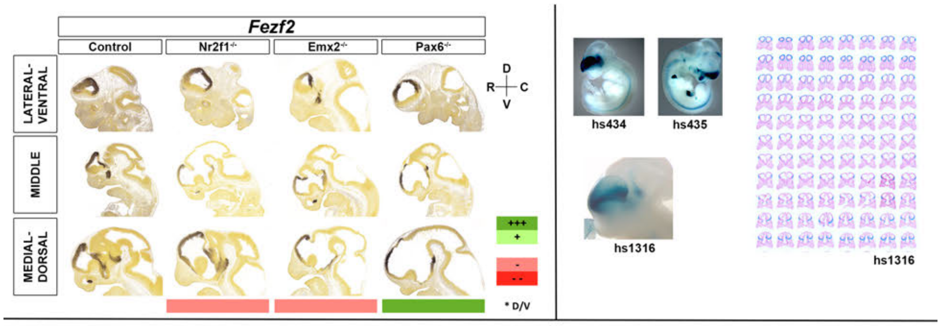

Fezf2	chr14:13000000-13370000	Motifs: Emx2 HB	Motifs: Nr2f1	Motifs: Pax6 HB	Motifs: Pax6 P	Motifs: Pbx1	TF ChIP Seq: Emx2	TF ChIP Seq: Lhx2	TF ChIP Seq: Nr2f1	TF ChIP Seq: Pax6	TF ChIP Seq: Pbx1	Differential epigenomic marks in WT vs. Mutant — Pax6 mutant: 27Ac	Pax6 mutant: 27me3	Pax6 mutant: ATAC	Emx2 mutant: 27Ac	Emx2 mutant: 27me3	Emx2 mutant: ATAC	Nr2f1 mutant: 27Ac	Nr2f1 mutant: 27me3	Nr2f1 mutant: ATAC	Plac	Computation	Genomic features
1	chr14:13021753-13022951	x		x	x		x	x			x	Loss		Same	Same		Same	Same		Same	x		
2	chr14:13169800-13171368	x					x	x	x	x	x	Loss		Same	Loss	Gain	Same	Loss		Same			hs434
3	chr14:13173323-13174387						x	x		x		Same		Same	Loss	Gain	Loss	Same		Same			
4	chr14:13177200-13178264						x	x		x		Loss		Loss	Loss	Gain	Same			Same			Fezf2 TSS
5	chr14:13178561-13179655						x	x				Same		Same	Loss	Gain	Same			Same			hs435
6	chr14:13180544-13181786	x		x	x	x	x	x	x	x				Loss		Gain	Loss			Same			
7	chr14:13214985-13216615												Same			Gain			Same		<10k		
8	chr14:13221010-13222735						x	x	x			Loss		Loss	Loss		Same	Same?		Same	x		

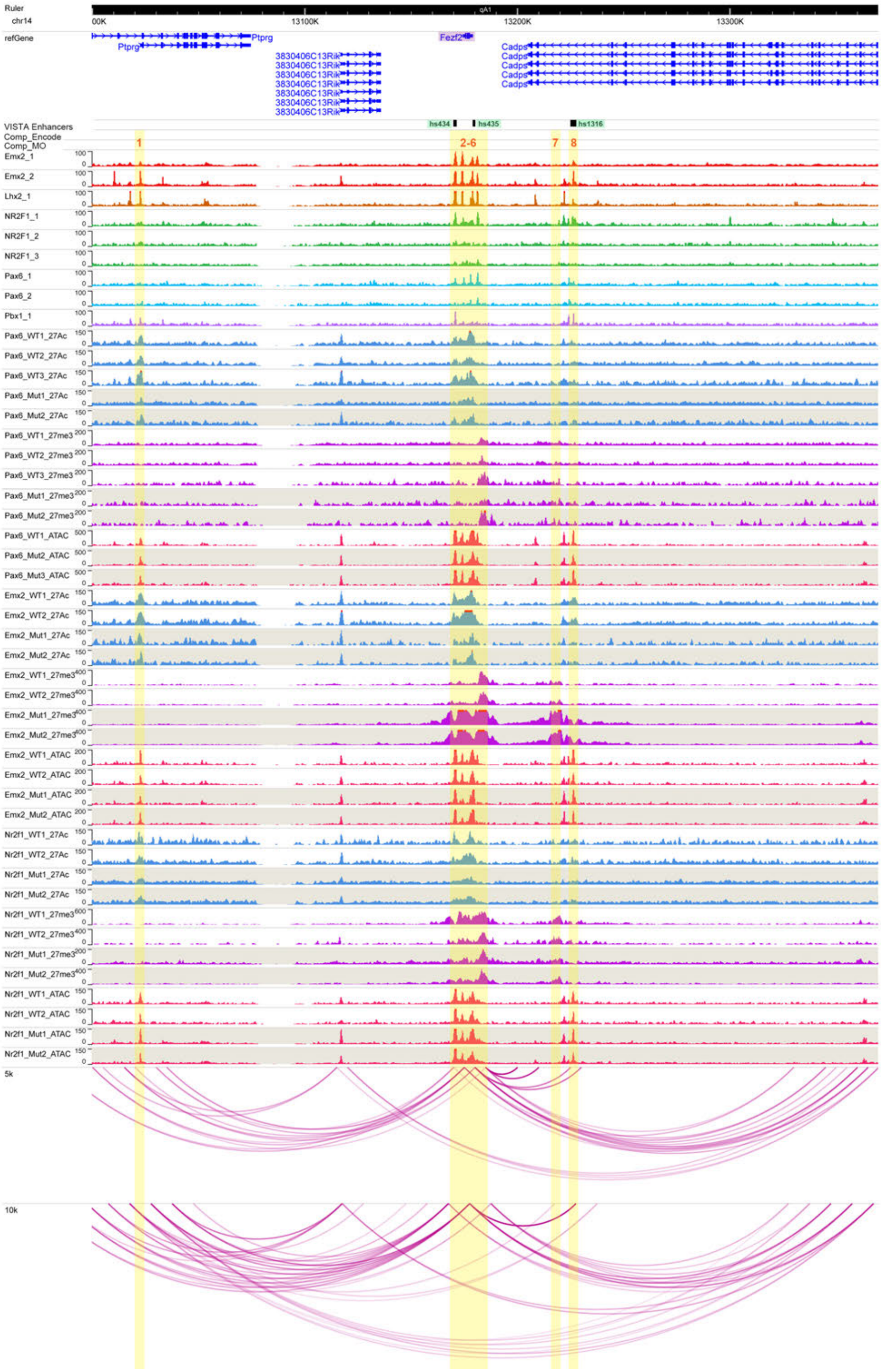

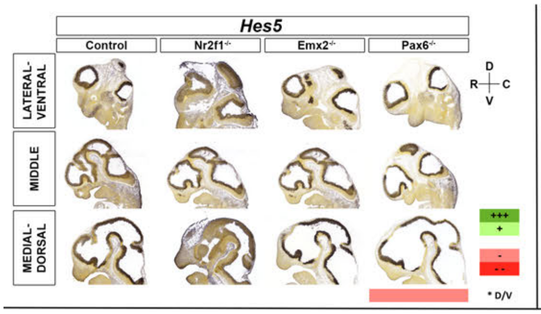

		Motifs					TF ChIP Seq					Differential epigenomic marks in WT vs. Mutant									Plac	Computation	Genomic features
												Pax6 mutant			Emx2 mutant			Nr2f1 mutant					
		Emx2 HB	Nr2f1	Pax6 Hb	Pax6 P	Pbx1	Emx2	Lhx2	Nr2f1	Pax6	Pbx1	27Ac	27me3	ATAC	27Ac	27me3	ATAC	27Ac	27me3	ATAC			
Hes5	chr4:154230000-154650000																						
1	chr4:154322202-154323976								x			Loss		Same			Same	Loss		Same		x	
2	chr4:154326550-154327836						x		x	x		Loss		Loss	Same		Same	Same		Same			
3	chr4:154334677-154335804	x					x	x				Same		Same	Small Gain?		Same			Same		x	Hes5 Gene Body
4	chr4:154607823-154610352								x			Same		Loss	Same		Same	Same		Same	x		mm318

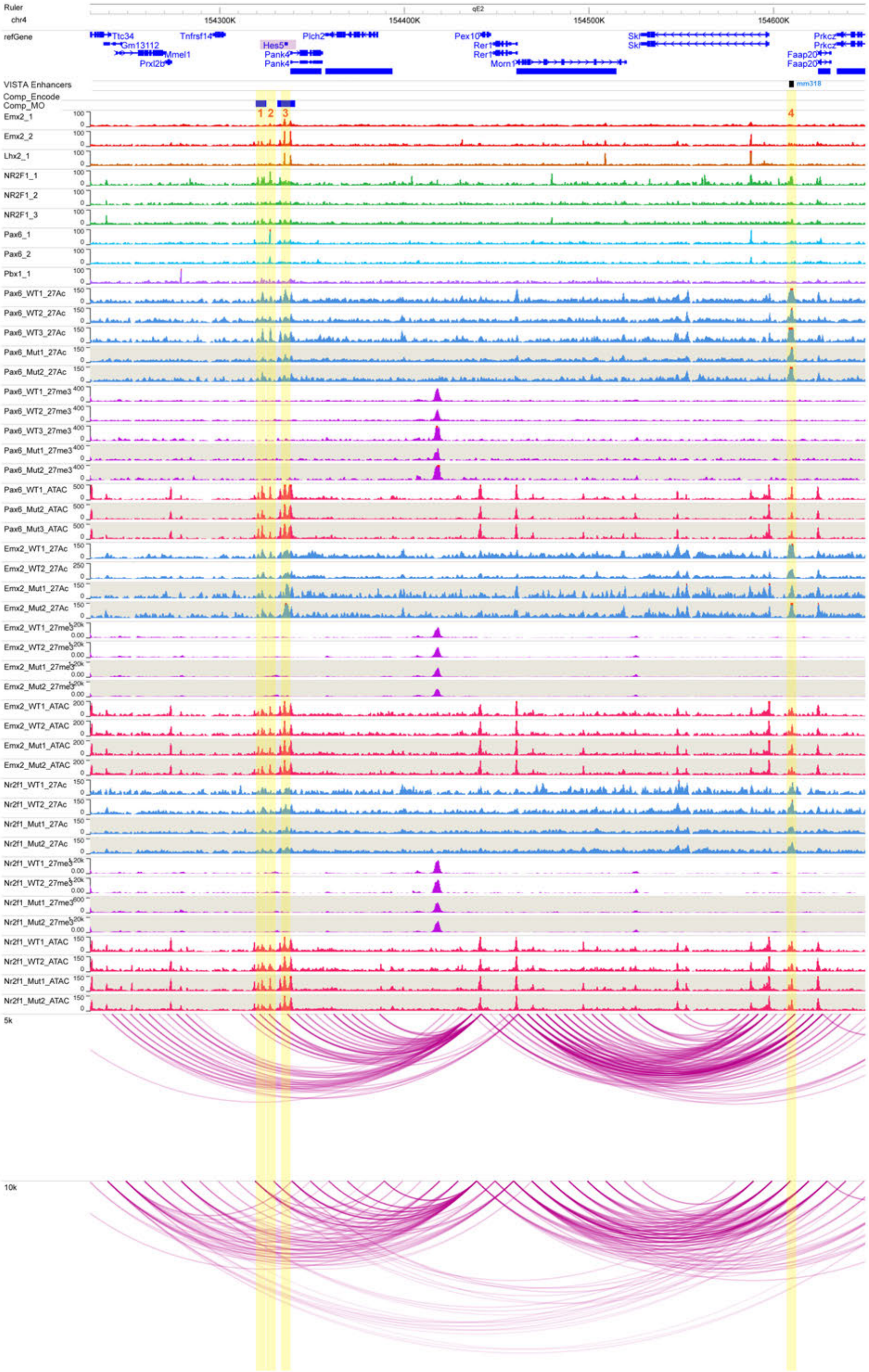

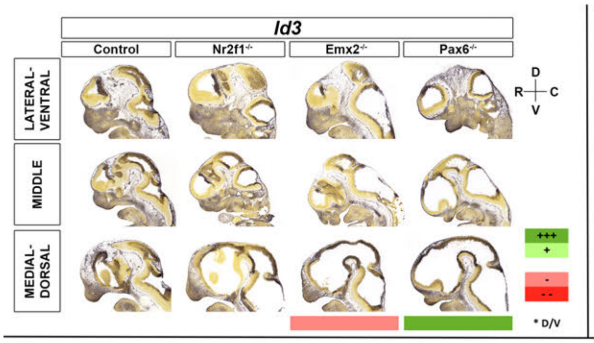

		Motifs					TF ChIP Seq					Differential epigenomic marks in WT vs. Mutant									Plac	Computation	Genomic features
												Pax6 mutant			Emx2 mutant			Nr2f1 mutant					
		Emx2 Hb	Nr2f1	Pax6 Hb	Pax6 P	Pbx1	Emx2	Lhx2	Nr2f1	Pax6	Pbx1	27Ac	27me3	ATAC	27Ac	27me3	ATAC	27Ac	27me3	ATAC			
Id3	chr4:135590000-135810000																						
1	chr4:135608849-135609824								x			Same		Same	Same		Same	Loss		Same	x		Rpl11 TSS
2	chr4:135793403-135795370												Loss			Same			Same			x	

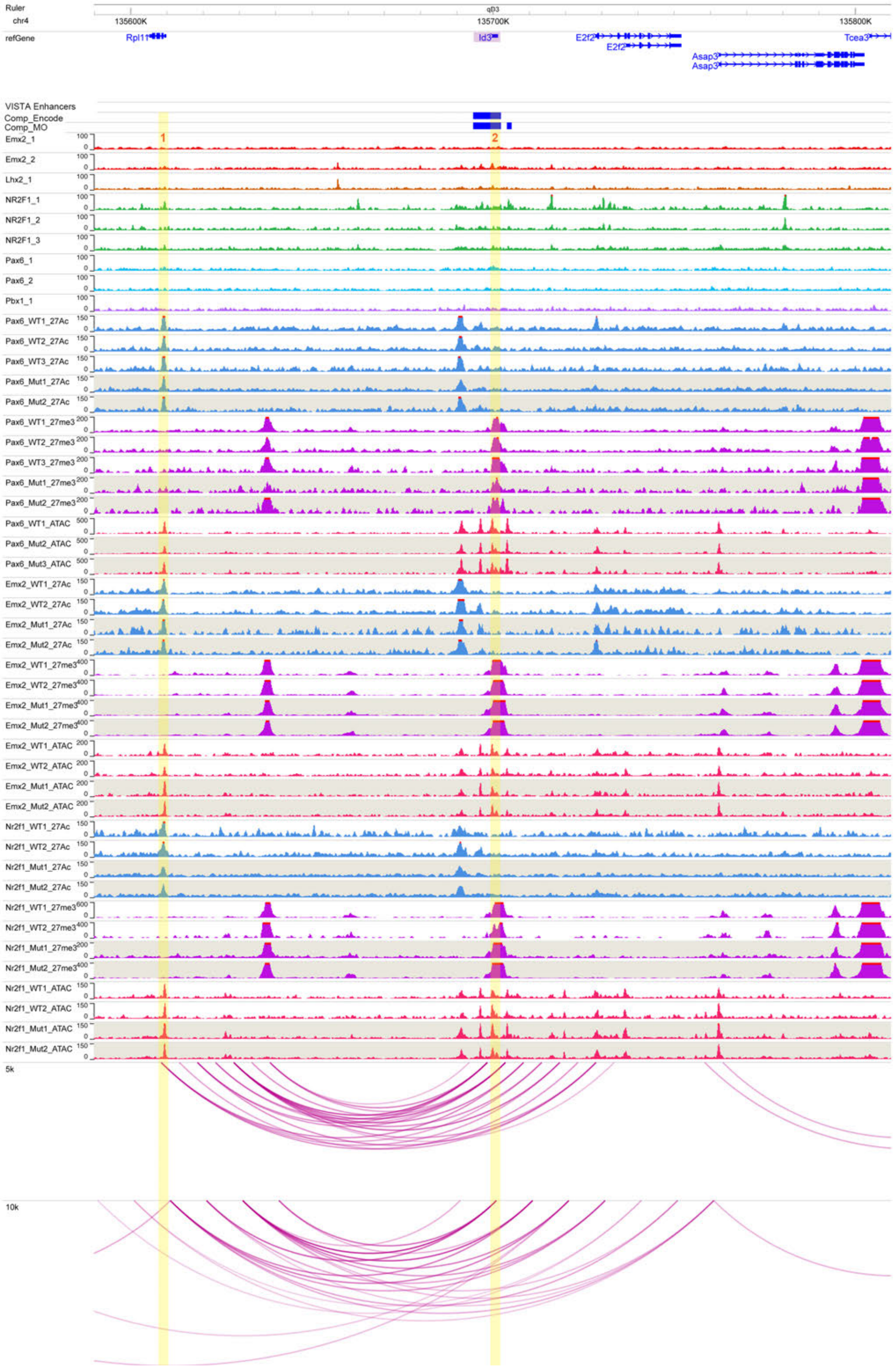

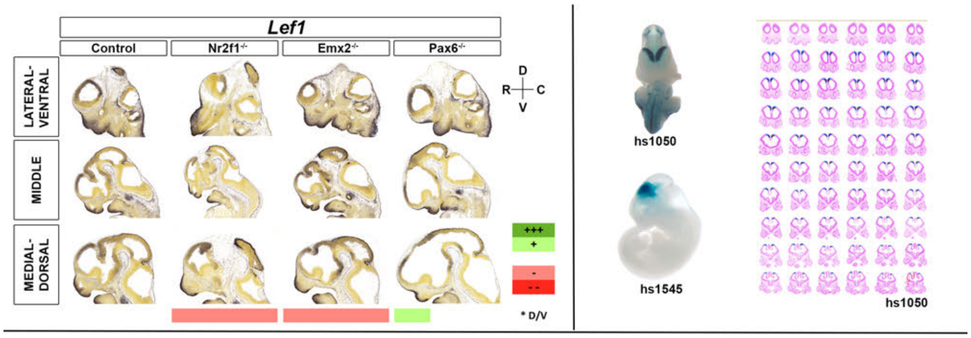

		Motifs					TF ChIP Seq					Differential epigenomic marks in WT vs. Mutant									Plac	Computation	Genomic features
												Pax6 mutant			Emx2 mutant			Nr2f1 mutant					
		Emx2 HB	Nr2f1	Pax6 Hb	Pax6 P	Pbx1	Emx2	Lhx2	Nr2f1	Pax6	Pbx1	27Ac	27me3	ATAC	27Ac	27me3	ATAC	27Ac	27me3	ATAC			
Lef1	chr3:130280000-131120000																						
1	chr3:130664999-130667081	x		x	x	x	x		x	x		Loss		Loss	Same		Same	Same		Same	x		hs1545
2	chr3:130774287-130776523						x	x	x	x				Same			Loss			Same			
3	chr3:130786473-130789557												Loss	Same		Same	Same		Same	Same			
4	chr3:130809043-130812870												Same	Same		Gain	Same		Same	Same			Lef1 Promoter
5	chr3:131035570-131040367	x					x	x		x	x		Same	Same		Loss	Loss		Same	Same			Cyp2u1

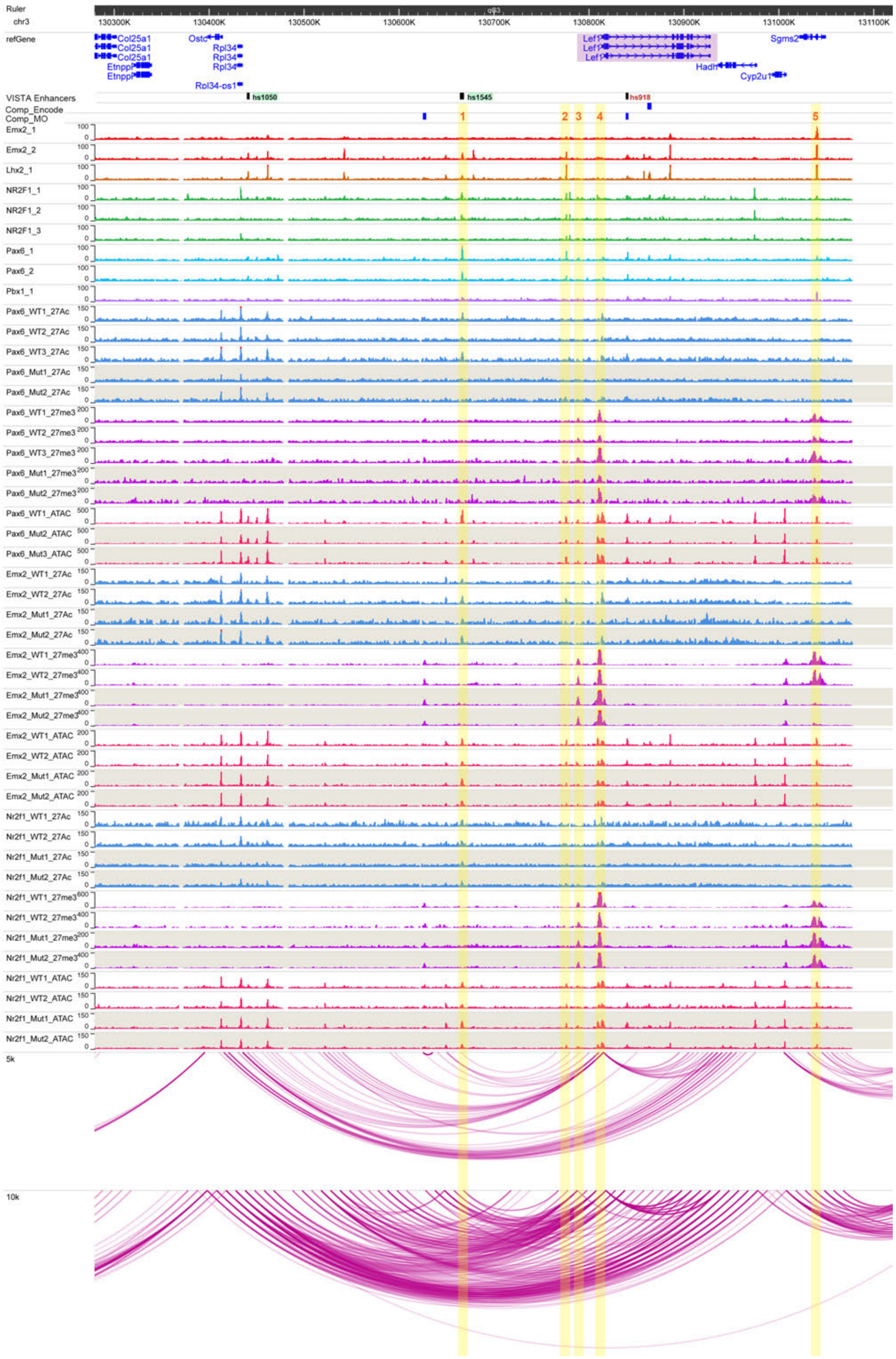

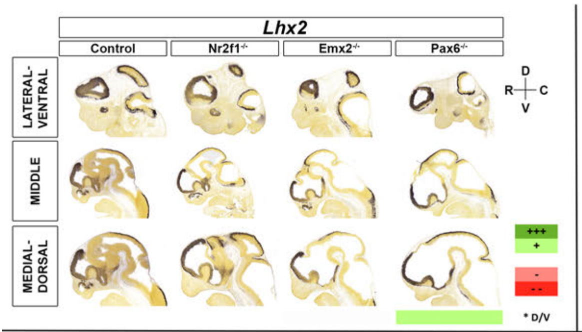

		Motifs					TF ChIP Seq					Differential epigenomic marks in WT vs. Mutant									Plac	Computation	Genomic features
												Pax6 mutant			Emx2 mutant			Nr2f1 mutant					
		Emx2 Hb	Nr2f1	Pax6 Hb	Pax6 P	Pbx1	Emx2	Lhx2	Nr2f1	Pax6	Pbx1	27Ac	27me3	ATAC	27Ac	27me3	ATAC	27Ac	27me3	ATAC			
Lhx2	chr2:37560000-38600000																						
1	chr2:37997193-38001351	x				x	x		x		x	Loss		Same	Loss		Same	Same		Same			hs314
2	chr2:38048398-38056606						x	x	x					Same		Gain	Same			Same			
3	chr2:38194392-38197269	x					x	x	x		x	Loss		Same	Loss	Gain	Same	Loss		Same			Lhx2 TSS
4	chr2:38202560-38207368	x				x	x	x	x		x	Loss		Same	Loss	Gain	Same	Loss		Same			Lhx2 alternative TSS
5	chr2:38370512-38372007								x		x	Loss		Loss	Loss		Same			Same			Nek6 TSS

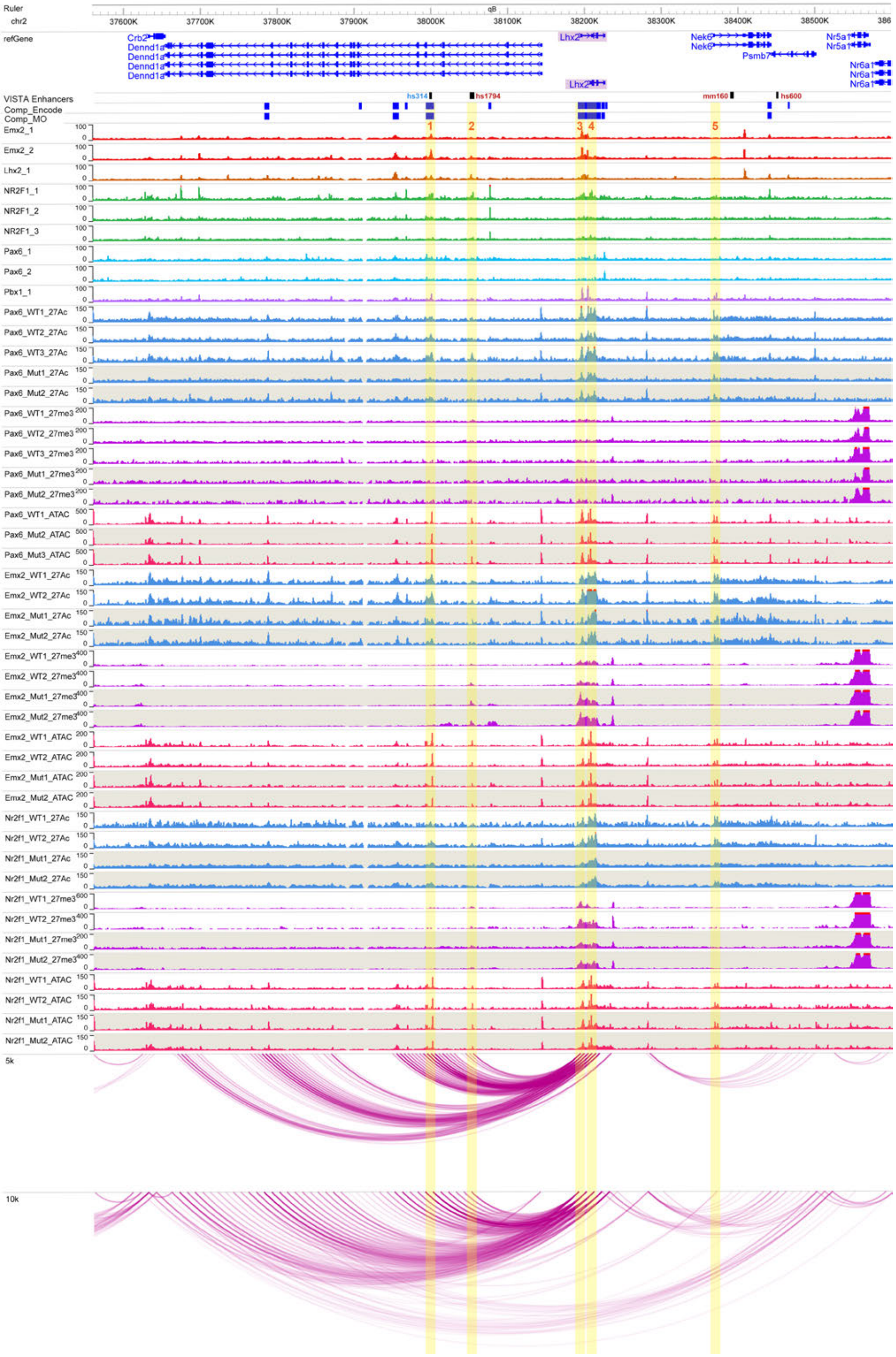

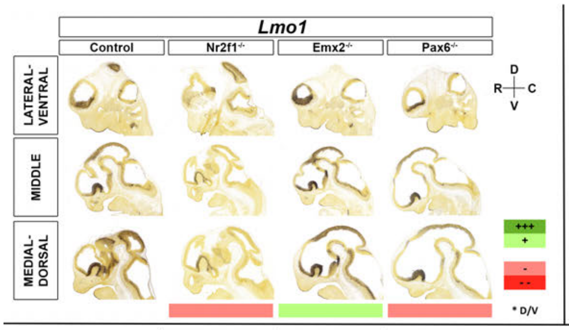

		Motifs					TF ChIP Seq					Differential epigenomic marks in WT vs. Mutant									Plac	Computation	Genomic features
												Pax6 mutant			Emx2 mutant			Nr2f1 mutant					
		Emx2 HB	Nr2f1	Pax6 Hb	Pax6 P	Pbx1	Emx2	Lhx2	Nr2f1	Pax6	Pbx1	27Ac	27me3	ATAC	27Ac	27me3	ATAC	27Ac	27me3	ATAC			
Lmo1	chr7:116182079-116670000																						
1	chr7:116254369-116260805						x	x	x	x			Same			Loss			Same				hs1859
2	chr7:116269340-116274412	x					x	x					Same			Loss			Same				
3	chr7:116285437-116289362	x					x	x	x				Same	Loss		Loss	Loss		Same				Lmo1 Gene Body
4	chr7:116307961-116310857	x					x	x		x			Same	Loss		Loss	Same		Same	Same			Lmo1 Gene Body
5	chr7:116310160-116318282						x						Same			Loss			Same				Lmo1 TSS
6	chr7:116318810-116326219	x		x	x		x	x	x	x	x	Loss	Same	Same	Gain	Loss	Same	Loss	Same	Same		x	
7	chr7:116333909-116336398	x					x	x	x	x	x	Loss	Same	Same	Same	Loss	Same	Loss	Same	Same			
8	chr7:116344968-116348740												Same			Loss			Same				
9	chr7:116581666-116584183												Same		Gain	Loss			Same		x		

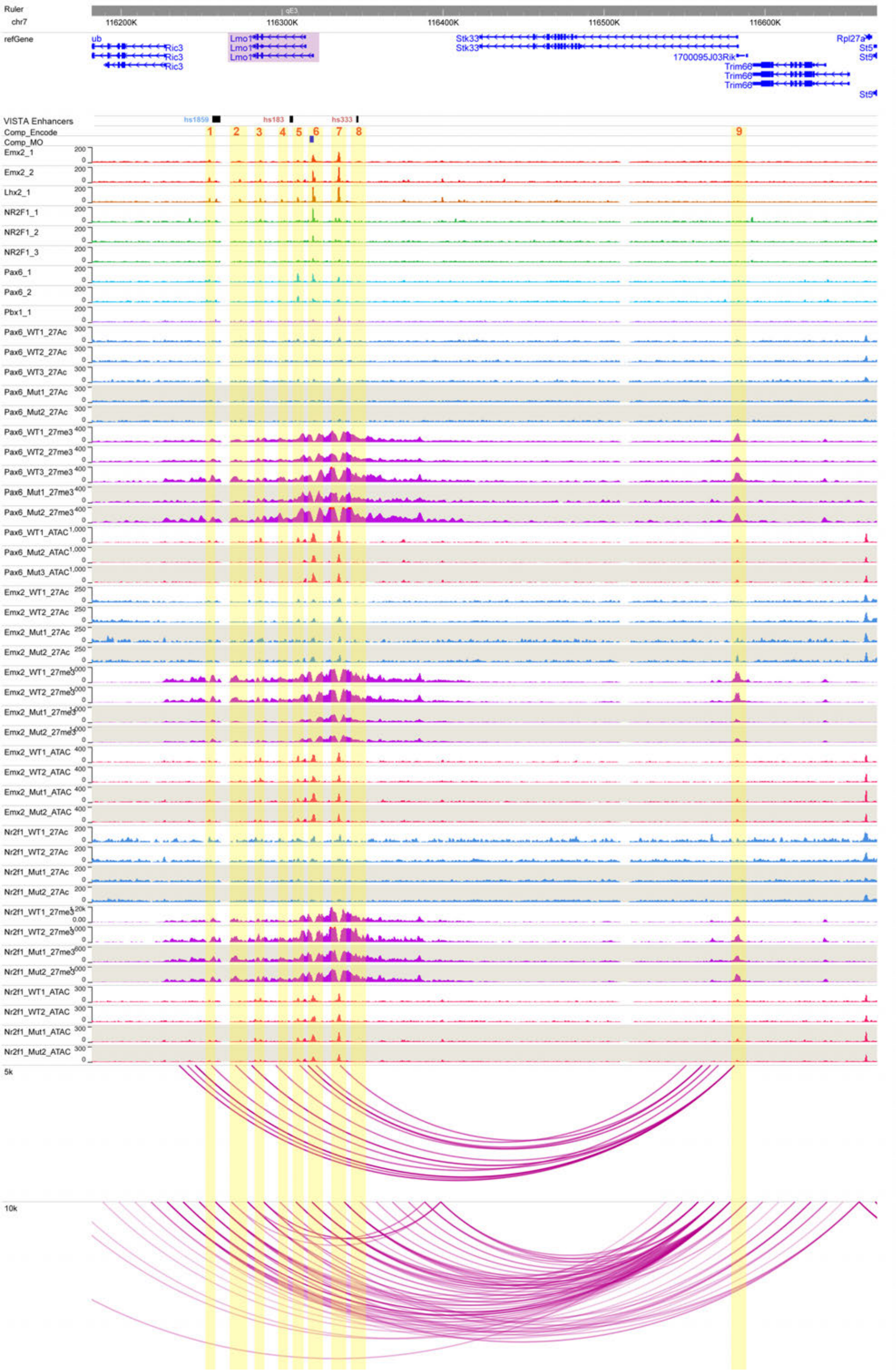

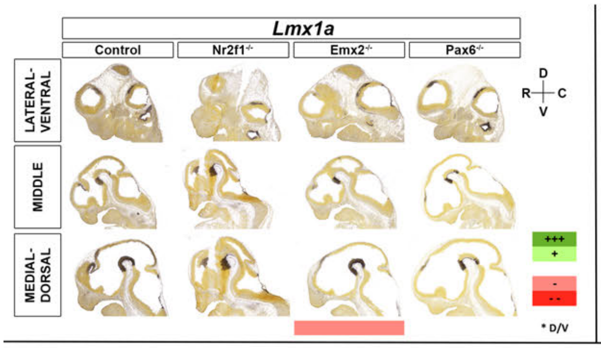

		Motifs					TF ChIP Seq					Differential epigenomic marks in WT vs. Mutant									Plac	Computation	Genomic features
												Pax6 mutant			Emx2 mutant			Nr2f1 mutant					
		Emx2 HB	Nr2f1	Pax6 Hb	Pax6 P	Pbx1	Emx2	Lhx2	Nr2f1	Pax6	Pbx1	27Ac	27me3	ATAC	27Ac	27me3	ATAC	27Ac	27me3	ATAC			
Lmx1a	chr1:169280000-169880000																						
1	chr1:169527243-169530622												Same			Loss			Same				
2	chr1:169589344-169592457												Same			Gain			Loss				
3	chr1:169601564-169604678												Same			Same			Loss				
4	chr1:169617808-169623158								x				Same	Small Loss		Same	Small loss		Same	Same			
5	chr1:169731979-169733692			x	x		x	x				Loss		Same	Same		Same	Same		Same			

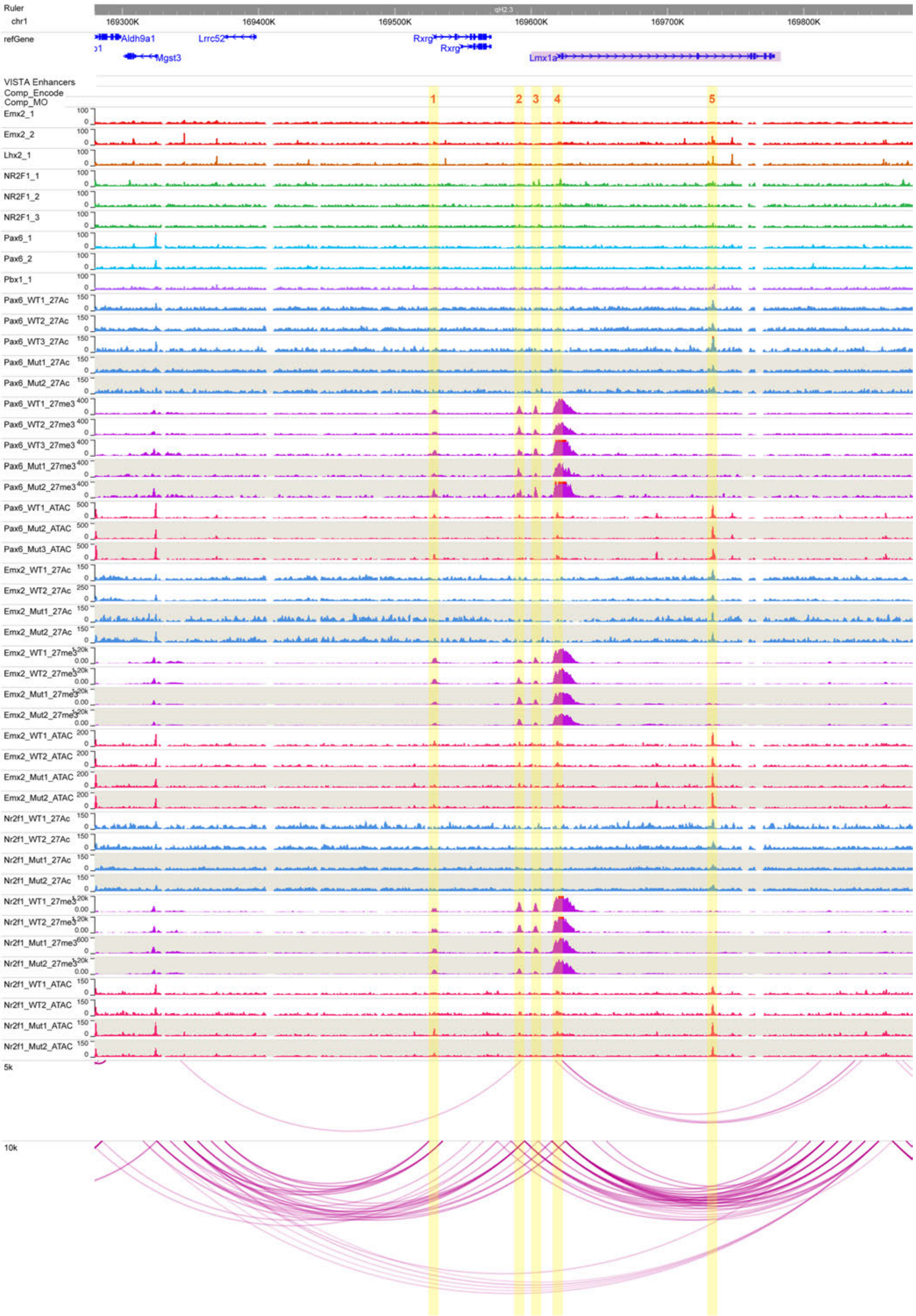

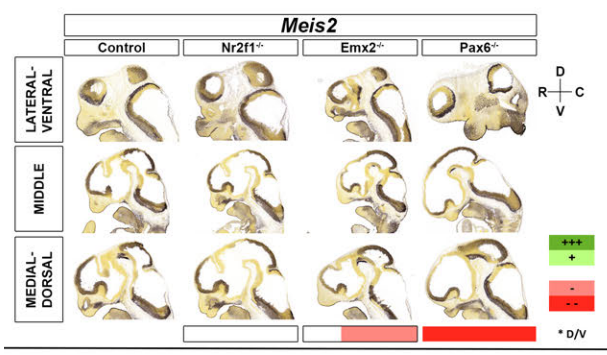

		Motifs					TF ChIP Seq					Differential epigenomic marks in WT vs. Mutant									Plac-Seq	Computation	Genomic features
												Pax6 mutant			Emx2 mutant			Nr2f1 mutant					
		Emx2 HB	Nr2f1	Pax6 Hb	Pax6 P	Pbx1	Emx2	Lhx2	Nr2f1	Pax6	Pbx1	27Ac	27me3	ATAC	27Ac	27me3	ATAC	27Ac	27me3	ATAC			
Meis2	chr2:114600000-116700000																						
1	chr2:114957467-114958808	x		x	x	x	x	x	x	x	x	Loss		Same	Loss		Same	Loss		Same	x		
2	chr2:115144553-115152886												Small gain			Gain			Same				
3	chr2:115281249-115282302	x					x	x	x	x	x	Loss		Same	Loss		Loss	Same		Same	x		
4	chr2:115293609-115294718	x		x	x		x	x		x		Same		Loss	Loss		Loss	Same		Same	x		
5	chr2:115482362-115483493	x					x	x	x		x	Same		Same	Loss		Loss	Same		Same	x		hs812
6	chr2:115485397-115487093	x					x	x	x	x	x	Loss		Same	Loss		Loss	Loss		Same	x	x	
7	chr2:115550455-115552059	x		x	x		x	x	x		x	Loss		Same	Loss		Loss	Same		Same	x	x	
8	chr2:115677352-115681367												Gain			Gain			Same		x	x	Meis2 5' UTR
9	chr2:115897214-115902068	x			x		x	x	x				Gain	Same		Gain	Same		Same	Same		x	Near Meis2 TSS

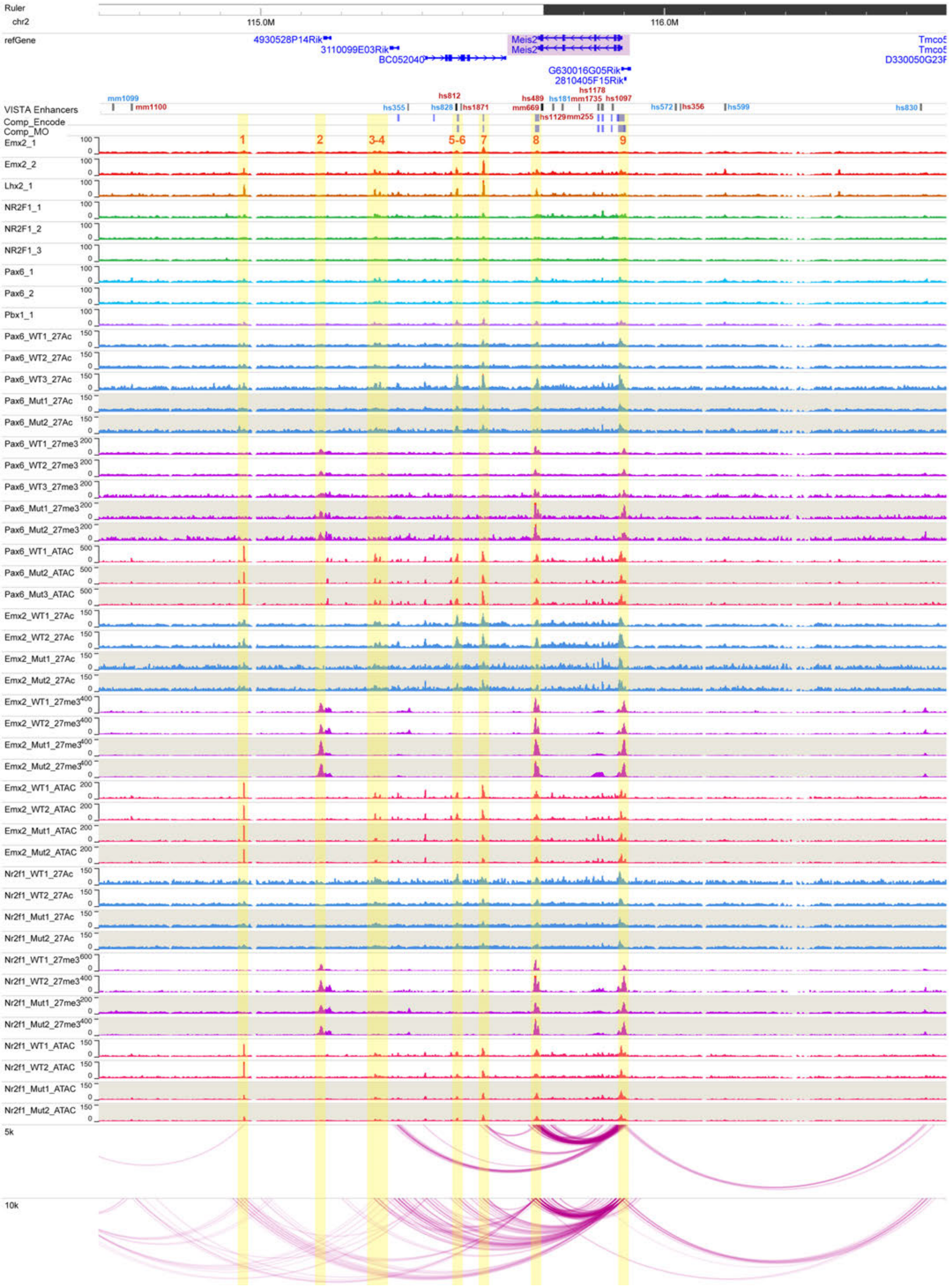

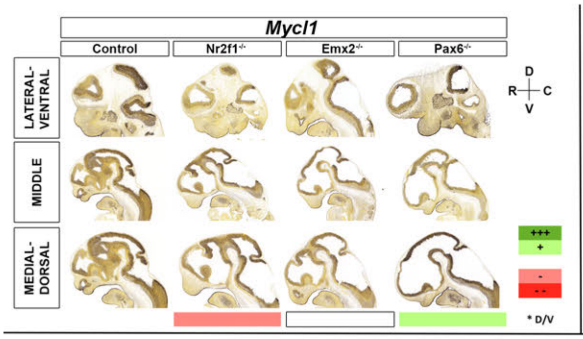

		Motifs					TF ChIP Seq					Differential epigenomic marks in WT vs. Mutant									Plac-Seq	Computation	Genomic features
												Pax6 mutant			Emx2 mutant			Nr2f1 mutant					
		Emx2 HB	Nr2f1	Pax6 Hb	Pax6 P	Pbx1	Emx2	Lhx2	Nr2f1	Pax6	Pbx1	27Ac	27me3	ATAC	27Ac	27me3	ATAC	27Ac	27me3	ATAC			
Mycl1	chr4:122560000-122800000																						
1	chr4:122672621-122674388											Loss		Same	Same		Same	Loss		Same		x	Mycl1 TSS
2	chr4:122690008-122692021											Loss		Same	Same		Same	Same		Same		x	
3	chr4:122692924-122694514											Loss		Same	Same		Same	Same		Same		x	Trit1 TSS
4	chr4:122727680-122729103								x			Same		Same	Same		Same	Loss		Same		x	Trit1 Gene body

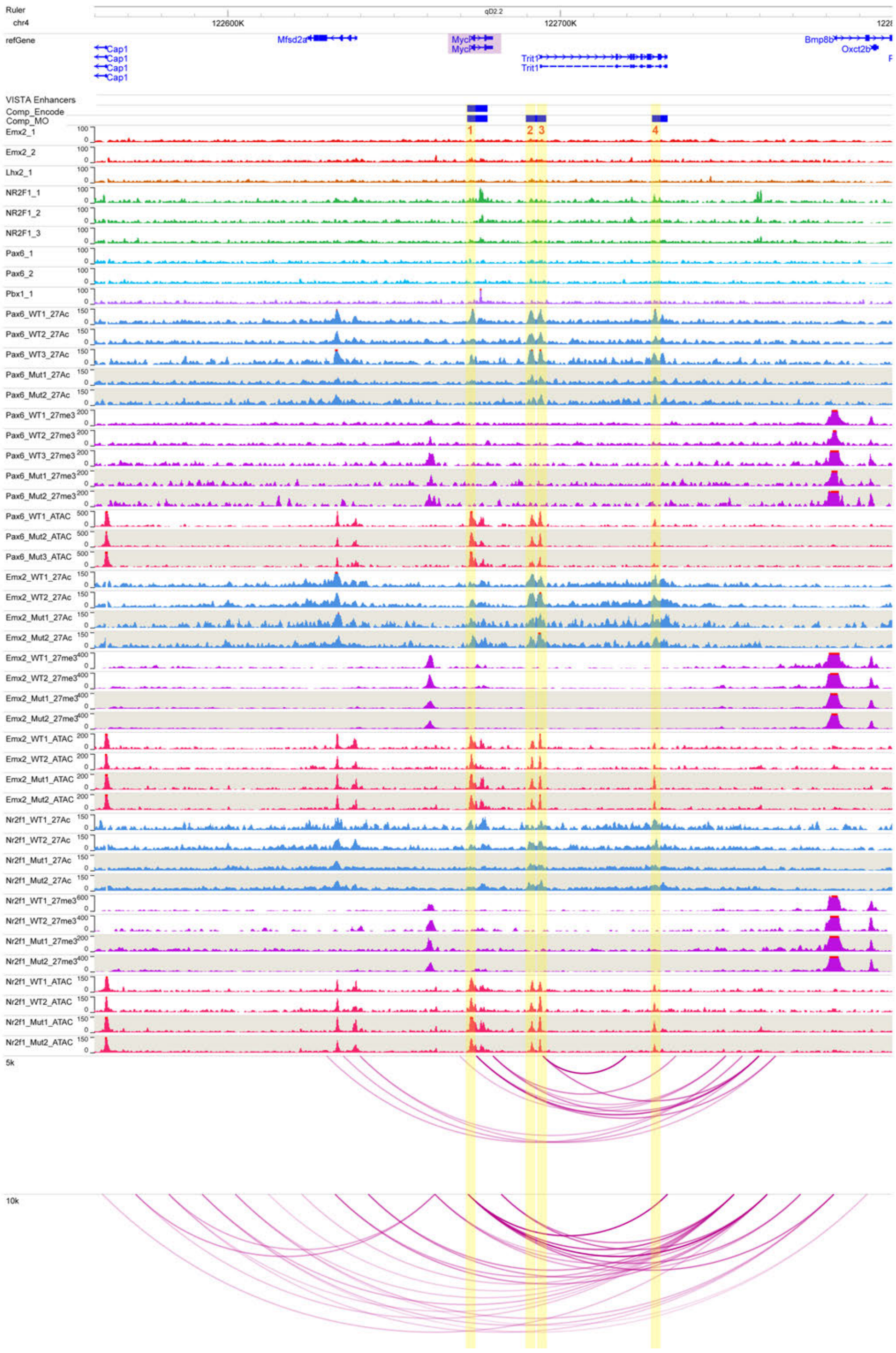

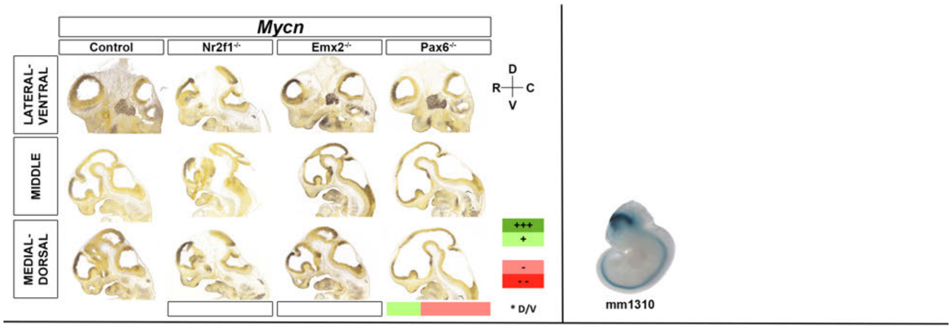

		Motifs					TF ChIP Seq					Differential epigenomic marks in WT vs. Mutant									Plac-Seq	Computation	Genomic features
												Pax6 mutant			Emx2 mutant			Nr2f1 mutant					
		Emx2 HB	Nr2f1	Pax6 Hb	Pax6 P	Pbx1	Emx2	Lhx2	Nr2f1	Pax6	Pbx1	27Ac	27me3	ATAC	27Ac	27me3	ATAC	27Ac	27me3	ATAC			
Mycn	chr12:12285000-13290000																						
1	chr12:12514739-12516576	x		x	x		x	x	x			Loss		Loss	Loss		Loss	Loss		Same	x		
2	chr12:12532797-12535809	x					x	x	x		x	Same		Same	Loss		Loss	Same		Same	x	x	
3	chr12:12587394-12589056						x		x		x	Same?		Same	Same		Same	Loss		Same	x	x	
4	chr12:12690102-12692253											Loss		Same	Loss		Same	Loss		Same	x		
5	chr12:12745097-12746653	x					x	x	x	x		Loss		Same	Loss		Same	Loss		Same	x	x	
6	chr12:12789957-12791513	x					x	x	x			Same		Same	Loss		Same	Same		Same	x		
7	chr12:12910817-12912373								x			Loss		Same	Same		Same	Same		Same			
8	chr12:12943665-12946290	x					x		x			Loss		Same	Same		Same	Loss		Same		x	Mycn Gene body
9	chr12:12948261-12950492						x		x			Loss		Same	Same		Same	Loss		Same		x	Mycn Gene body
10	chr12:13017470-13019026	x					x	x			x			Loss			Loss			Same			
11	chr12:13215205-13216274								x	x				Loss			Same			Same	x		

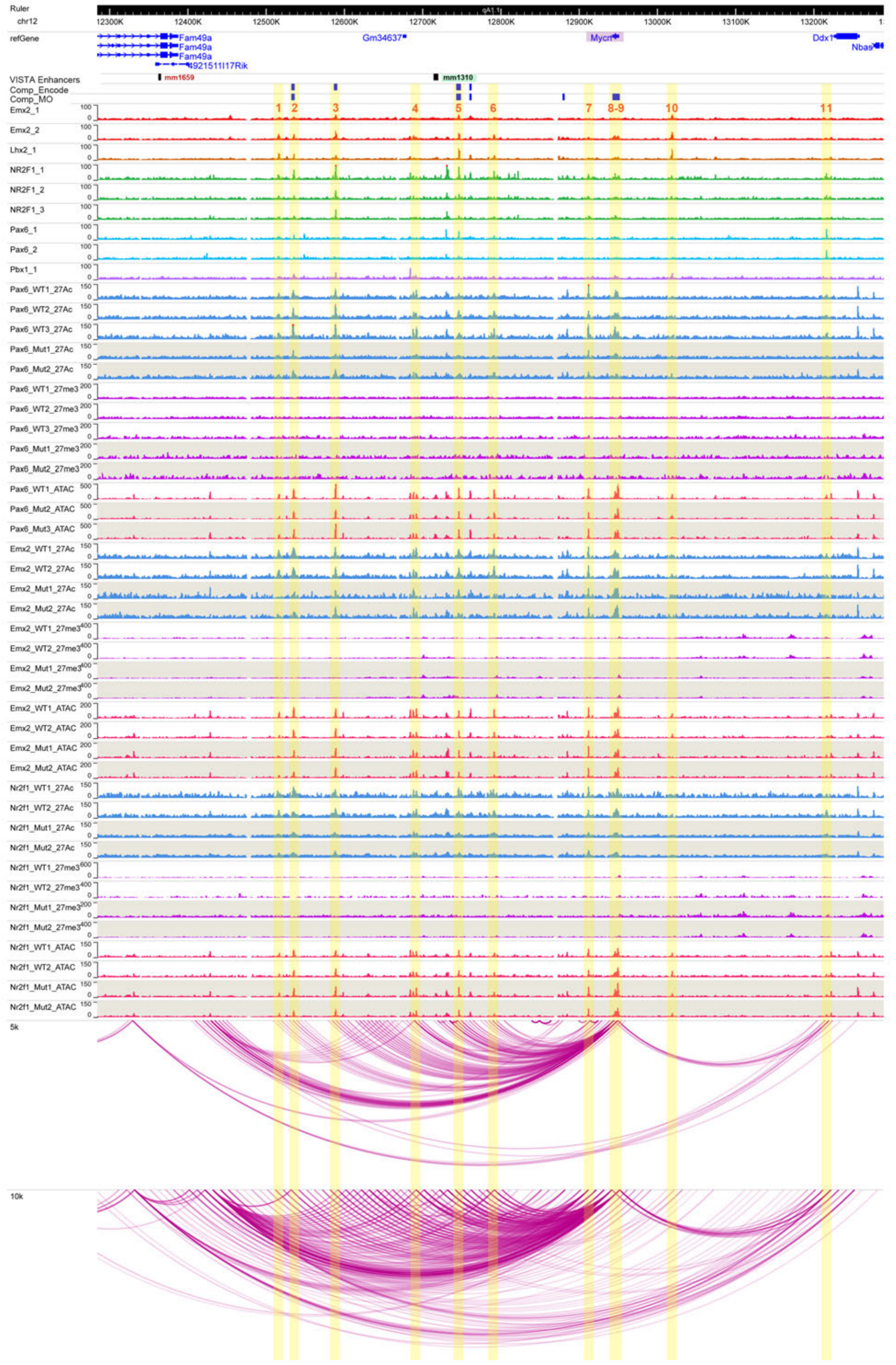

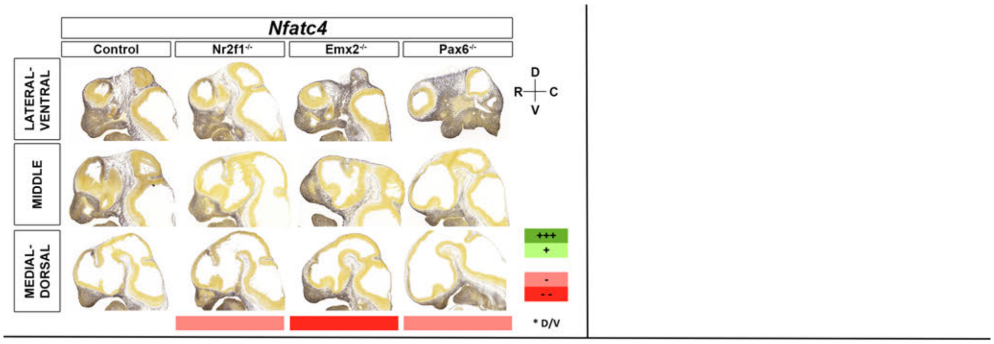

		Motifs					TF ChIP Seq					Differential epigenomic marks in WT vs. Mutant									Plac-Seq	Computation	Genomic features
												Pax6 mutant			Emx2 mutant			Nr2f1 mutant					
		Emx2 HB	Nr2f1	Pax6 Hb	Pax6 P	Pbx1	Emx2	Lhx2	Nr2f1	Pax6	Pbx1	27Ac	27me3	ATAC	27Ac	27me3	ATAC	27Ac	27me3	ATAC			
Nfatc4	chr14:56343632-56560000																						
1	chr14:56440792-56446602								x							Gain							Nfatc4 TSS

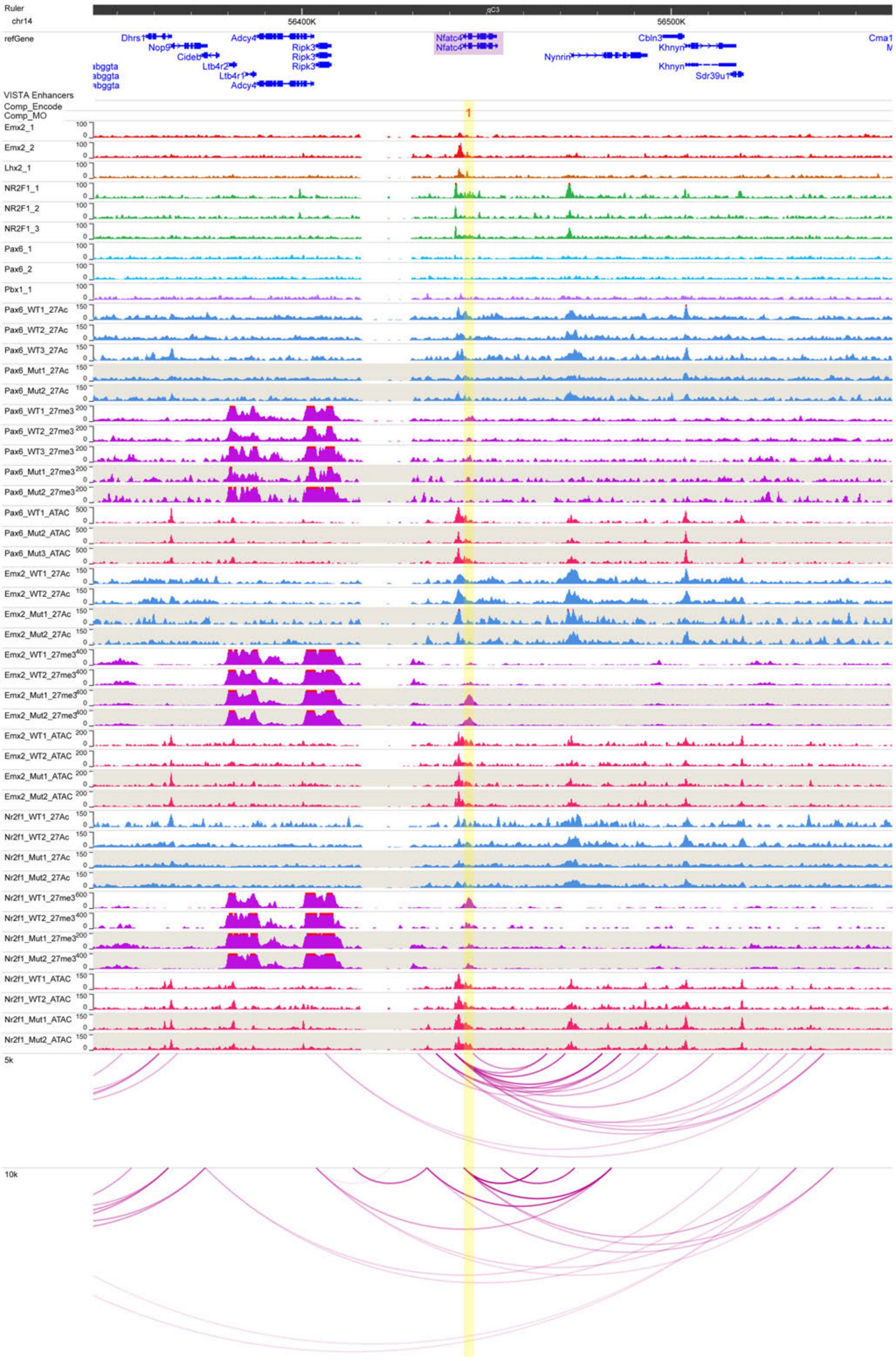

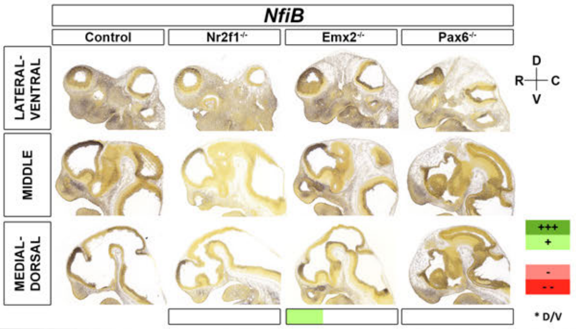

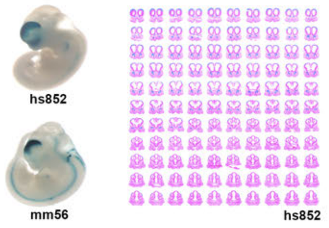

		Motifs					TF ChIP Seq					Differential epigenomic marks in WT vs. Mutant									Plac-Seq	Computation	Genomic features
												Pax6 mutant			Emx2 mutant			Nr2f1 mutant					
		Emx2 HB	Nr2f1	Pax6 Hb	Pax6 P	Pbx1	Emx2	Lhx2	Nr2f1	Pax6	Pbx1	27Ac	27me3	ATAC	27Ac	27me3	ATAC	27Ac	27me3	ATAC			
**NfiB**	chr4:81100000-82900000																						
**1**	chr4:81270987-81272556						x	x	x					Same			Loss			Same	x		
**2**	chr4:81673006-81674575	x					x	x			x			Same			Gain			Same			
**3**	chr4:81949394-81952730			x			x		x	x		Loss		Same	Same		Same	Same		Same	x		
**4**	chr4:82029483-82031642	x					x					Same		Same	Loss		Loss	Same		Same		x	
**5**	chr4:82150505-82154090	x		x			x	x	x	x	x	Loss		Same	Gain		Same	Same		Same		x	Nfib TSS
**6**	chr4:82181026-82186913	x					x		x		x		Same			Gain			Loss			x	
**7**	chr4:82525419-82535633	x					x	x								Gain							Cer1 Gene Body
**8**	chr4:82561255-82563610	x					x	x			x	Same		Same	Loss		Loss	Same		Same	x		mm56

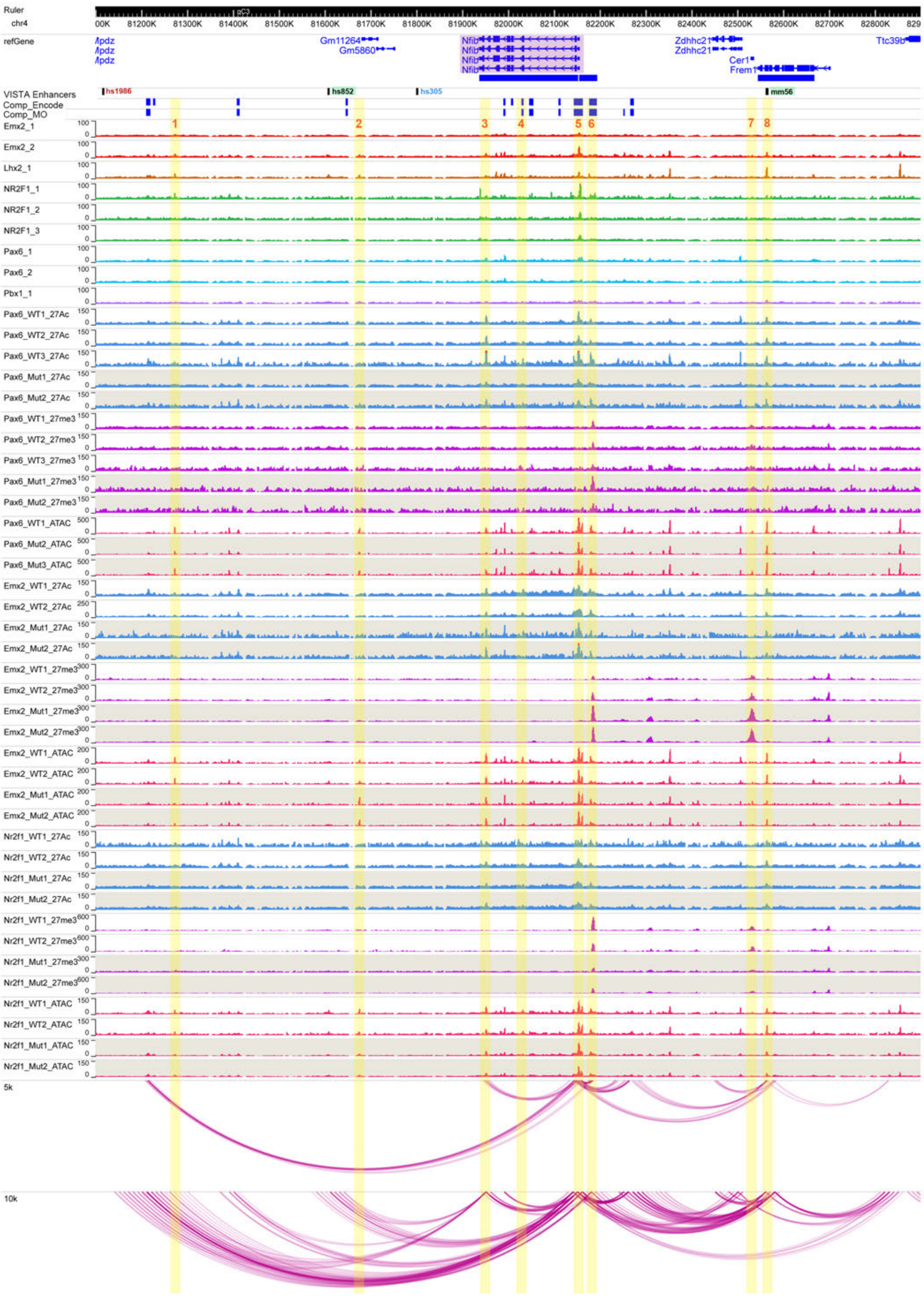

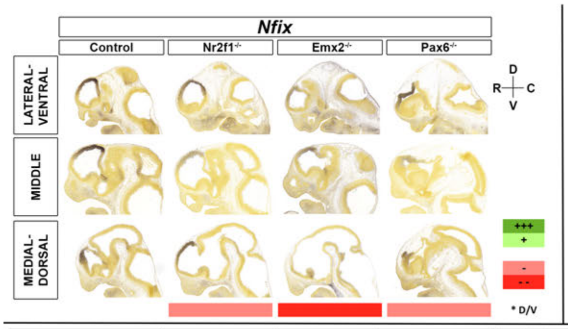

		Motifs					TF ChIP Seq					Differential epigenomic marks in WT vs. Mutant									Plac-Seq	Computation	Genomic features
												Pax6 mutant			Emx2 mutant			Nr2f1 mutant					
		Emx2 Hb	Nr2f1	Pax6 Hb	Pax6 P	Pbx1	Emx2	Lhx2	Nr2f1	Pax6	Pbx1	27Ac	27me3	ATAC	27Ac	27me3	ATAC	27Ac	27me3	ATAC			
Nfix	chr8:87060000-87430000																						
1	chr8:87183906-87187000	x							x			Loss		Same	Loss		Same	Loss		Same	x		
2	chr8:87210441-87212579											Loss		Same	Same		Same	Loss		Same	x		Nacc1 TSS
3	chr8:87225214-87232338								x				Same			Gain			Same		x	x	Nfix 5'UTR
4	chr8:87295475-87304533	x				x		x	x	x			Same		Loss	Gain			Loss			x	Nfix TSS
5	chr8:87306480-87314255								x				Same			Gain			Same			x	
6	chr8:87319898-87327594													Same		Gain	Same			Same		x	Nfix alternative TSS

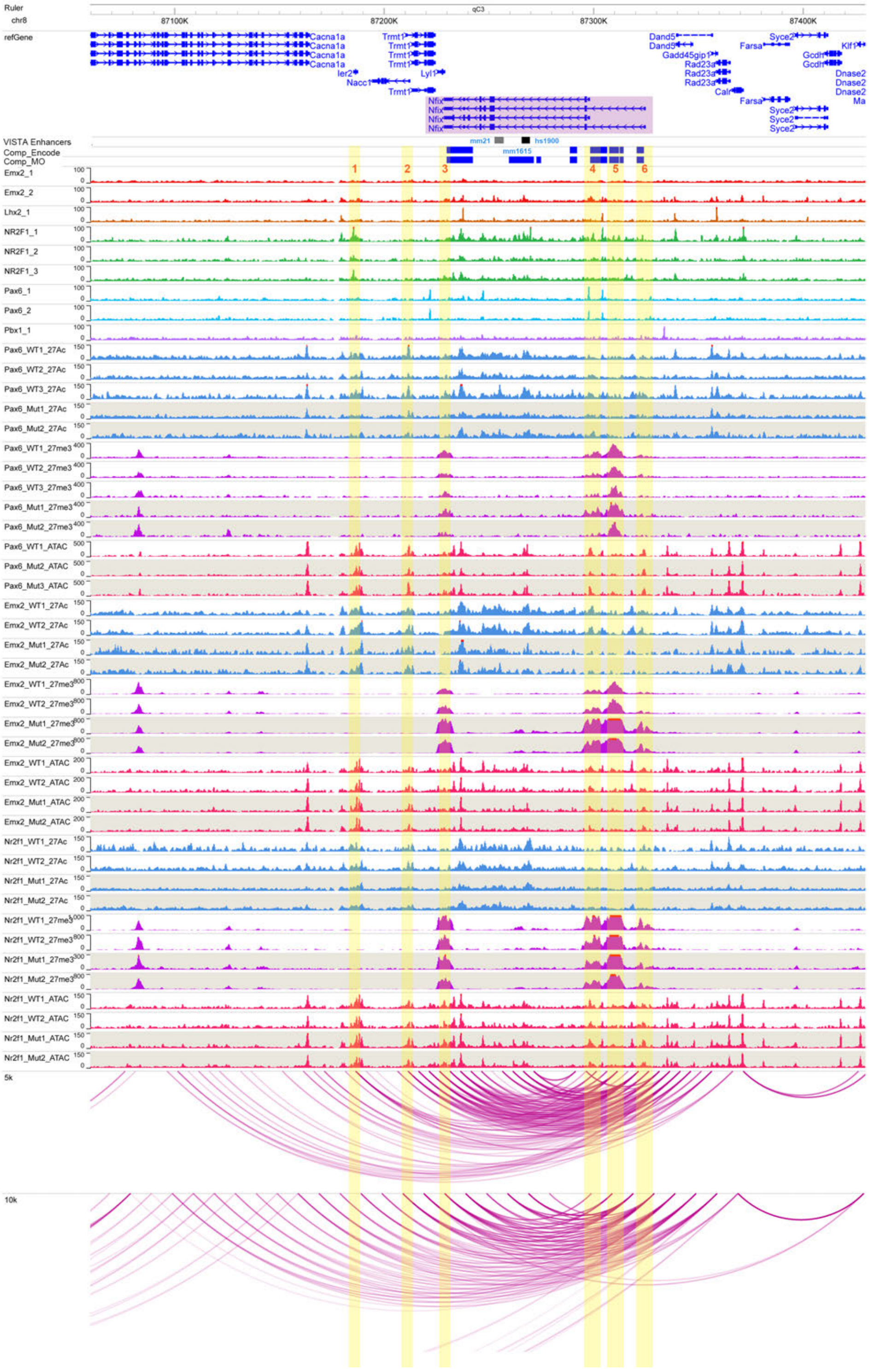

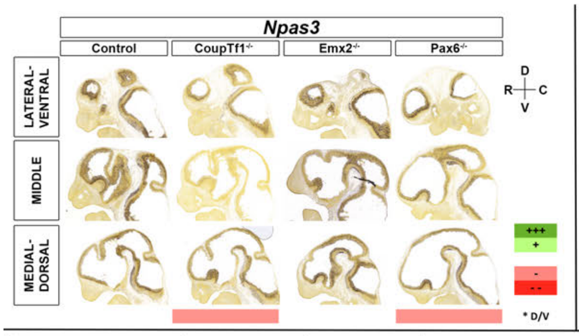

		Motifs					TF ChIP Seq					Differential epigenomic marks in WT vs. Mutant									Plac-Seq	Computation	Genomic features
												Pax6 mutant			Emx2 mutant			Nr2f1 mutant					
		Emx2 HB	Nr2f1	Pax6 Hb	Pax6 P	Pbx1	Emx2	Lhx2	Nr2f1	Pax6	Pbx1	27Ac	27me3	ATAC	27Ac	27me3	ATAC	27Ac	27me3	ATAC			
Npas3	chr12:53900000-55700000																						
1	chr12:54162201-54164479	x	x				x	x			x	Loss		Same	Loss		Same	Loss		Same	x	x	mJR97
2	chr12:54212422-54214798	x					x	x			x			Same			Loss			Loss	x		
3	chr12:54224810-54226545	x					x	x		x	x			Same			Loss			Loss			
4	chr12:54347665-54350320											Loss		Same	Same		Same	Loss		Same		x	Npas3 TSS
5	chr12:54353494-54355559	x					x			x	x	Loss		Same	Same		Same	Loss		Same		x	Npas3 Gene Body
6	chr12:54391045-54394848						x		x		x	Loss		Same	Same		Same	Loss				x	Npas3 Gene Body
7	chr12:54548080-54550100						x	x	x	x	x			Loss		Small gain	Same			Same		x	mJR94
8	chr12:54593760-54594932	x					x	x	x		x			Same			Loss			Same	x		Npas3 Gene Body
9	chr12:54723872-54727081	x		x	x	x	x	x	x	x		Loss		Loss	Same		Same	Same?		Same	x	x	hs527

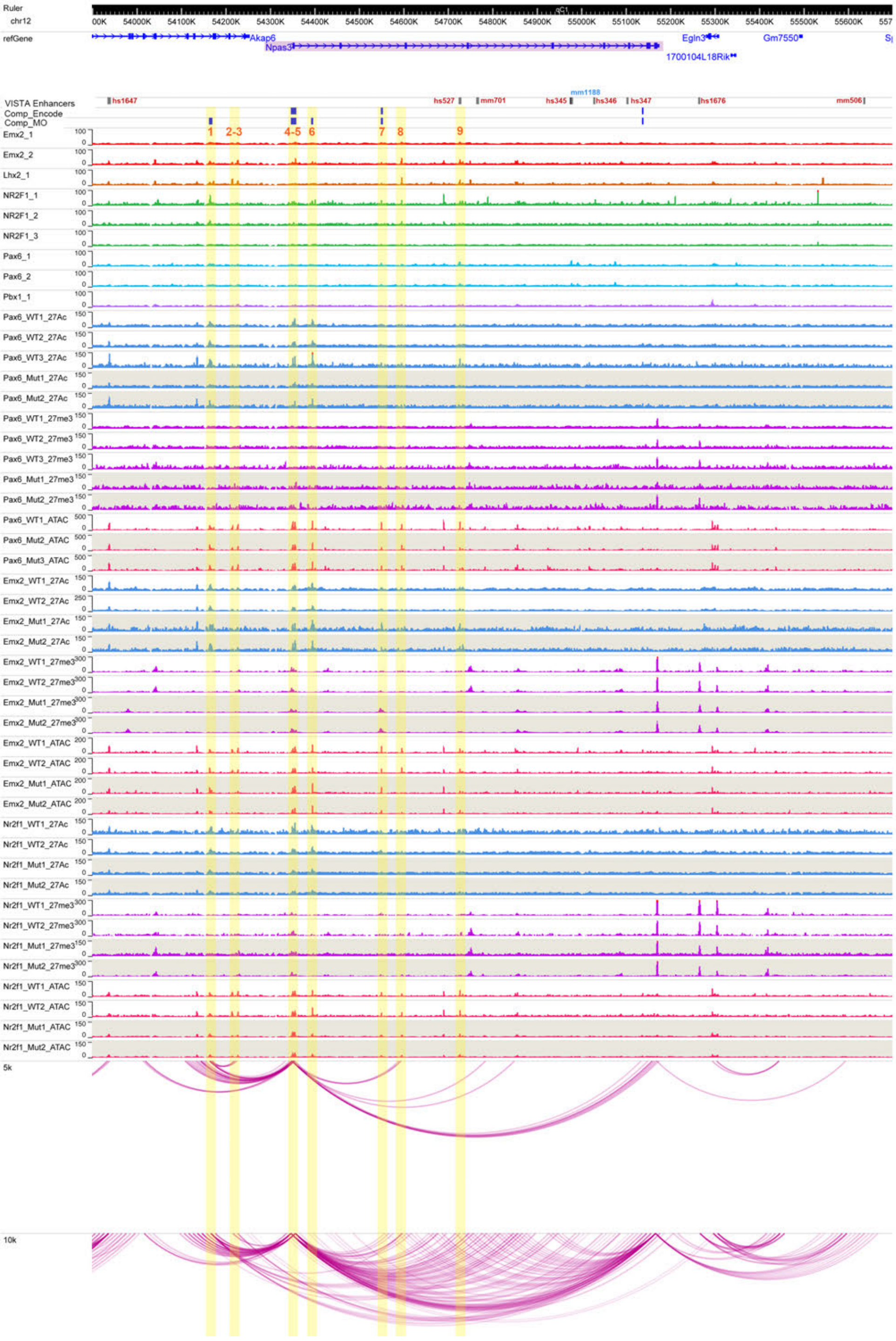

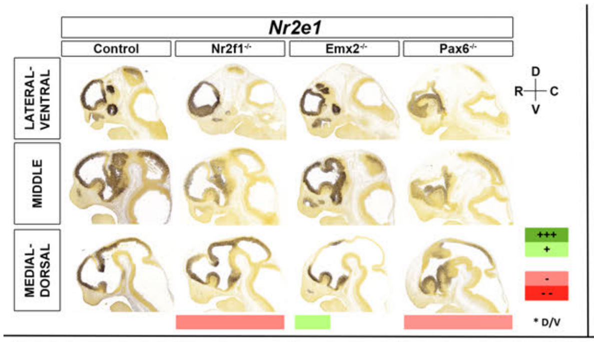

		Motifs					TF ChIP Seq					Differential epigenomic marks in WT vs. Mutant									Plac-Seq	Computation	Genomic features
												Pax6 mutant			Emx2 mutant			Nr2f1 mutant					
		Emx2 HB	Nr2f1	Pax6 Hb	Pax6 P	Pbx1	Emx2	Lhx2	Nr2f1	Pax6	Pbx1	27Ac	27me3	ATAC	27Ac	27me3	ATAC	27Ac	27me3	ATAC			
Nr2E1	chr10:41950000-42460000																						
1	chr10:42297819-42300842						x		x	x		Loss		Loss	Same		Same	Same		Same	x	x	Nr2E1 Intron
2	chr10:42302763-42304738	x	x	x	x		x	x	x	x	x	Loss		Same	Same		Same	Loss		Same	x	x	Nr2E1 TSS
3	chr10:42310968-42312329	x					x		x	x		Loss		Same	Same		Same	Loss		Same		x	
4	chr10:42350739-42352822	x		x	x		x	x	x	x		Same		Same	Same		Same	Loss		Same		x	

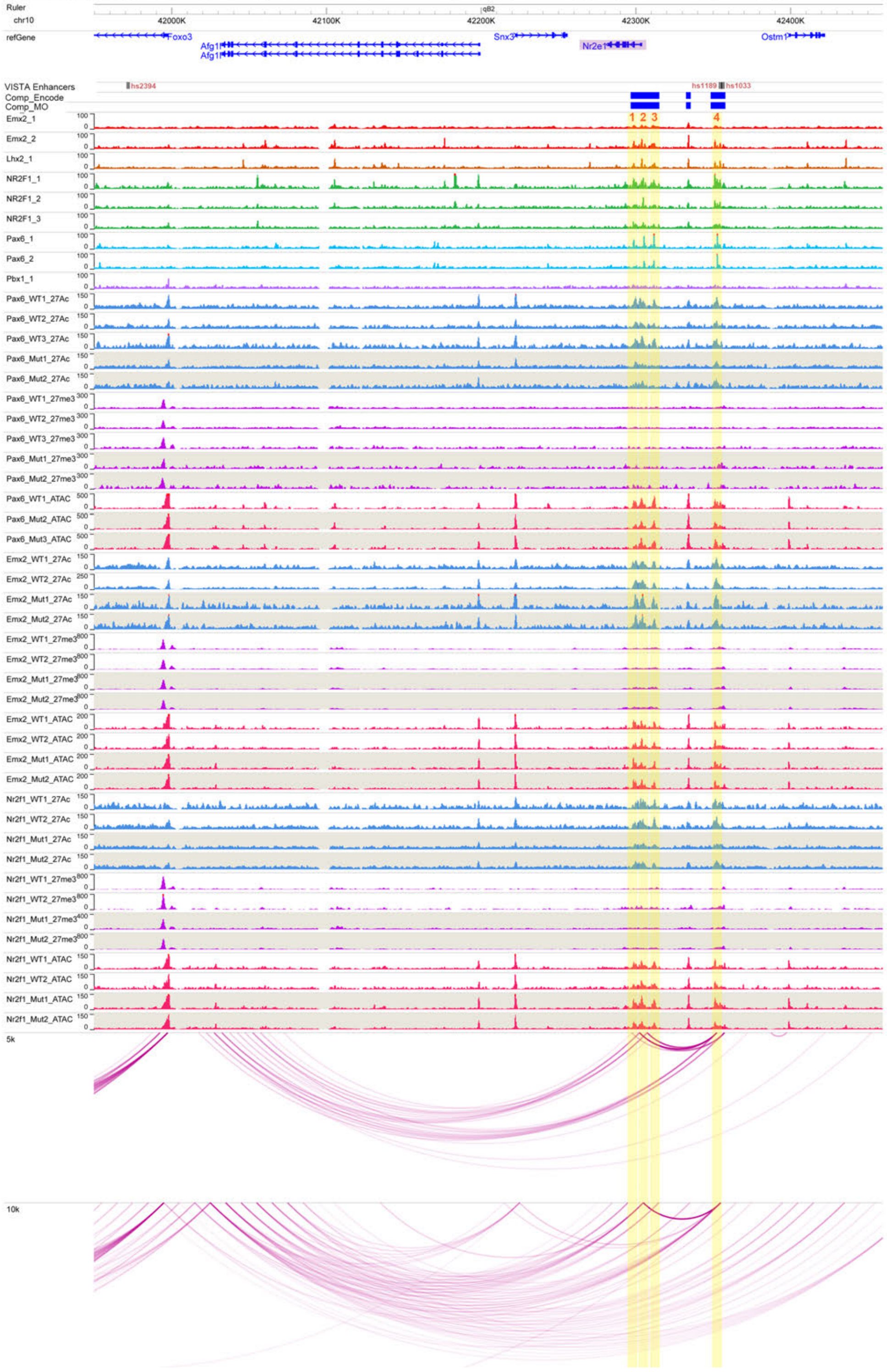

Nr2f1

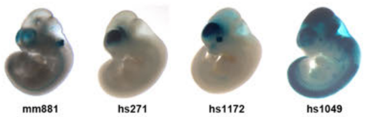

		Motifs					TF ChIP Seq					Differential epigenomic marks in WT vs. Mutant									Plac-Seq	Computation	Genomic features
												Pax6 mutant			Emx2 mutant			Nr2f1 mutant					
		Emx2 HB	Nr2f1	Pax6 Hb	Pax6 P	Pbx1	Emx2	Lhx2	Nr2f1	Pax6	Pbx1	27Ac	27me3	ATAC	27Ac	27me3	ATAC	27Ac	27me3	ATAC			
**Nr2f1**	**chr13:77500000-79323000**																						
**1**	chr13:77535859-77537676	x					x	x	x			Same		Same	Loss		Loss			Same	x		hs273
**2**	chr13:78031585-78032706	x					x	x	x	x	x	Loss		Same	Loss	Gain	Same	Same		Same	x		hs271
**3**	chr13:78033028-78033775	x					x	x	x	x		Loss		Same	Loss	Gain	Loss	Loss		Same	x		
**4**	chr13:78238631-78240287	x					x	x		x		Loss		Loss	Loss		Loss	Same		Same		x	
**5**	chr13:78301285-78303743	x					x		x			Loss		Same	Same	Same	Same	Same	Same	Same		x	
**6**	chr13:78318156-78320079	x					x		x	x		Loss	Gain	Same	Loss	Gain	Same	Loss	Loss	Same		x	
**7**	chr13:78320294-78321148	x					x					Same	Gain	Same	Loss	Gain	Same	Same	Loss	Same		x	
**8**	chr13:78333521-78334986											Loss	Same	Same	Loss	Gain	Same	Loss	Loss	Same		x	Nr2f1 Gene Body
**9**	chr13:78336209-78338805	x					x			x		Loss	Same	Same	Loss	Gain	Same	Loss	Loss	Loss		x	Nr2f1 TSS
**10**	chr13:78339478-78340485	x					x	x	x					Same		Gain	Loss		Loss	Same			
**11**	chr13:78341891-78344303								x			Same		Same	Loss	Gain	Loss	Same	Loss	Same		x	
**12**	chr13:78348092-78349649	x					x	x				Loss	Same	Loss	Loss	Gain	Same		Loss	Same		x	
**13**	chr13:78565590-78568155	x					x	x		x		Loss		Same	Loss	Gain	Same	Same		Same	x	x	
**14**	chr13:78583826-78587247						x		x	x		Loss		Loss	Loss	Gain	Same	Same		Same	x		hs1550
**15**	chr13:78719884-78721593	x		x	x	x	x	x	x	x	x			Same	Loss	Gain	Same			Same	x		hs1172
**16**	chr13:78781101-78783086	x					x	x			x	Loss		Loss	Loss		Loss	Same		Same	x		

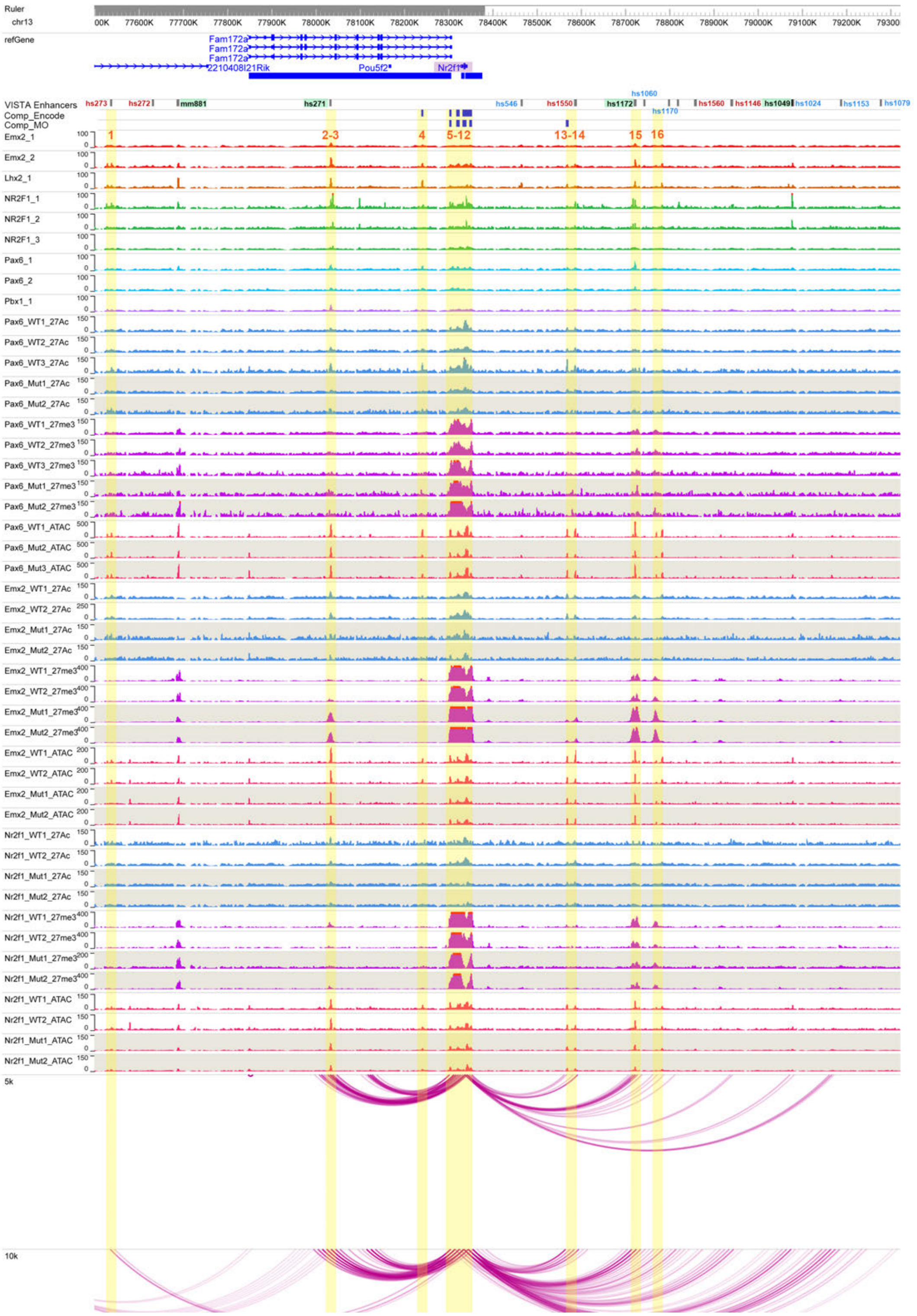

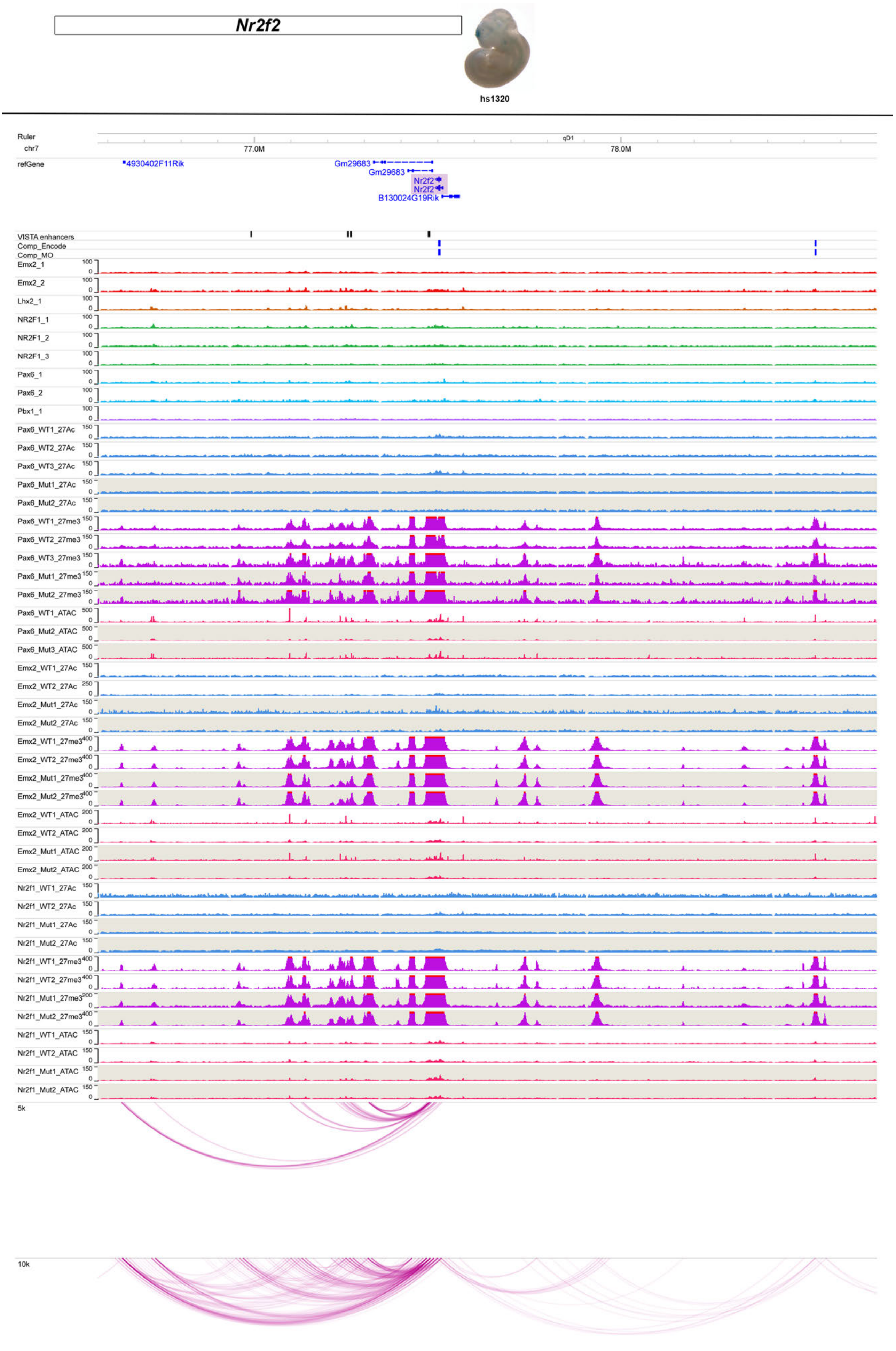
Nr2f2
hs1320
Ruler
chr7
qD1
77.0M
78.0M
refGene
4930402F11Rik
Gm29683
Gm29683
Nr2f2
Nr2f2
B130024G19Rik
VISTA enhancers
Comp_Encode
Comp_MO
Emx2_1
Emx2_2
Lhx2_1
NR2F1_1
NR2F1_2
NR2F1_3
Pax6_1
Pax6_2
Pbx1_1
Pax6_WT1_27Ac
Pax6_WT2_27Ac
Pax6_WT3_27Ac
Pax6_Mut1_27Ac
Pax6_Mut2_27Ac
Pax6_WT1_27me3
Pax6_WT2_27me3
Pax6_WT3_27me3
Pax6_Mut1_27me3
Pax6_Mut2_27me3
Pax6_WT1_ATAC
Pax6_Mut2_ATAC
Pax6_Mut3_ATAC
Emx2_WT1_27Ac
Emx2_WT2_27Ac
Emx2_Mut1_27Ac
Emx2_Mut2_27Ac
Emx2_WT1_27me3
Emx2_WT2_27me3
Emx2_Mut1_27me3
Emx2_Mut2_27me3
Emx2_WT1_ATAC
Emx2_WT2_ATAC
Emx2_Mut1_ATAC
Emx2_Mut2_ATAC
Nr2f1_WT1_27Ac
Nr2f1_WT2_27Ac
Nr2f1_Mut1_27Ac
Nr2f1_Mut2_27Ac
Nr2f1_WT1_27me3
Nr2f1_WT2_27me3
Nr2f1_Mut1_27me3
Nr2f1_Mut2_27me3
Nr2f1_WT1_ATAC
Nr2f1_WT2_ATAC
Nr2f1_Mut1_ATAC
Nr2f1_Mut2_ATAC
5k
10k

Pax6

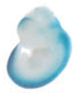

hs1531

		Motifs					TF ChIP Seq					Differential epigenomic marks in WT vs. Mutant									Plac-Seq	Computation	Genomic features
												Pax6 mutant			Emx2 mutant			Nr2f1 mutant					
		Emx2 Hb	Nr2f1	Pax6 Hb	Pax6 P	Pbx1	Emx2	Lhx2	Nr2f1	Pax6	Pbx1	27Ac	27me3	ATAC	27Ac	27me3	ATAC	27Ac	27me3	ATAC			
Pax6	chr2:105085000-106050000																						
1	chr2:105355979-105356970						x	x	x	x	x	Loss		Same	Same		Same	Same		Same			hs855
2	chr2:105451245-105454219						x	x				Same		Same	Same		Same	Loss		Same			hs1531
3	chr2:105502815-105507481												Loss			Same			Same				Pax6 TSS
4	chr2:105558877-105560976						x	x	x	x	x	Loss		Same	Loss		Same	Same		Same			hs198

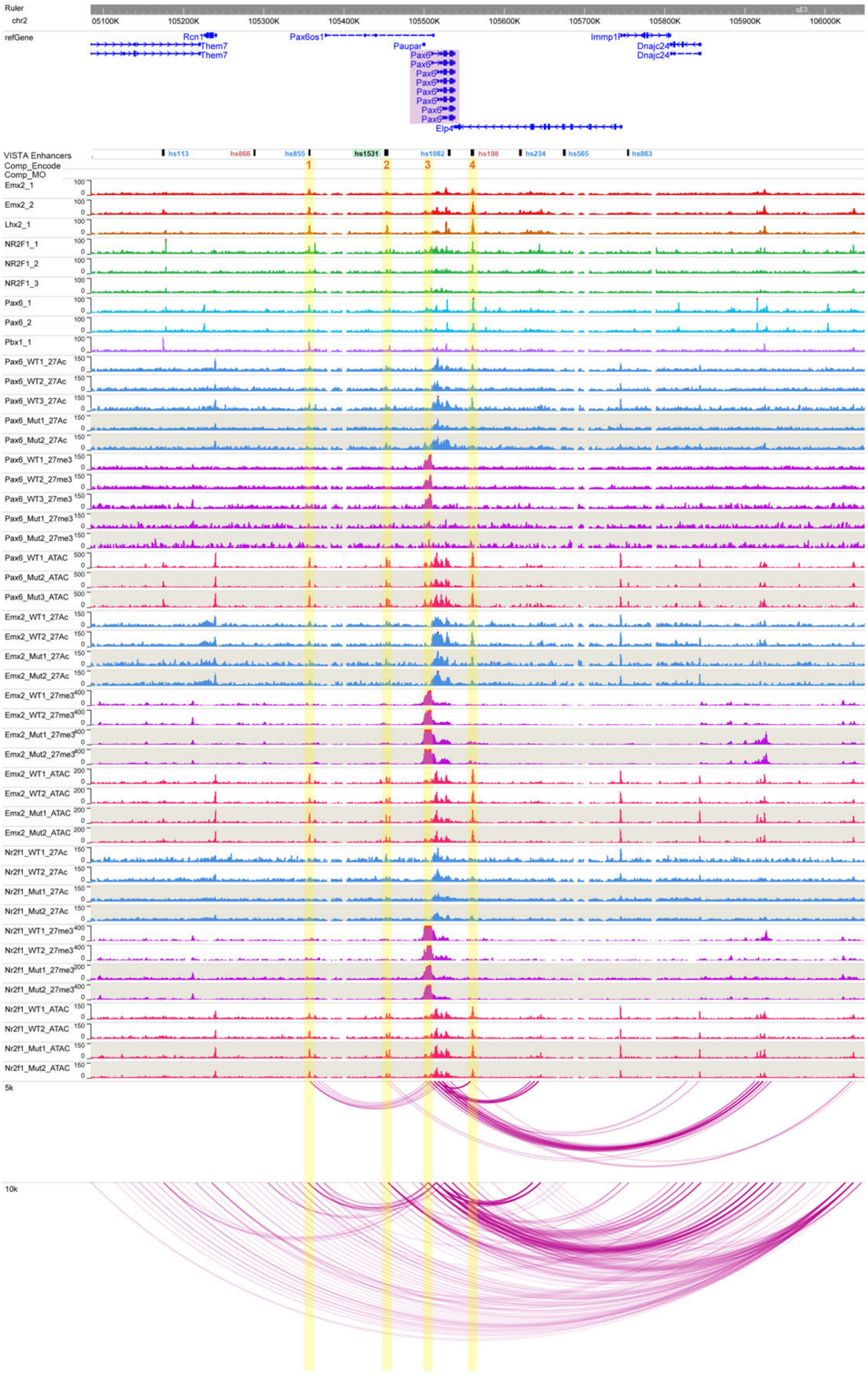

Pbx1

		Motifs					TF ChIP Seq					Differential epigenomic marks in WT vs. Mutant									Plac-Seq	Computation	Genomic features
												Pax6 mutant			Emx2 mutant			Nr2f1 mutant					
		Emx2 HB	Nr2f1	Pax6 Hb	Pax6 P	Pbx1	Emx2	Lhx2	Nr2f1	Pax6	Pbx1	27Ac	27me3	ATAC	27Ac	27me3	ATAC	27Ac	27me3	ATAC			
Pbx1	chr1:169860000-171370500																						
1	chr1:169975190-169978135						x	x	x	x	x	Same		Loss	Loss		Loss	Same		Same	x		
2	chr1:170175012-170176937						x		x		x	Loss		Same	Loss		Same	Loss		Same	x		Pbx1 Gene Body
3	chr1:170229514-170231269											Loss			Loss			Loss			x		hs1144
4	chr1:170257128-170258791						x	x				Same		Same	Same		Loss	Same		Same	x		hs203
5	chr1:170321692-170324410						x	x	x	x		Same		Loss	Loss		Loss	Loss			x	x	Pbx1 Gene Body
6	chr1:170340225-170341639						x							Loss			Same			Same		x	Pbx1 Gene Body
7	chr1:170360324-170363111								x		x	Loss		Same	Same		Same	Same		Same		x	Pbx1 TSS
8	chr1:170837816-170839853						x	x	x		x	Loss		Same	Loss		Loss	Same		Same	x		hs202

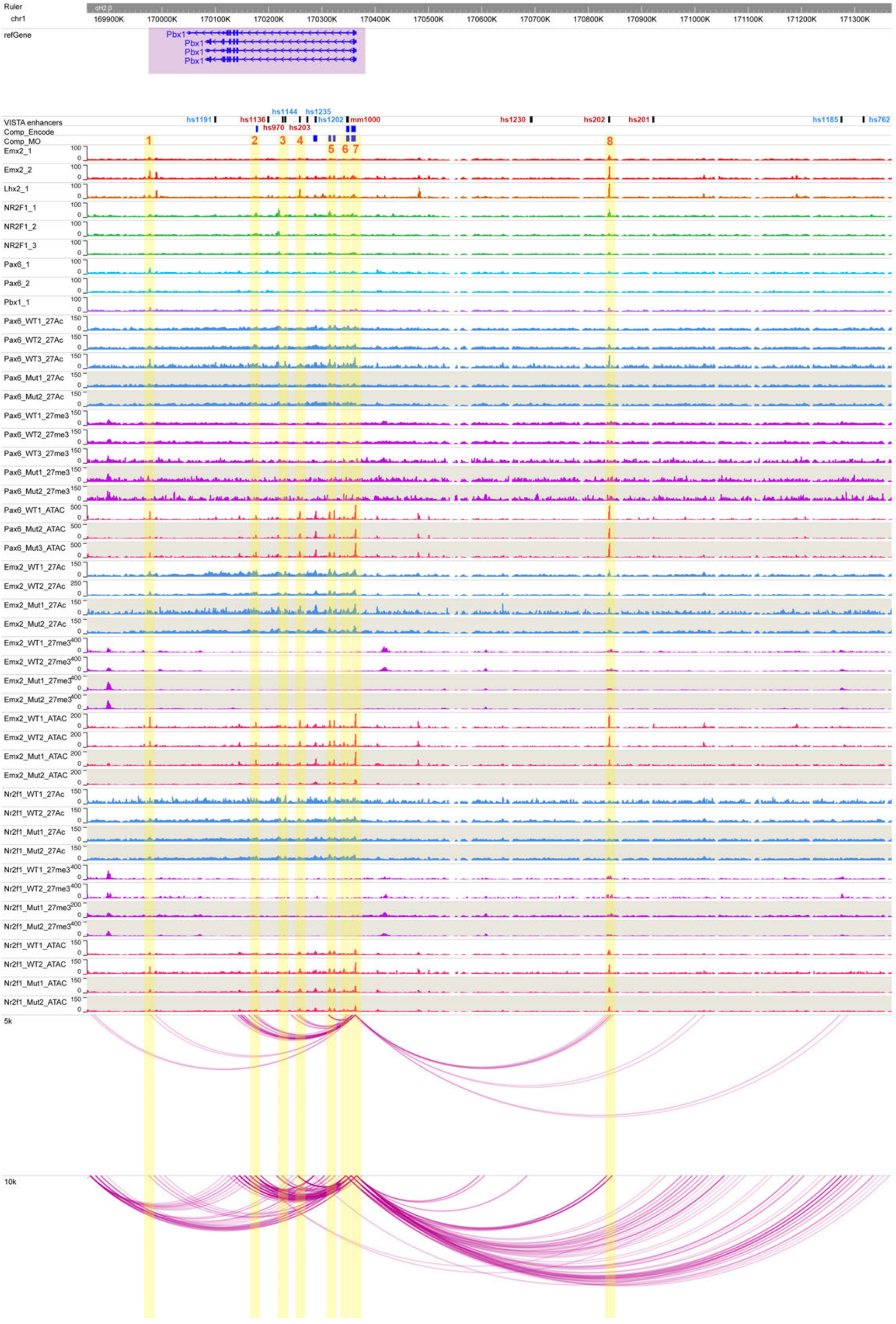

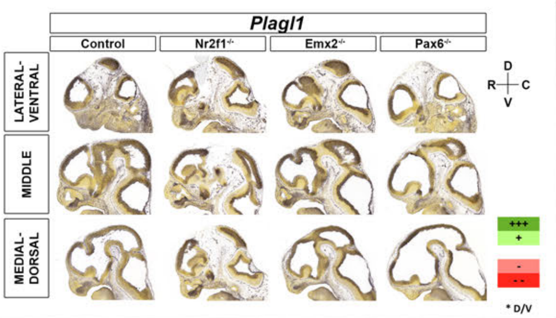

		Motifs					TF ChIP Seq					Differential epigenomic marks in WT vs. Mutant									Plac-Seq	Computation	Genomic features
												Pax6 mutant			Emx2 mutant			Nr2f1 mutant					
		Emx2 HB	Nr2f1	Pax6 Hb	Pax6 P	Pbx1	Emx2	Lhx2	Nr2f1	Pax6	Pbx1	27Ac	27me3	ATAC	27Ac	27me3	ATAC	27Ac	27me3	ATAC			
Plagl1	chr10:12700000-13310000																						
1	chr10:12809880-12812996											Loss		Same	Loss		Same	Same		Same			Plagl1 TSS
2	chr10:12826983-12831335	x					x	x	x	x	x	Loss		Same	Loss		Same	Same		Same		x	Plagl1 Gene body
3	chr10:12836410-12839285	x	x	x	x		x	x	x	x		Loss		Same	Loss		Same	Same		Same		x	Plagl1 Gene body
4	chr10:13043102-13045156	x	x						x			Loss		Same	Same		Same	Loss		Same	x		Phactr2 TSS
5	chr10:13135354-13136688	x					x	x	x			Loss		Same	Loss		Same	Same		Same	x		

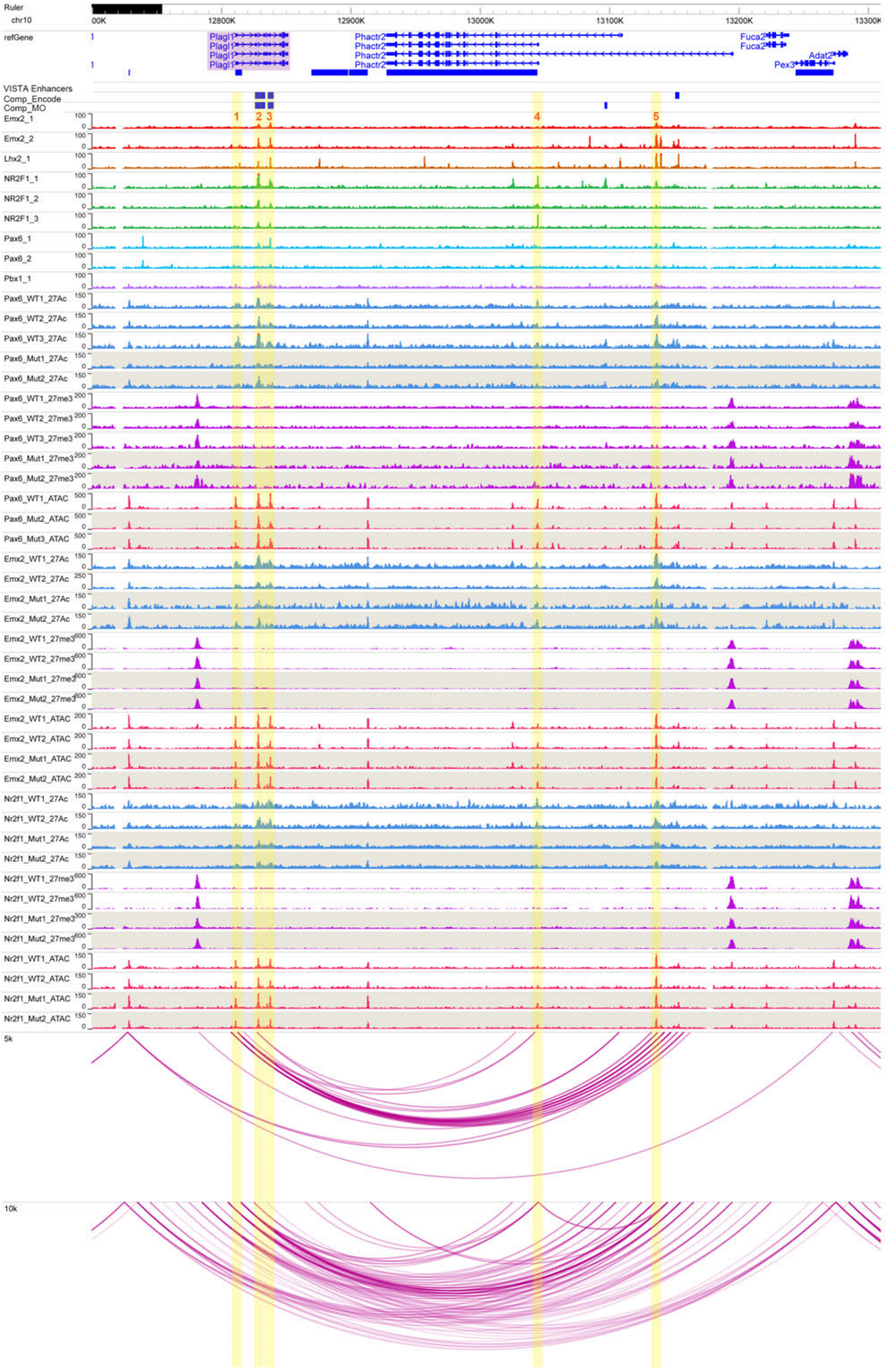

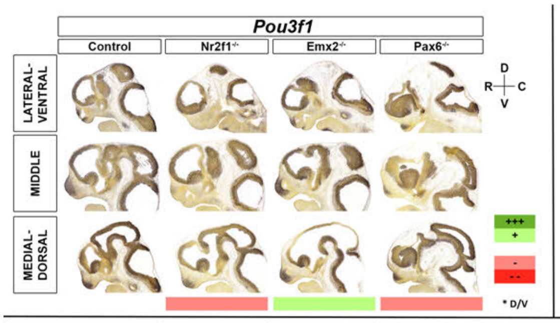

		Motifs					TF ChIP Seq					Differential epigenomic marks in WT vs. Mutant									Plac-Seq	Computation	Genomic features
												Pax6 mutant			Emx2 mutant			Nr2f1 mutant					
		Emx2 HB	Nr2f1	Pax6 Hb	Pax6 P	Pbx1	Emx2	Lhx2	Nr2f1	Pax6	Pbx1	27Ac	27me3	ATAC	27Ac	27me3	ATAC	27Ac	27me3	ATAC			
Pou3f1	chr4:123550000-124550000																						
1	chr4:123711996-123714726	x		x	x		x		x			Loss		Same	Loss		Same	Same		Same		x	hs1031
2	chr4:124072428-124078145	x		x	x	x	x	x	x		x		Same	Same		Loss	Loss		Same	Same	x		hs270
3	chr4:124118120-124122688			x	x					x			Same			Loss			Same		x		
4	chr4:124132233-124133640												Same			Loss			Same		x		hs1008
5	chr4:124135319-124138674												Same			Loss			Same		x		hs2088
6	chr4:124142356-124147119	x					x	x	x	x	x		Same	Same		Loss	Loss		Same		x		
7	chr4:124154196-124157660								x				Same			Loss			Same		x		hs1001
8	chr4:124335186-124337892												Same			Loss			Loss				Pou3f1 gene body

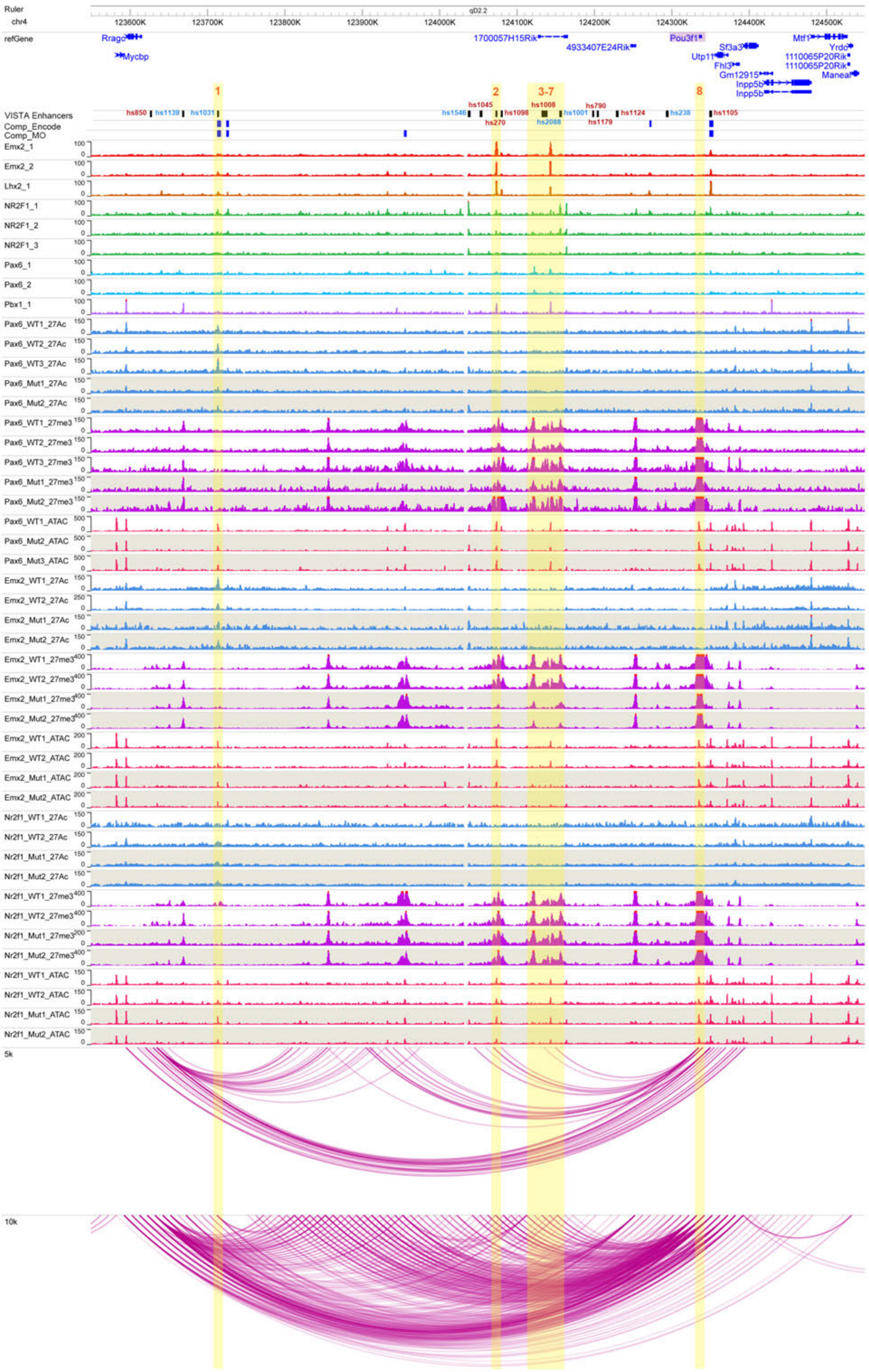

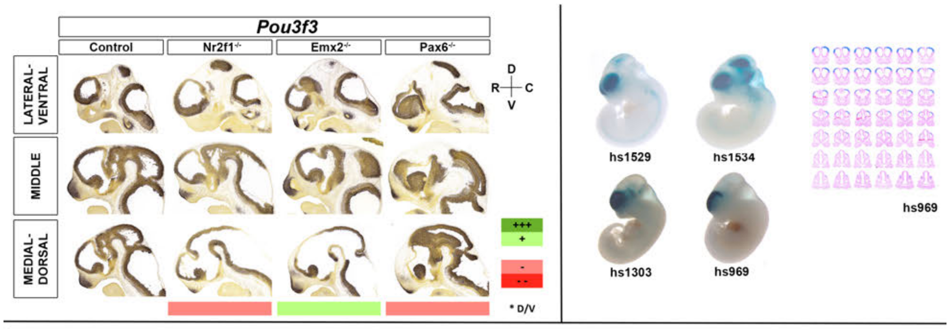

		Motifs					TF ChIP Seq					Differential epigenomic marks in WT vs. Mutant									Plac-Seq	Computation	Genomic features
												Pax6 mutant			Emx2 mutant			Nr2f1 mutant					
		Emx2 HB	Nr2f1	Pax6 Hb	Pax6 P	Pbx1	Emx2	Lhx2	Nr2f1	Pax6	Pbx1	27Ac	27me3	ATAC	27Ac	27me3	ATAC	27Ac	27me3	ATAC			
Pou3f3	chr1:41700000-42857055																						
1	chr1:41798138-41801834	x		x	x		x	x	x	x	x	Same		Loss	Same		Same	Same		Same	x		
2	chr1:41870353-41872353	x					x	x	x	x	x	Loss		Same	Same		Same	Same?		Same	x	x	hs1529
3	chr1:41992227-41995700	x		x	x		x	x		x	x		Loss	Loss		Loss	Loss		Same	Same	x		
4	chr1:42313865-42317133	x		x		x	x		x	x	x	Loss		Same	Same		Same	Loss		Same	x	x	hs960
5	chr1:42595469-42598795	x					x	x	x	x	x	Loss		Same	Same		Same	Loss		Same	x	x	hs969
6	chr1:42746840-42749611						x		x	x		Loss		Same	Gain		Same	Loss		Same			
7	chr1:42753952-42757047								x			Loss			Gain			Loss				x	Pou3f3 gene body
8	chr1:42769552-42771799		x						x		x	Loss		Same	Gain		Same	Loss		Same			

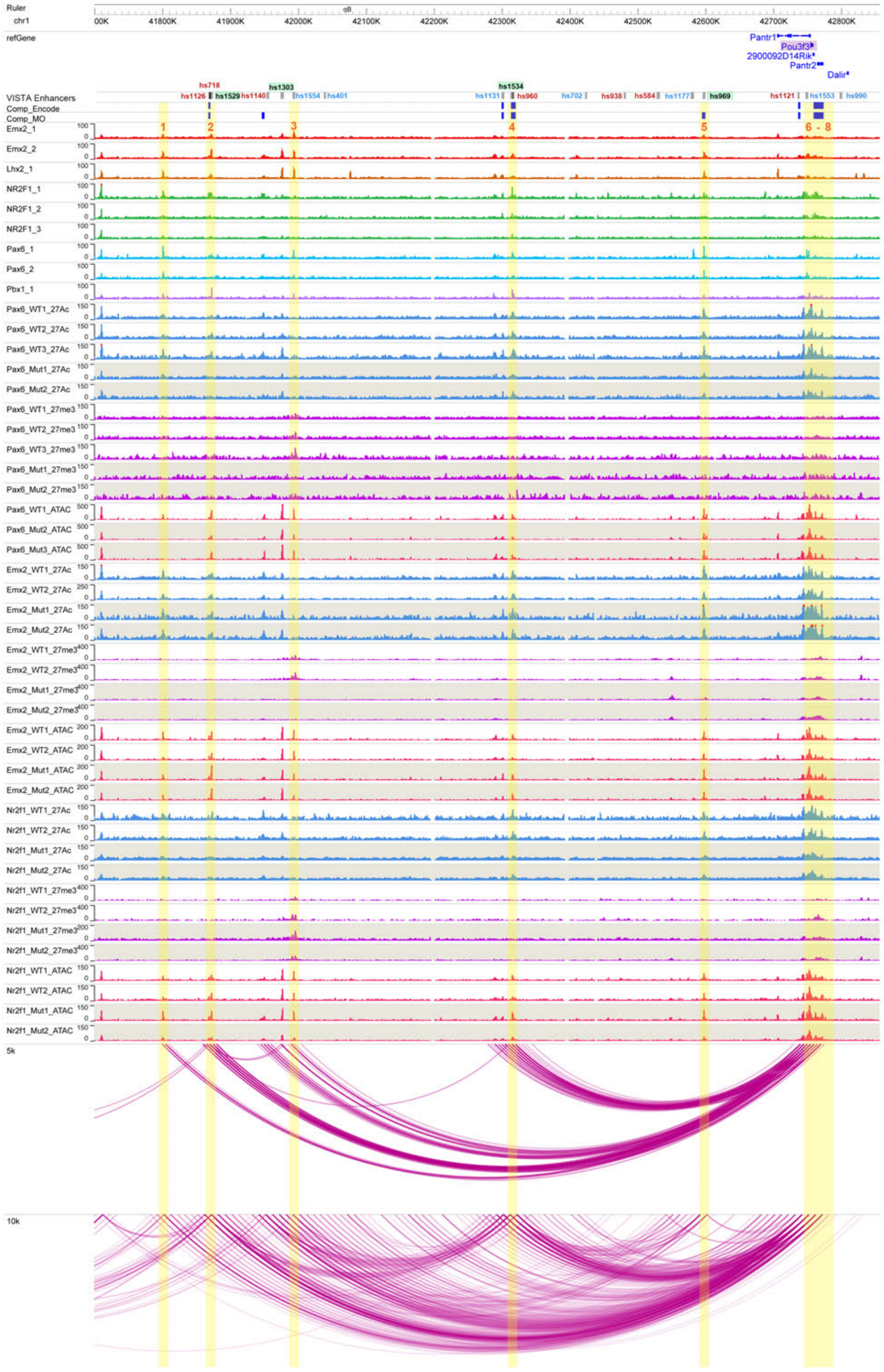

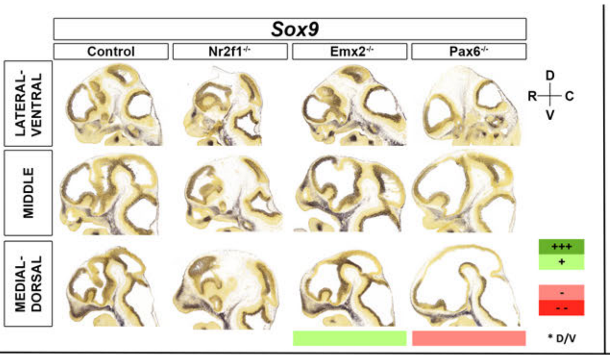

		Motifs					TF ChIP Seq					Differential epigenomic marks in WT vs. Mutant									Plac-Seq	Computation	Genomic features
												Pax6 mutant			Emx2 mutant			Nr2f1 mutant					
		Emx2 HB	Nr2f1	Pax6 Hb	Pax6 P	Pbx1	Emx2	Lhx2	Nr2f1	Pax6	Pbx1	27Ac	27me3	ATAC	27Ac	27me3	ATAC	27Ac	27me3	ATAC			
Sox9	chr11:111700000-113500000																						
1	chr11:112058352-112060480	x		x	x	x	x	x	x	x	x	Loss		Same	Same		Same	Loss		Same			
2	chr11:112273236-112274832	x					x	x	x			Loss		Same	Loss		Loss	Loss		Same			mm634
3	chr11:112669970-112674132						x	x	x			Loss		Same	Loss		Same	Loss					
4	chr11:112838032-112839034	x					x	x	x			Loss		Same	Same		Same	Loss		Same	x	x	
5	chr11:112843512-112845082	x		x			x	x	x	x		Same		Loss	Same		Same	Same		Same	x		
6	chr11:112877902-112879155						x	x	x			Same		Same	Loss		Same	Same		Same	x	x	mm636
7	chr11:113300047-113301927			x			x		x			Same		Same	Loss		Same	Same		Same		x	

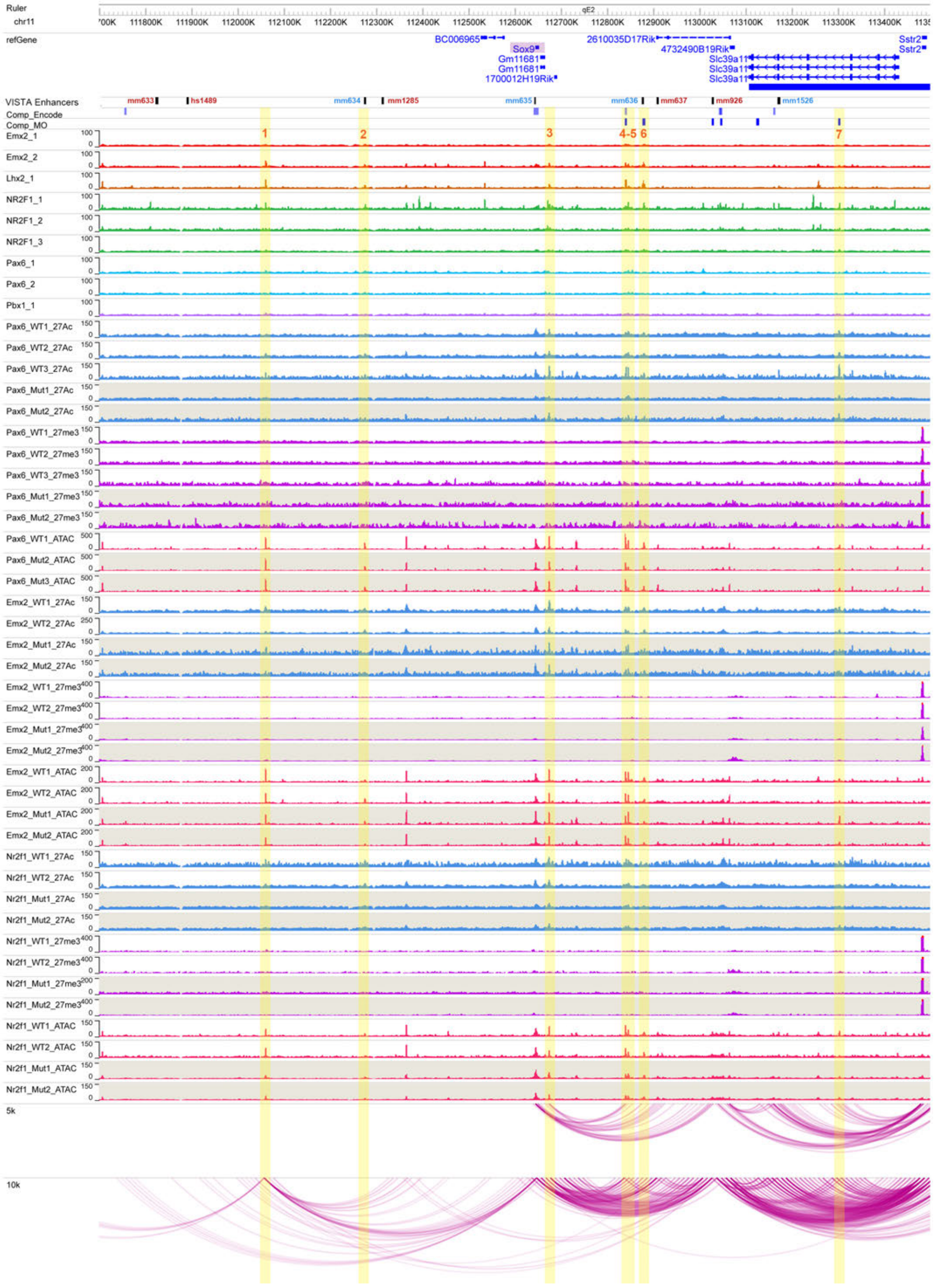

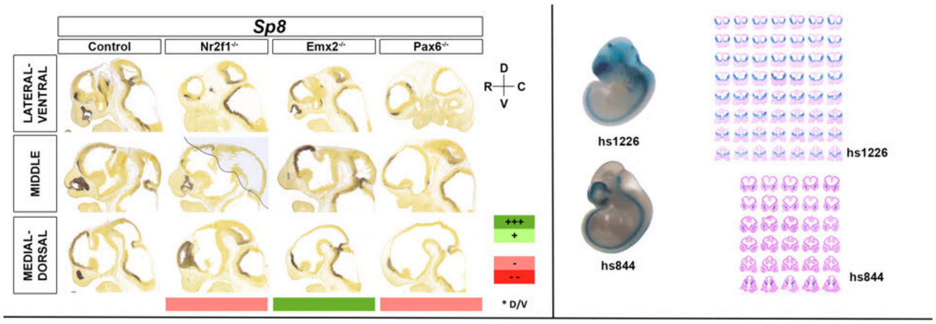

		Motifs					TF ChIP Seq					Differential epigenomic marks in WT vs. Mutant									Plac-Seq	Computation	Genomic features
												Pax6 mutant			Emx2 mutant			Nr2f1 mutant					
		Emx2 Hb	Nr2f1	Pax6 Hb	Pax6 P	Pbx1	Emx2	Lhx2	Nr2f1	Pax6	Pbx1	27Ac	27me3	ATAC	27Ac	27me3	ATAC	27Ac	27me3	ATAC			
Sp8	chr12:119000000-120191051																						
1	chr12:119043999-119045761	x		x	x		x	x		x	x		Same	Loss		Same	Same		Same	Same	x		hs807
2	chr12:119047456-119048848	x					x	x	x		x			Same			Loss			Same	x		
3	chr12:119266560-119267560	x						x					Same	Same		Loss			Same		x		
4	chr12:119639681-119646276												Loss			Loss			Same		x		
5	chr12:119767849-119769440	x		x	x		x		x			Loss		Loss	Loss		Same	Same		Same	x		
6	chr12:119888958-119890368			x			x		x	x	x			Loss			Gain			Same	x	x	hs1226
7	chr12:119897620-119901100	x		x			x	x	x	x	x		Same	Loss		Same	Same		Same	Same	x		
8	chr12:119957232-119961000	x					x	x	x				Same	Same		Loss	Same		Same	Same			
9	chr12:120019929-120024242	x					x	x	x				Same	Same		Loss			Same				
10	chr12:120078064-120080117	x					x	x	x	x		Loss	Gain	Loss	Same	Same	Same	Same	Same	Same		x	hs844
11	chr12:120084740-120088847											Loss	Gain	Same	Gain	Same	Same	Loss	Same	Same		x	Sp8 Gene body

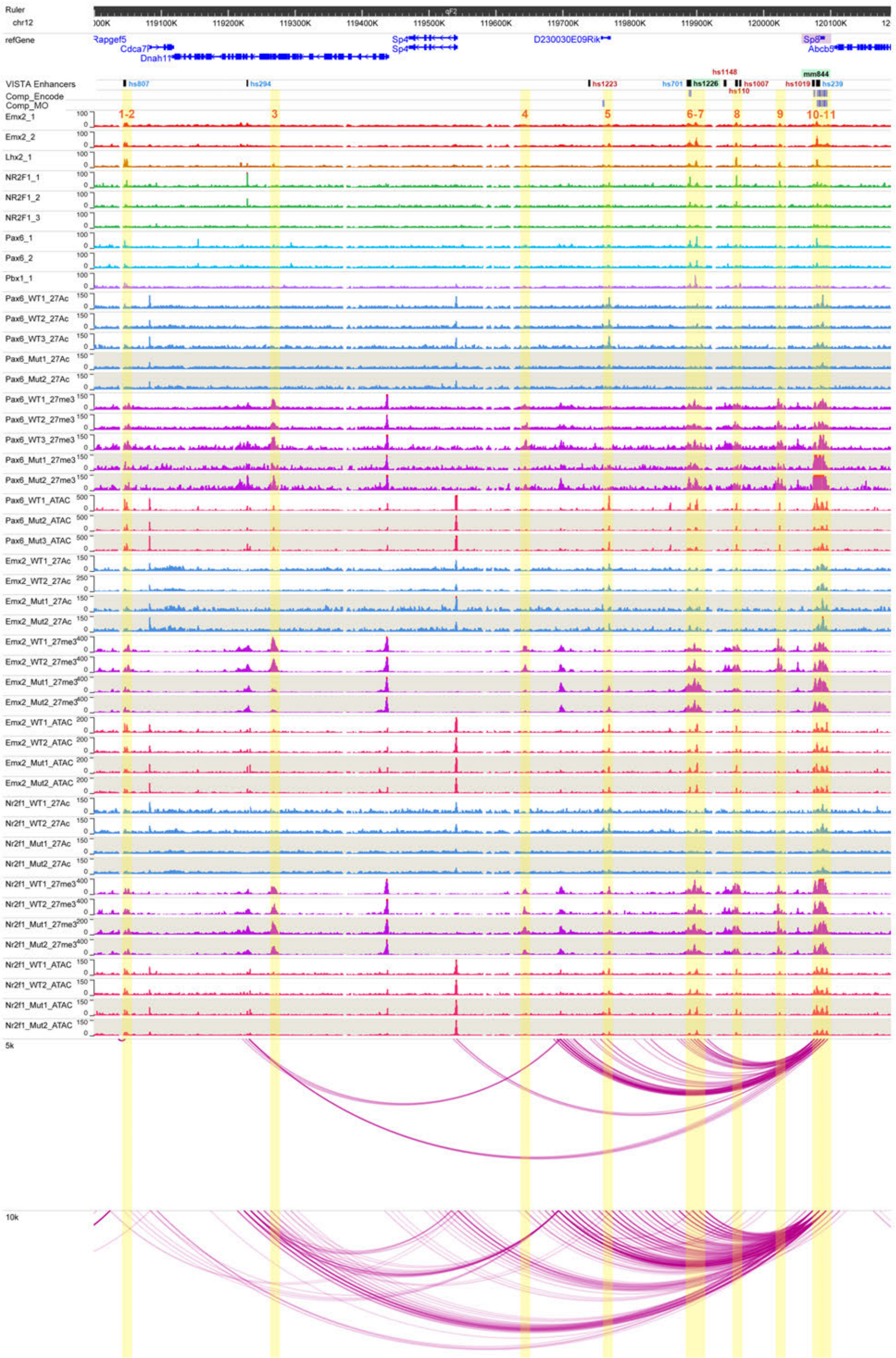

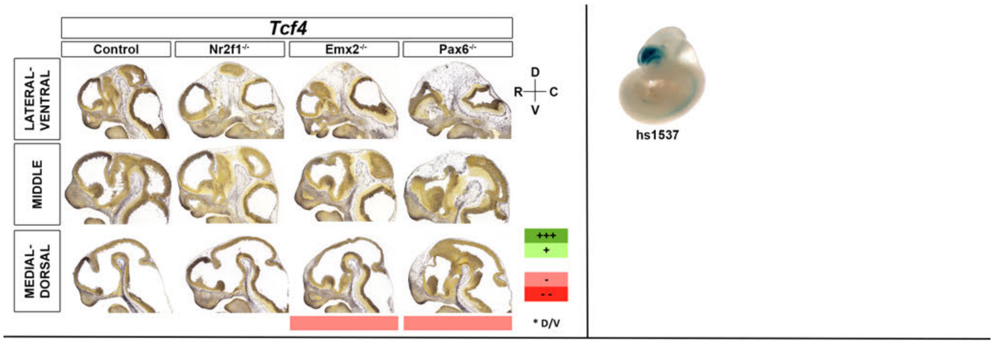

		Motifs					TF ChIP Seq					Differential epigenomic marks in WT vs. Mutant									Plac-Seq	Computation	Genomic features
												Pax6 mutant			Emx2 mutant			Nr2f1 mutant					
		Emx2 HB	Nr2f1	Pax6 Hb	Pax6 P	Pbx1	Emx2	Lhx2	Nr2f1	Pax6	Pbx1	27Ac	27me3	ATAC	27Ac	27me3	ATAC	27Ac	27me3	ATAC			
Tcf4	chr18:68600000-69947621																						
1	chr18:69357344-69358848								x				Same	Same		Gain	Loss		Same	Same	x	x	
2	chr18:69503409-69507465						x					Loss		Same	Loss		Same	Loss		Same		x	TCF4 TSS
3	chr18:69658711-69661831	x		x	x		x	x	x	x	x	Loss		Same	Loss		Loss	Loss		Same	x		hs376
4	chr18:69676248-69678881						x				x	Same		Same	Loss		Loss	Same		Same	x	x	
5	chr18:69721590-69724223	x				x	x	x	x	x	x	Loss		Same	Loss		Same	Same?		Same	x		hs1537
6	chr18:69751317-69754326	x		x	x		x	x	x	x	x	Loss		Same	Loss		Same	Same		Same	x	x	
7	chr18:69765992-69770130	x		x		x	x	x	x	x	x	Same		Same	Loss		Loss	Same		Same	x	x	hs1561

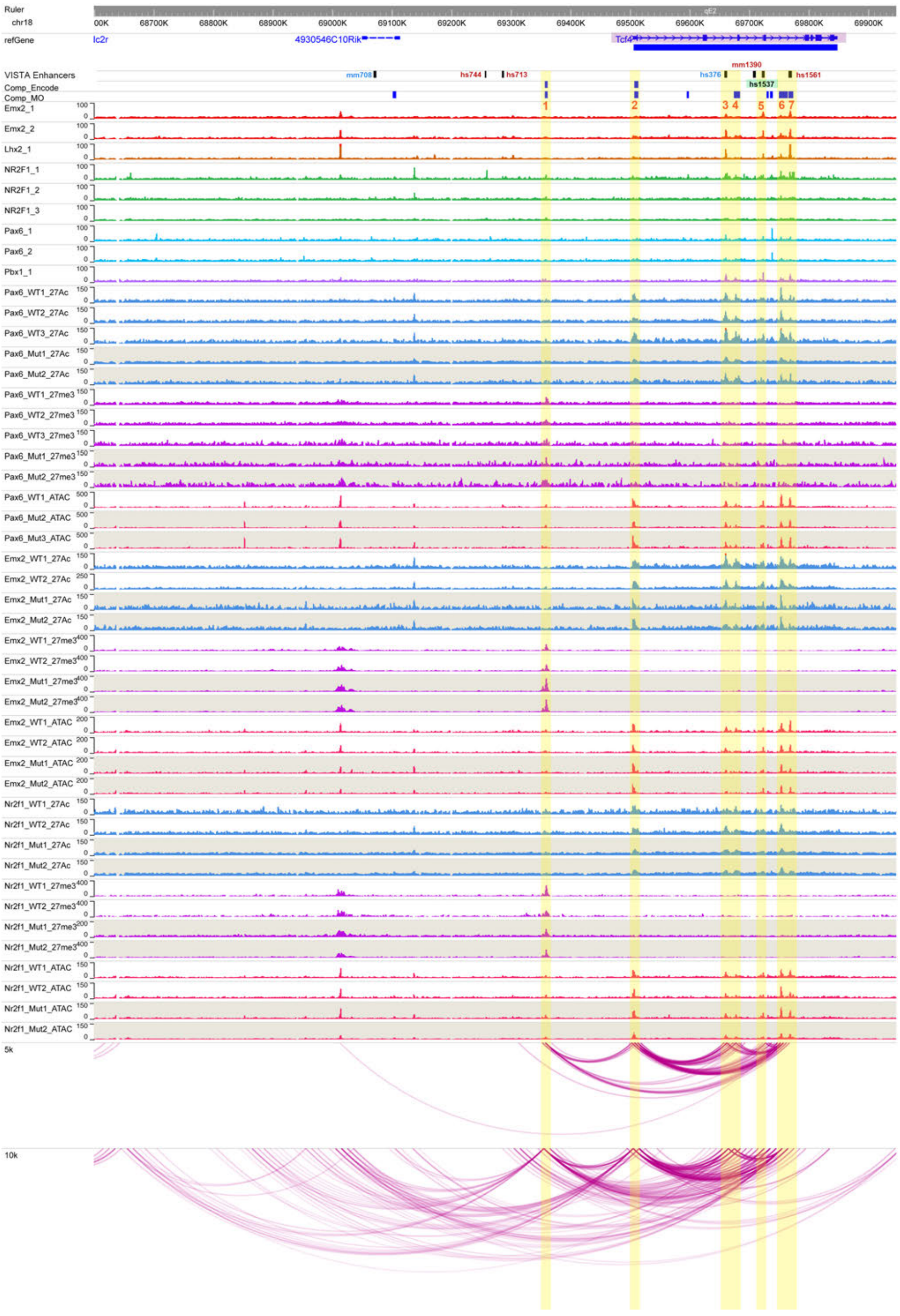

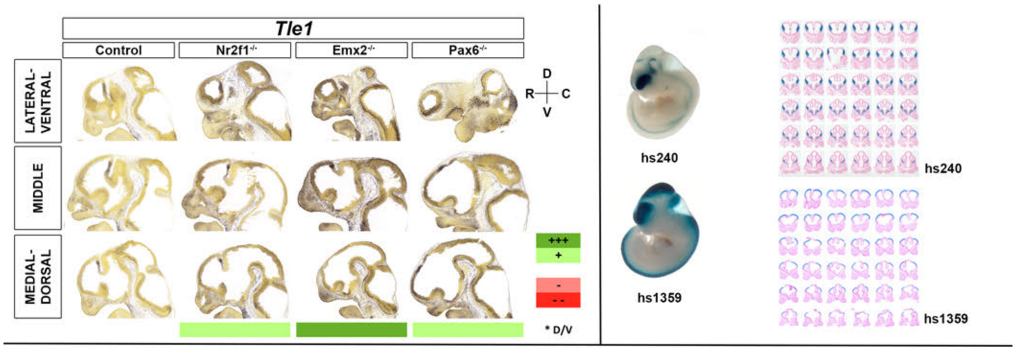

		Motifs					TF ChIP Seq					Differential epigenomic marks in WT vs. Mutant									Plac-Seq	Computation	Genomic features
												Pax6 mutant			Emx2 mutant			Nr2f1 mutant					
		Emx2 HB	Nr2f1	Pax6 Hb	Pax6 P	Pbx1	Emx2	Lhx2	Nr2f1	Pax6	Pbx1	27Ac	27me3	ATAC	27Ac	27me3	ATAC	27Ac	27me3	ATAC			
Tle1	chr4:71000000-72800000																						
1	chr4:71138280-71141616												Loss			Loss			Same		x		
2	chr4:71145257-71148745	x		x			x	x	x	x	x		Same	Same		Loss	Same		Same	Same	x		hs240
3	chr4:71594251-71595160	x		x	x		x	x						Same			Loss			Same	x		
4	chr4:71710362-71711705									x				Loss			Same			Same			
5	chr4:71806612-71808583						x		x	x				Loss	Same		Same	Same		Same			
6	chr4:71820673-71822175	x		x	x		x	x		x				Loss			Same			Same			
7	chr4:71859657-71862582					x	x		x		x	Loss	Loss	Same	Gain	Loss	Same	Loss	Same	Same		x	Tle1 TSS

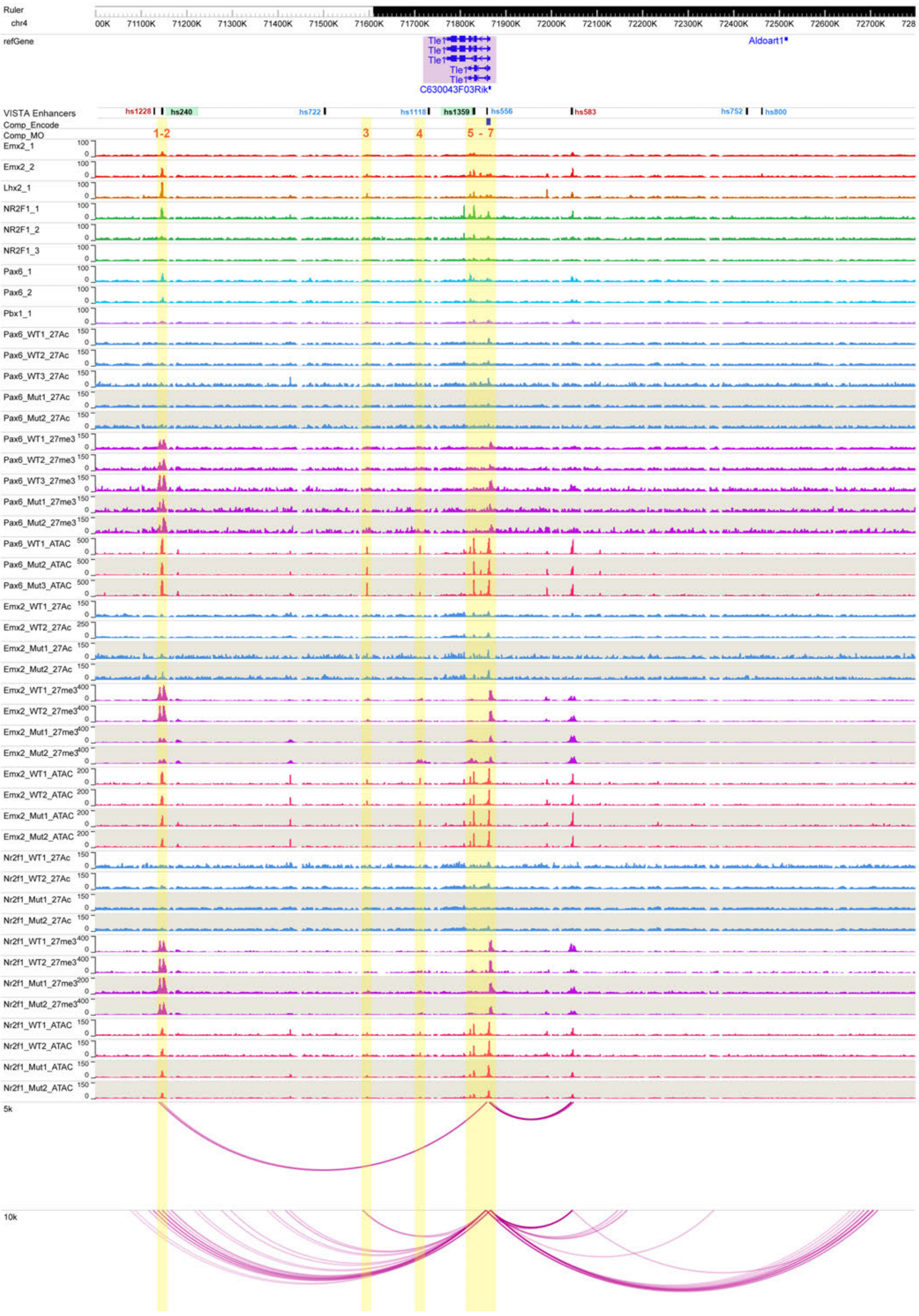

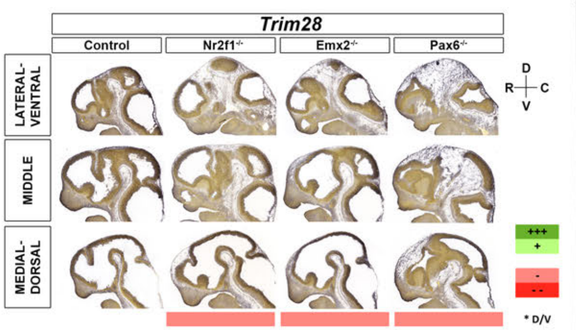

		Motifs					TF ChIP Seq					Differential epigenomic marks in WT vs. Mutant									Plac-Seq	Computation	Genomic features
												Pax6 mutant			Emx2 mutant			Nr2f1 mutant					
		Emx2 HB	Nr2f1	Pax6 Hb	Pax6 P	Pbx1	Emx2	Lhx2	Nr2f1	Pax6	Pbx1	27Ac	27me3	ATAC	27Ac	27me3	ATAC	27Ac	27me3	ATAC			
Trim28	chr7:13480000-13716381																						
1	chr7:13594337-13595650			x	x	x				x	x	Same		Same	Same		Same	Loss		Same			Zbtb45 TSS
2	chr7:13608554-13611167											Loss		Same	Same		Same	Same		Same			Trim28 TSS
3	chr7:13622814-13623925			x					x			Loss		Same	Same		Same	Loss		Same			Ube2m TSS

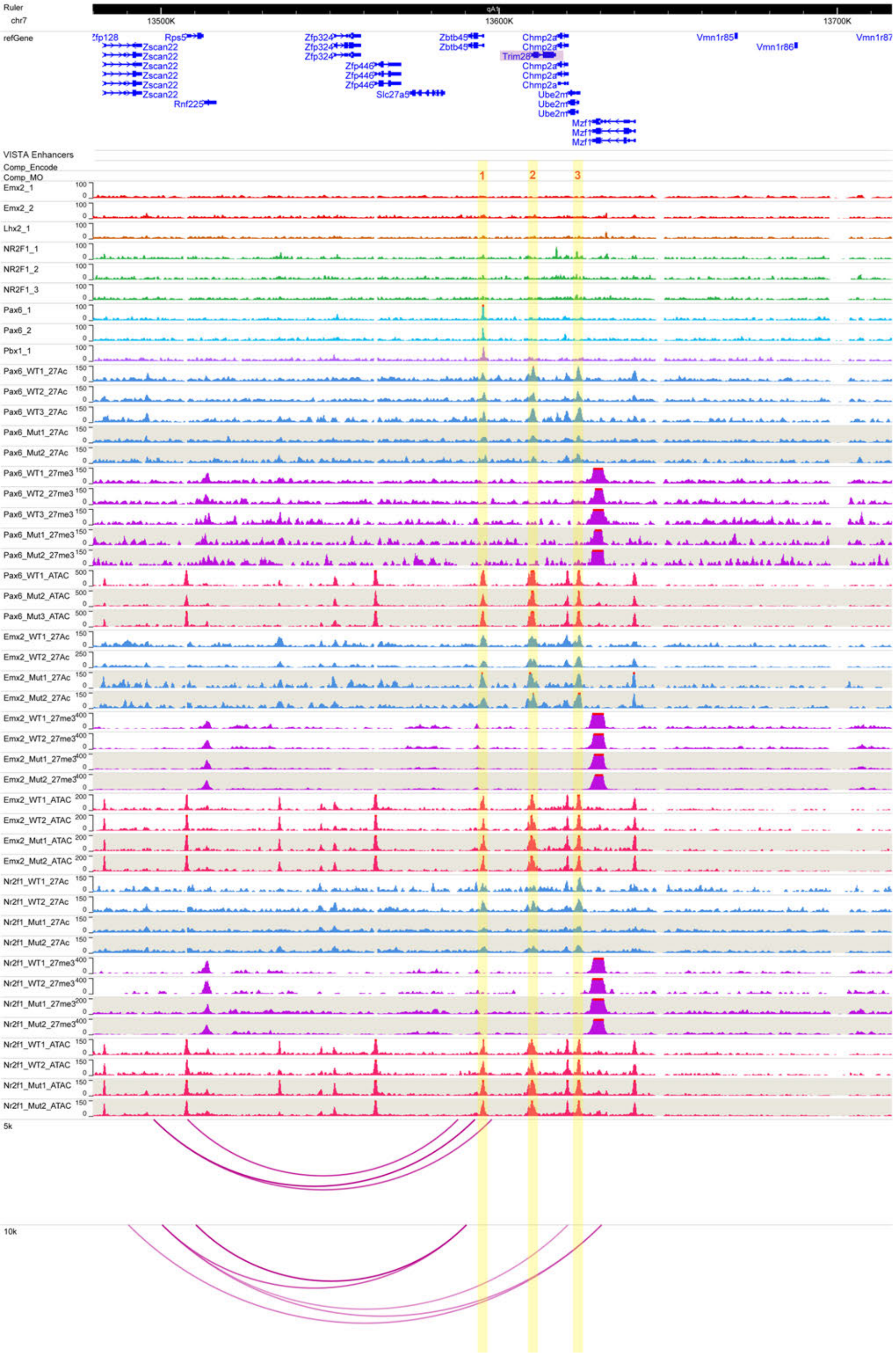

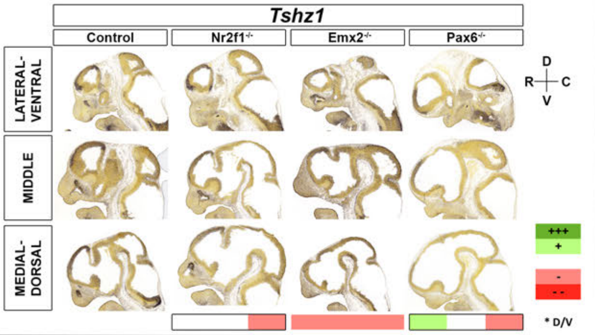

		Motifs					TF ChIP Seq					Differential epigenomic marks in WT vs. Mutant									Plac-Seq	Computation	Genomic features
												Pax6 mutant			Emx2 mutant			Nr2f1 mutant					
		Emx2 HB	Nr2f1	Pax6 Hb	Pax6 P	Pbx1	Emx2	Lhx2	Nr2f1	Pax6	Pbx1	27Ac	27me3	ATAC	27Ac	27me3	ATAC	27Ac	27me3	ATAC			
Tshz1	chr18:83200000-84800000																						
1	chr18:83568794-83569877								x					Loss			Same			Same	x		
2	chr18:83806342-83808046	x		x			x	x		x		Same		Same	Loss		Loss	Same		Same	x		
3	chr18:83982198-83985551	x					x						Same	Small Gain		Loss			Same		x		
4	chr18:84258352-84261915												Loss			Gain			Loss			x	Zadh2 Gene Body
5	chr18:84757934-84760102											Loss		Same	Same		Same	Loss		Same	x		Zfp407 TSS

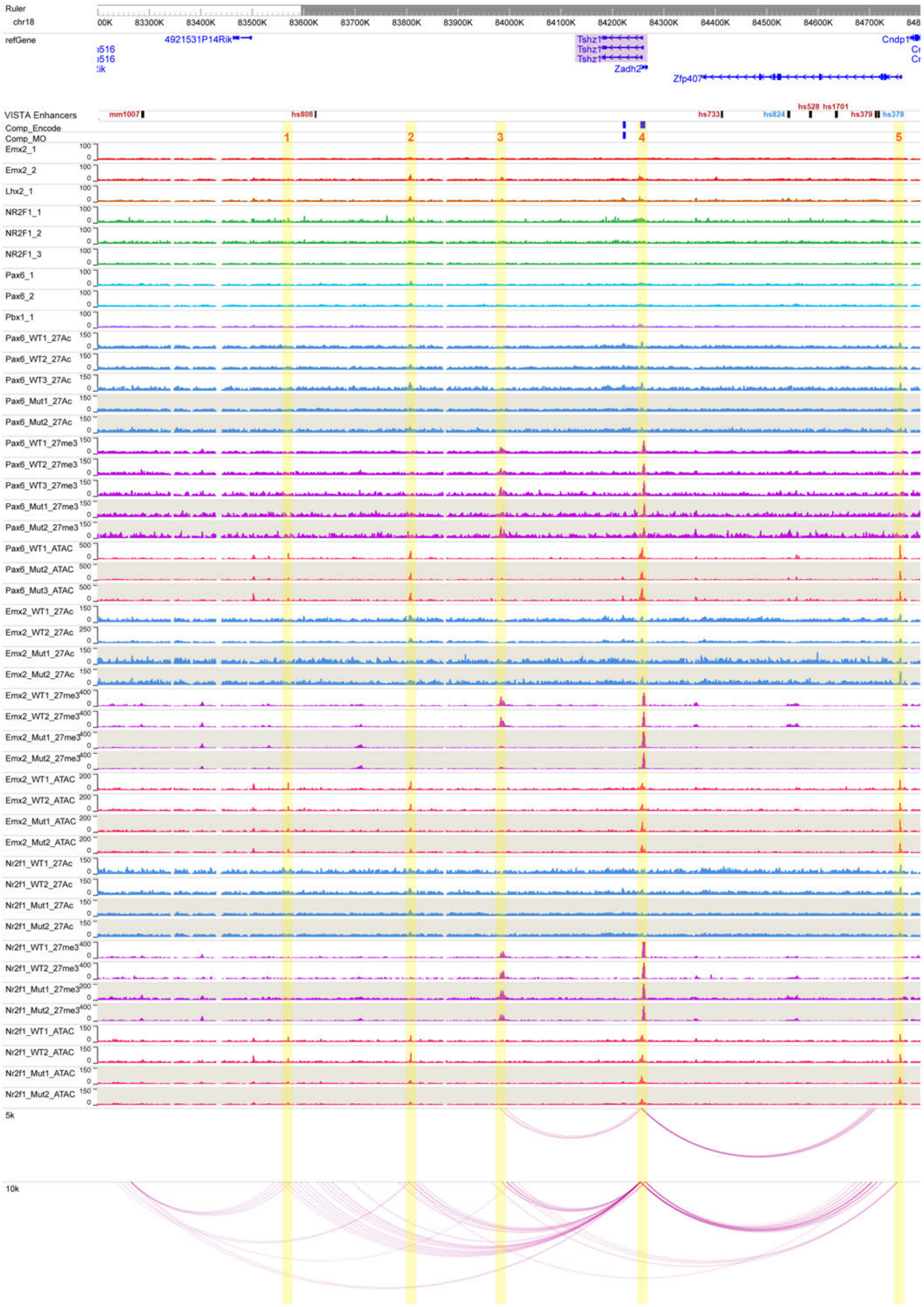

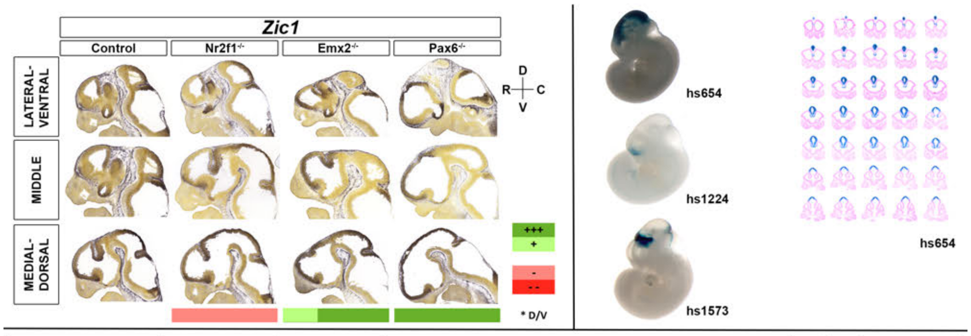

		Motifs					TF ChIP Seq					Differential epigenomic marks in WT vs. Mutant									Plac-Seq	Computation	Genomic features
												Pax6 mutant			Emx2 mutant			Nr2f1 mutant					
		Emx2 HB	Nr2f1	Pax6 Hb	Pax6 P	Pbx1	Emx2	Lhx2	Nr2f1	Pax6	Pbx1	27Ac	27me3	ATAC	27Ac	27me3	ATAC	27Ac	27me3	ATAC			
Zic1	chr9:90500000-91900000																						
1	chr9:90587642-90589755	x					x	x	x	x			Loss	Same		Same	Same		Same	Same	x	x	hs654
2	chr9:90595267-90596439	x					x	x		x				Loss	Gain		Same			Same	x		hs739
3	chr9:90625721-90627316									x				Loss			Gain			Same	x		hs1549
4	chr9:91243690-91252943												Same	Same	Gain	Loss	Gain		Same	Gain		x	
5	chr9:91254220-91260123											Same	Loss	Same	Gain	Loss	Gain	Loss	Loss	Same		x	Zic1 Gene body
6	chr9:91261560-91266186		x						x			Same	Same	Loss	Gain	Loss	Gain	Same	Loss	Same			hs1043 + Zic4 TSS
7	chr9:91271293-91279908									x		Same	Same	Same	Gain	Loss	Same	Same	Same	Same		x	Zic4 gene body
8	chr9:91297461-91302087	x		x						x			Same	Same	Gain	Loss	Same		Loss	Same			

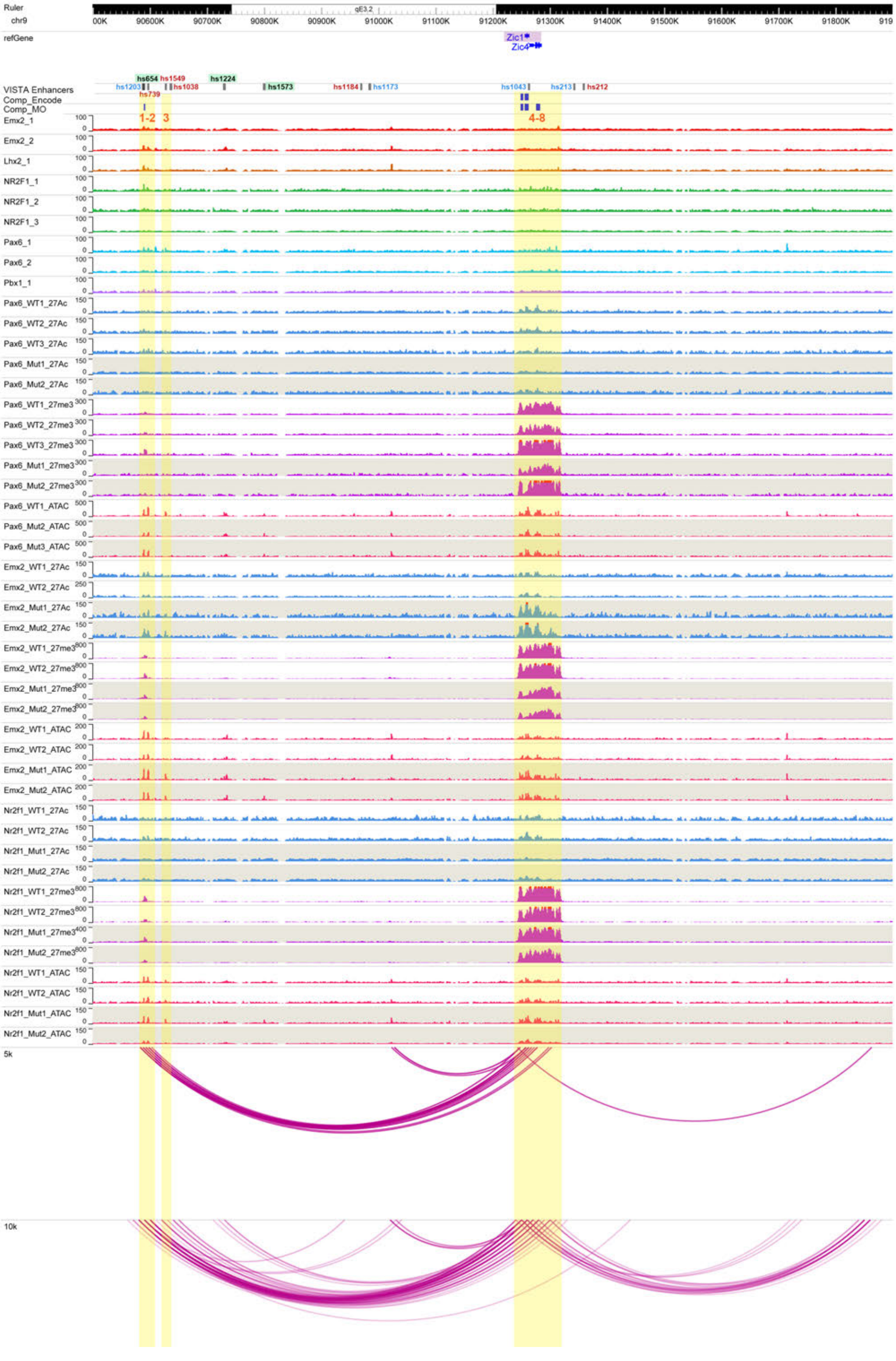

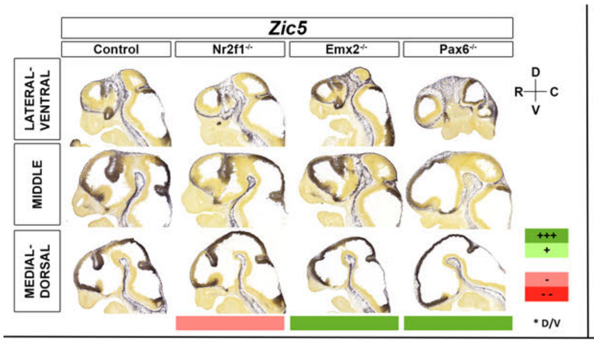

		Motifs					TF ChIP Seq					Differential epigenomic marks in WT vs. Mutant									Plac-Seq	Computation	Genomic features
												Pax6 mutant			Emx2 mutant			Nr2f1 mutant					
		Emx2 Hb	Nr2f1	Pax6 Hb	Pax6 P	Pbx1	Emx2	Lhx2	Nr2f1	Pax6	Pbx1	27Ac	27me3	ATAC	27Ac	27me3	ATAC	27Ac	27me3	ATAC			
Zic5	chr14:122500000-123750000																						
1	chr14:122625471-122629125			x	x		x		x						Gain						x	x	
2	chr14:122645738-122648063	x		x	x	x	x	x			x			Same		Small loss	Small loss			Same	x		
3	chr14:122772536-122775105	x					x		x		x			Same	Gain		Same			Same	x		
4	chr14:122861504-122864073								x			Loss		Same	Gain		Same	Same		Same			Zic5 TSS
5	chr14:122872971-122876529						x					Loss		Same	Gain		Same	Loss		Same			Zic2 TSS
6	chr14:123381259-123383393								x		x	Loss		Same	Gain		Same	Loss		Same	x		

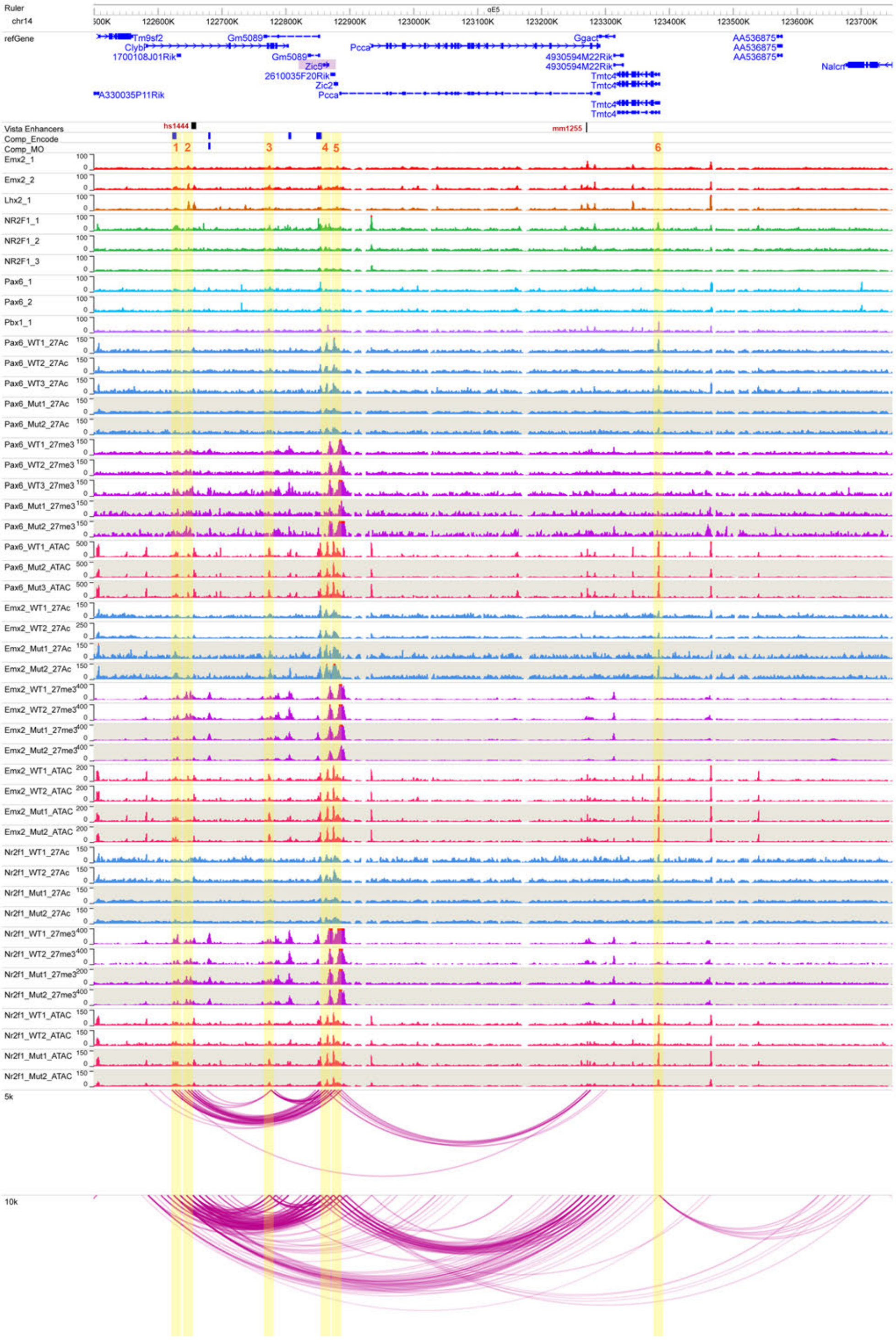

Figure S6. CRTFN transcriptional network in *Emx2*$^{-/-}$, *Nr2f1*$^{-/-}$ and *Pax6*$^{-/-}$
For each of the 38 TFs in the CRTFN, we show:

- ISH of TF gene in wild-type and mutant mice at E11.5.

- pallial VISTA enhancer wholemount (and sections if available) showing activity in the cortex at E11.5.

- Table with a comprehensive list of pREs that shows differential regulation in *Pax6*$^{-/-}$, *Emx2*$^{-/-}$ and *Nr2f1*$^{-/-}$ around the interactome of the relevant TF. Each pRE in this table is highlighted by a yellow column in the genome tracks below. The highlighted columns have orange numbers matched to the row number in this table.

- TF's genomic loci showing:

1. RefSeq genes around the TF gene (highlighted in purple).
2. Genomic position of VISTA enhancers with cortical activity (Green), activity in other regions (Blue) or no activity (Red) at E11.5.
3. Computationally derived enhancer-gene associations based on correlation between the putative enhancer activity (using Encode data: Comp_Encode or data generate here: Comp_MO) and the expression level of the genes residing in the same topologically-associating domain.
4. TF ChIP binding for EMX2, LHX2, NR2F1, PAX6 and PBX1; replicates are shown.
5. Epigenomic marks in VZ of Wild-Type (Shaded in white) and *Pax6*$^{-/-}$, *Emx2*$^{-/-}$ and *Nr2f1*$^{-/-}$ littermates (shaded in grey). For each experiment, H3K27ac tracks are in blue, H3K27me3 tracks are in purple, and ATAC-seq tracks are in red. Replicates are shown.
6. pRE-promoter interactions derived from PLAC-seq are shown by arcs (magenta) using two statistical stringencies (5k and 10k).

pREs with differential epigenomic marks in any one mutant are highlighted in yellow columns. These columns are labelled by orange numbers that correspond to the rows in the table above.

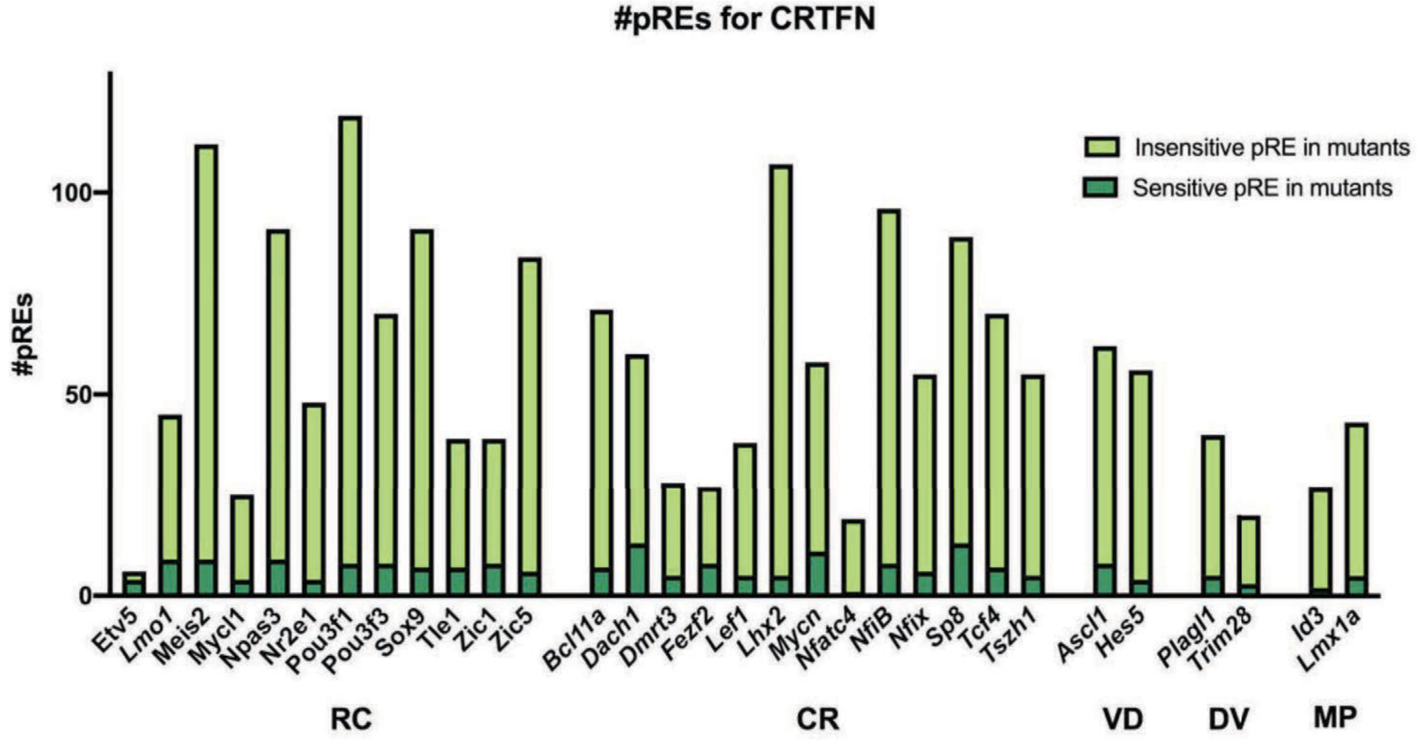

#pREs for CRTFN
Insensitive pRE in mutants
Sensitive pRE in mutants
#pREs
0
50
100
Etv5
Lmo1
Meis2
Mycl1
Npas3
Nr2e1
Pou3f1
Pou3f3
Sox9
Tle1
Zic1
Zic5
Bcl11a
Dach1
Dmrt3
Fezf2
Lef1
Lhx2
Mycn
Nfatc4
NfiB
Nfix
Sp8
Tcf4
Tszh1
Ascl1
Hes5
Plagl1
Trim28
Id3
Lmx1a
RC
CR
VD
DV
MP

Figure S7.
Bar plot showing proportion of pREs for CRTFN genomic loci that are sensitive (light green) or insensitive (dark green) to the effects of *Pax6*$^{-/-}$, *Emx2*$^{-/-}$ and *Nr2f1*$^{-/-}$.

DATASETS

DATASET S1: Annotation of TF expression in pallium at E11.5. Expression levels were assessed on a scale of 0-5 for density (D) and intensity (I) of ISH staining in subregions of the pallium: LVP, RDP, CDP, MP. When possible, VZ/SVZ and Mantle zone expression were assessed (blank means levels of staining in these regions could not be assessed).

DATASET S2: Genomic coordinates of CRTFN pRES annotated for TF ChIP-seq peaks (EMX2, LHX2, NR2F1, PAX6 and PBX1), histone ChIP-seq peaks (H3K27ac, H3K27me3) and ATAC-seq peaks. Each row is a pRE. Column A is the TF gene and column B-D is the interactome of a given TF gene. Column H is the size of the pRE. Numbers in column I are the number of TFs co-bound to a given pRE. Numbers in columns (J-Q) indicates how many replicates have a peak. Number of replicates for each ChIP-seq/ATAC-seq is indicated in parenthesis in the title of column. Column R annotates the presence of a VISTA enhancer.

DATASET S3: Classification of CRTFN VISTA enhancers as Pallial, Subpallial, Non-telencephalic and Inactive at E11.5. Table shows VISTA enhancer name, genomic coordinates, region of activity and VISTA annotation of other regions of activity, TF and histone ChIP-seq peaks. Table includes: enhancer name, genomic coordinates, size of enhancer, relevant region of activity of enhancer (P for Pallial, SP for Subpallial, N for Non-telencephalic and I for Inactive), other regions of activity of enhancer, TF ChIP-seq, WT histone marks (H3K27ac and H3K27me3) and ATAC-seq peaks. In rows, x indicates presence of that particular feature.

DATASET S4: Classification of gradients of activity in CRTFN VISTA enhancers. Table shows VISTA enhancer name, genomic coordinates, flanking genes, cortical gradient of activity (RC = rostrocaudal, CR = caudorostral, DV = dorsoventral, VD = ventrodorsal) and a detailed analysis of their subregional cortical expression of activity when sections were available (columns J-R). Number 1 in column indicates presence of that particular feature.

DATASET S5: PLAC-seq and computational pREs-promoter interactions in the interactome of CRTFN genes. Tab 1 maps the interactions derived from PLAC-seq in CRTFN interactomes using a 5k stringency. Computationally derived enhancer-gene associations are also indicated. This computational analysis is based on correlation between the putative enhancer activity (using Encode data: Comp_Encode or data generated here: Comp_MO) and the expression level of the genes residing in the same topologically-associating domain. Tab 2 is the same as Tab 1 with a stringency for PLAC-seq of 10k. Footnotes and abbreviations for Tab 1-2 is in Tab 3.

DATASET S6: Coordinates and activity annotations of newly tested VISTA enhancers. Each row is a newly tested VISTA enhancer and includes information about its coordinates, its activity overall, its cortical activity (column F), annotations of other regions of activity, annotations for presence of TF ChIP-seq, histone and ATAC ChIP-seq peaks, and pRE-promoter interactions. x and xx indicate presence of peaks in 1 or 2 replicates respectively.

DATASET S7: Coordinates and annotations (Gain, Loss, No Change) of pREs that show changes in histone marks and/or chromatin accessibility in *Pax6*$^{-/-}$, *Emx2*$^{-/-}$ and *Nr2f1*$^{-/-}$. This table shows for each interactome: coordinates of sensitive pREs (column B); primary DNA binding motifs (column C-G); TF ChIP-Seq (column H-L); Gain, Loss and No Change in Histone marks and ATAC Seq in Pax6, Emx2 and Nr2f1 mutants (Columns M-U); Plac-Seq and computation pRE-promoter binding (Column V-W); Genomic features of a given pRE.

SI References:

1. Hill RE, et al. (1991) Mouse small eye results from mutations in a paired-like homeobox-containing gene. *Nature* 354(6354):522–525.

2. Pellegrini M, Mansouri A, Simeone A, Boncinelli E, Gruss P (1996) Dentate gyrus formation requires Emx2. *Development* 122(12):3893–3898.

3. Zhou C, Tsai SY, Tsai MJ (2001) COUP-TFI: an intrinsic factor for early regionalization of the neocortex. *Genes & Development* 15(16):2054–2059.

4. Tang K, Rubenstein JLR, Tsai SY, Tsai M-J (2012) COUP-TFII controls amygdala patterning by regulating neuropilin expression. *Development* 139(9):1630–1639.

5. Gorski JA, et al. (2002) Cortical excitatory neurons and glia, but not GABAergic neurons, are produced in the Emx1-expressing lineage. *Journal of Neuroscience* 22(15):6309–6314.

6. Erbel-Sieler C, et al. (2004) Behavioral and regulatory abnormalities in mice deficient in the NPAS1 and NPAS3 transcription factors. *Proc Natl Acad Sci USA* 101(37):13648–13653.

7. Pattabiraman K, et al. (2014) Transcriptional Regulation of Enhancers Activein Protodomains of the Developing Cerebral Cortex. *Neuron* 82(5):989–1003.

8. Zhao Y, et al. (2008) Distinct molecular pathways for development of telencephalic interneuron subtypes revealed through analysis of Lhx6 mutants. *J Comp Neurol* 510(1):79–99.

9. Li H, Durbin R (2009) Fast and accurate short read alignment with Burrows-Wheeler transform. *Bioinformatics* 25(14):1754–60.

10. Ramírez F, et al. (2016) deepTools2: a next generation web server for deep-sequencing data analysis. *Nucleic Acids Research* 44(W1):W160–5.

11. Heinz S, et al. (2010) Simple combinations of lineage-determining transcription factors prime cis-regulatory elements required for macrophage and B cell identities. *Molecular Cell* 38(4):576–589.

12. Telley L, et al. (2016) Sequential transcriptional waves direct the differentiation of newborn neurons in the mouse neocortex. *Science* 351(6280):1443–1446.

13. Sandberg M, et al. (2016) Transcriptional Networks Controlled by NKX2-1 in the Development of Forebrain GABAergic Neurons. *Neuron* 91(6):1260–1275.

14. Fang R, et al. (2016) Mapping of long-range chromatin interactions by proximity ligation-assisted ChIP-seq. *Nature Publishing Group* 26(12):1345–1348.

15. Juric I, et al. (2019) MAPS: Model-based analysis of long-range chromatin interactions from PLAC-seq and HiChIP experiments. *PLoS Comput Biol* 15(4):e1006982.

16. Zhang Y, et al. (2008) Model-based analysis of ChIP-Seq (MACS). *Genome Biol* 9(9):R137.

17. Osterwalder M, et al. (2018) Enhancer redundancy provides phenotypic robustness in mammalian development. *Nature* 554(7691):239–243.

18. Gorkin DU, et al. (2020) An atlas of dynamic chromatin landscapes in mouse fetal development. *Nature* 583(7818):744–751.

19. Visel A, Minovitsky S, Dubchak I, Pennacchio LA (2007) VISTA Enhancer Browser--a database of tissue-specific human enhancers. *Nucleic Acids Research* 35(Database issue):D88–D92.

20. Speir ML, et al. (2016) The UCSC Genome Browser database: 2016 update. *Nucleic Acids Research* 44(D1):D717–D725.

21. Kothary R, et al. (1989) Inducible expression of an hsp68-lacZ hybrid gene in transgenic mice. *Development* 105(4):707–714.

22. Pennacchio LA, et al. (2006) In vivo enhancer analysis of human conserved non-coding sequences. *Nature* 444(7118):499–502.

23. Lindtner S, et al. (2019) Genomic Resolution of DLX-Orchestrated Transcriptional Circuits Driving Development of Forebrain GABAergic Neurons. *CellReports* 28(8):2048–2063.e8.

24. Monahan K, et al. (2017) Cooperative interactions enable singular olfactory receptor expression in mouse olfactory neurons. *eLife* 6. doi:10.7554/eLife.28620.

25. Fornes O, et al. (2020) JASPAR 2020: update of the open-access database of transcription factor binding profiles. *Nucleic Acids Research* 48(D1):D87–D92.
